# Deep ocean seascape and Pseudotanaidae (Crustacea: Tanaidacea) diversity at the Clarion-Clipperton Fracture Zone

**DOI:** 10.1038/s41598-019-51434-z

**Published:** 2019-11-21

**Authors:** Aleksandra Jakiel, Ferran Palero, Magdalena Błażewicz

**Affiliations:** 1 Department of Invertebrate Zoology and Hydrobiology, Faculty of Biology and Environmental Protection, University of Lodz, ul. Banacha 12/16, 90-237 Łódź, Poland; 2 Centre d’Estudis Avançats de Blanes (CEAB-CSIC), Carrer d’accés a la Cala Sant Francesc 14, 17300 Blanes, Spain; 3 Associate Researcher, Department of Life Sciences, The Natural History Museum, Cromwell Road, London, SW7 5BD UK

**Keywords:** Evolution, Zoology

## Abstract

Understanding the diversity and spatial distribution of benthic species is fundamental to properly assess the impact of deep sea mining. Tanaidacea provide an exceptional opportunity for assessing spatial patterns in the deep-sea, given their low mobility and limited dispersal potential. The diversity and distribution of pseudotanaid species is characterized here for the Clarion and Clipperton Fractures Zone (CCZ), which is the most extensive deposit field of metallic nodules. Samples were taken from the Belgian, German and French license areas, but also from the APEI 3 (Area of Particular Environmental Interest 3) of the Interoceanmetal consortium associates. The combination of morphological and genetic data uncovered one new pseudotanaid genus (*Beksitanais* n. gen.) and 14 new species of *Pseudotanais* (2 of them virtual taxa). Moreover, our results suggest that spatial structuring of pseudotanaid diversity is correlated with deep-sea features, particularly the presence of fractures and seamount chains crossing the CCZ. The presence of geographical barriers delimiting species distributions has important implications for the establishment of protected areas, and the APEI3 protected area contains only one third of the total pseudotanaid species in CCZ. The specimen collection studied here is extremely valuable and represents an important first step in characterizing the diversity and distribution of pseudotanaids within the Tropical Eastern Pacific.

## Introduction

The influence of habitat heterogeneity on species diversity has puzzled biologists for a long time and still raises many questions^1–3^. High habitat heterogeneity and spatial complexity provide shelter for many invertebrate taxa and might result in higher diversity of benthic organisms^4^. Competition and influence of predators are restricted in heterogeneous areas^5,6^ while the number of potential ecological niches increases^7^. Studies concerning benthic marine fauna have traditionally focused on shallow-water areas, so that knowledge on deep-sea habitat heterogeneity and its influence at various spatial scales is still lacking^8^. The deep-sea ecosystem was considered as a rather homogeneous environment in the past, but the application of state-of-the-art technologies for habitat mapping has proven otherwise^1^. McClain and Barry (2010)^9^ have shown that habitat heterogeneity is an important factor driving the structure of benthic assemblages and that significant species turnover can be observed at relatively small scales (<1 km)^8^. Abyssal hills increase habitat heterogeneity, benthic megafaunal biomass and diversity^10^. Furthermore, benthic meiofauna studies also show that deep sea nodule fields facilitate the coexistence of species with different modes of life, ranging from sediment dwelling to epifaunal^11^.

The Clarion and Clipperton Fractures Zone (CCZ) is a 6 million km^2^ region located in international waters of the Tropical Eastern Pacific. Well-known to mining corporations, this is the most extensive deposit field of metallic nodules, rich in manganese, nickel, copper and cobalt^12,13^. The attraction for deep sea nodules has raised in the last few years because they host large quantities of other critical metals needed for high-tech, green-tech, and energy applications^14^. The exploration and exploitation of the CCZ is currently managed by the International Seabed Authority (ISA), an intergovernmental body that regulates mining and related activities in the seabed beyond national jurisdiction^15^. ISA has recently granted 15 mining licences in the CCZ area and selected 9 Areas of Particular Environmental Interest (APEI) as non-mining, reference areas. Fields rich in polymetallic nodules represent heterogeneous habitats, which increases regional diversity^11,16,17^, but removing nodules, together with the resuspension and redeposition of the sediment, affects local fauna^18^. Experimental work suggests that mining may cause major disturbances on nodule-associated fauna and reduce biodiversity^19^. Therefore, understanding connectivity and spatial distribution of benthic species is fundamental to properly assess the impact of mining^20^.

Tanaidacea are small peracarid crustaceans, benthic brooders, living on tubes or buried in the sediment. Tanaidacean abundance is usually underestimated^21,22^, but they can be more numerous than amphipods or isopods^23^. They have low mobility and limited dispersal potential, and provide an exceptional opportunity for assessing connectivity patterns in the deep-sea. Morphological identification of tanaidaceans is difficult because of their small size and sexual dimorphism^23^, and some currently accepted taxa might form in fact species complexes, considering their low dispersal abilities and reproductive biology^24^. The use of molecular techniques before thorough morphological evaluation (i.e. reverse taxonomy) can be advantageous when the occurrence of cryptic species is expected^25,26^. Nevertheless, the scarcity of data in public databases such as GenBank or BOLD is a limiting factor for the study of genetic variation in Tanaidacea. From a total of 346 tanaid sequences deposited in GenBank, ~25% are simply identified as ‘unclassified Tanaidacea’, which clearly hinders the use of DNA barcoding approaches. This is particularly pressing on the Pseudotanaidae, for which the only sequence available in public databases corresponds to the Histone 3 gene of *Pseudotanais* sp^27^, and without any DNA barcoding data published so far.

Pseudotanaidae (Sieg 1976) species represent a frequent and diverse element of deep-sea benthic assemblages, only exceeded by polychaetes^28,29^. The genus *Pseudotanais* is the most speciose within the family, formed by four species-groups: ‘affinis’, ‘denticulatus’, ‘forcipatus’ and ‘longisetosus’, based on morphological variation in key traits (e.g. antenna article 2–3, mandibles, chelipeds, and setation and ornamentation on pereopods 1–3) (see^30^ and^31^). However, the validity of these groups is unclear and the systematics of pseudotanaids has never been studied using molecular methods. From the 55 pseudotanaid species known, only 9 have been reported from the Pacific Ocean, 7 restricted to this area (*Akanthinotanais makrothrix* Dojiri and Sieg, 1997; *Pseudotanais californiensis* Dojiri and Sieg, 1997; *P*. *abathagastor* Błażewicz-Paszkowycz *et al*., 2013; *P*. *intortus* Błażewicz-Paszkowycz *et al*., 2013; *P*. *soja* Błażewicz-Paszkowycz *et al*., 2013; *P*. *nipponicus* McLelland, 2007 and *P*. *vitjazi* Kudinova-Pasternak, 1966; WoRMS 2018) and two species originally described from the Atlantic Ocean namely, *P*. *affinis* Hansen, 1887 and *P*. *nordenskioldi* Sieg, 1977 (reported by Kudinova-Pasternak^31^ but unlikely to belong to these two Atlantic species).

The present study was designed to characterize the diversity and distribution of pseudotanaid species in the CCZ area. The mitochondrial gene coding for the subunit I of the cytochrome oxidase was selected to help filling the current gap in molecular databases. The combination of morphological and molecular genetic data uncovered the presence of one new genus (*Beksitanais* n. gen.) and 14 new species of *Pseudotanais* (two of them virtual taxa). Moreover, our results suggest that genetic structuring of pseudotanaid diversity is correlated with deep-sea landscape and the presence of seamounts and fractures crossing the CCZ.

## Results

### Phylogenetic analyses

**Figure 1:**
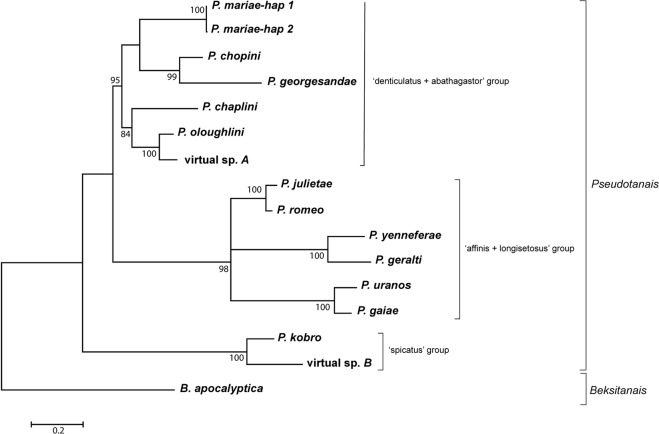
Evolutionary relationships between Pseudotanaidae species inferred by using the COI sequences and the Maximum Likelihood method. The percentage of trees in which the associated taxa clustered together (bootstrap support) is shown next to the branches. Only values above 70% are shown.

Pseudotanaids were found in 87% (13 out of 15) of the stations surveyed, which confirms the generalized presence of these tanaids in the deep-sea benthos (Table 1). The bathymetric range where pseudotanaids were captured was large, spanning from 4093 m to 4877 m depth. A total of 67 individuals were used for molecular analysis and gave positive DNA barcoding results (Table 2). A total of 16 different COI haplotypes were obtained (Fig. 1), representing one *Beksitanais* and 14 *Pseudotanais* species (two virtual taxa, without a voucher left for morphological analysis). The sequence alignment spanned 691 bp before trimming and was reduced to 611 bp after running Gblocks. The Hasegawa-Kishino-Yano (HKY + *G* + *I*) model showed the lowest BIC score (BIC = 9947.97) and it is considered to describe the substitution pattern the best. Non-uniformity of evolutionary rates among sites was modelled using a Gamma distribution (+*G* = 0. 85) and the rate variation model allowed for some positions to be evolutionarily invariable (+*I* = 37.61% sites). The Maximum Likelihood tree with the highest log likelihood value (lnL = −4841.74) is shown in Fig. 1. *Pseudotanais* species grouped into three well-supported clades namely, 1) the ‘spicatus’ group (including *P*. *kobro* and virtual species B); 2) the ‘affinis + longisetosus’ group (including three pairs of sister taxa: *P*. *romeo/P*. *julietae*, *P*. *geralti/P*. *yenneferae* and *P*. *uranos/P*. *gaiae*) and 3) the ‘abathagastor + denticulatus’ group (including *P*. *mariae*, the sister species *P*. *chopini/P*. *georgesandae* and a clade formed by *P*. *chaplini*, *P*. *oloughlini* and virtual species A). The genetic clustering of COI sequences in the ML tree corresponds to the morphological identification of taxa (see below).

**Table 1:** Pseudotanaidae presence (✓) or absence (×) on the surveyed stations.

Licence area	Station	Latitude [N]	Longitude [W]	Depth [m]	Pseudotanaid presence
BGR	20	11° 49.81′	117° 00.28′	4093	✓
BGR	24	11° 51.52′	117° 01.19′	4100	✓
BGR	50	11° 49.92'	117° 29.31'	4330	✓
BGR	59	11° 48.55'	117° 29.03′	4342	✓
IOM	81	11° 03.97'	119° 37.67'	4365	✓
IOM	99	11° 02.61'	119° 39.52'	4401	✓
GSR	117	13° 52.39'	123° 15.30′	4496	✓
GSR	133	13° 50.98′	123° 15.07′	4507	✓
IFREMER	158	14° 03.41′	130° 07.99'	4946	✓
IFREMER	171	14° 02.68′	130° 05.97'	5030	×
APEI3	192	18° 44.81′	128° 21.87'	4877	✓
APEI3	197	18° 48.66'	128° 22.75′	4805	✓
APEI3	210	18° 49.27′	128° 25.80′	4700	×

APEI3: Area of Particular environmental Interest 3; BGR: Bundesanstalt fur Geowissenschalfen und Rofstoffe (Germany); IOM: Interoceanometal; GSR: Global Sea Mineral Resources NV (Belgium); IFREMER: Institut Français de Recherche pour l’Exploitation de la Mer (France).

**Table 2:** Pseudotanaidae species abundance on the CCZ stations surveyed.

Area	Station	*B. apocalyptica*	*P. uranos*	*P. gaiae*	*P. yenneferae*	*P. geralti*	*P. julietae*	*P. romeo*	*P. georgesandae*	*P. oloughlini*	*virtual sp A*	*P. chaplini*	*P. mariae*	*P. chopini*	*P. kobro*	*virtual sp B*
BGR	20							1				1	1	3		1
	24							4						4		
	50													3		
	59													2	1	
IOM	81	3				2							2		1	
	99					4							1	1	3	
GSR	117					1					1				1	
	133						1									
IFREMER	158											1				
	171															
APEI3	192			3	1				1	2						
	197		5		9					3

Pairwise genetic *p-*distances between COI sequences ranged between 0 and 35.5% (Table S1). Intraspecific genetic variation was very low, as expected given the limited sample size per species, and only *P*. *mariae* showed more than one haplotype. Estimates of average evolutionary divergence over sequence pairs within groups of *Pseudotanais* species showed similar mean divergences within the ‘abathagastor + denticulatus’ group (0.228 ± 0.022) and within the ‘affinis + longisetosus’ group (0.277 ± 0.030), and lower divergences within the ‘spicatus’ group (0.060 ± 0.008). Net evolutionary divergences over sequence pairs between groups of species were larger between *Beksitanais* and any *Pseudotanais* clade than between *Pseudotanais* species groups. Within *Pseudotanais*, the ‘spicatus’ group and either the ‘affinis + longisetosus’ (0.429 ± 0.051) or the ‘abathagastor + denticulatus’ (0.402 ± 0.055) clades show divergences almost twice as large as those observed between ‘abathagastor + denticulatus’ and ‘affinis + longisetosus’ (0.275 ± 0.037).

### Spatial modelling and genetic gradients

**Figure 2:**
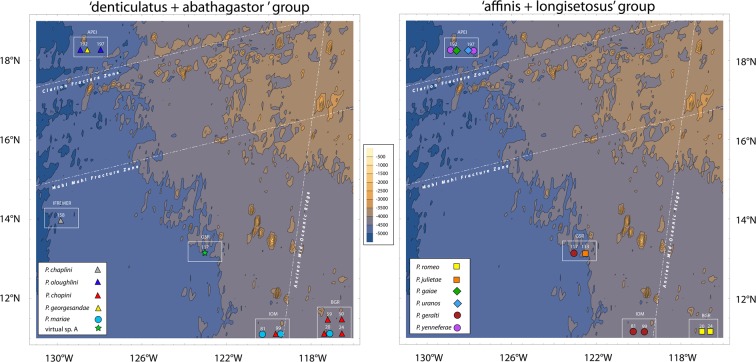
Contour plot showing the bathymetry of the studied area and the spatial distribution of the newly described Pseudotanaidae. Station numbers are shown in white. Mountain chains can be identified as a series of concentric contours running adjacent to the Clarion Fracture Zone or the ancient Mid-Ocean Ridge.

**Figure 3:**
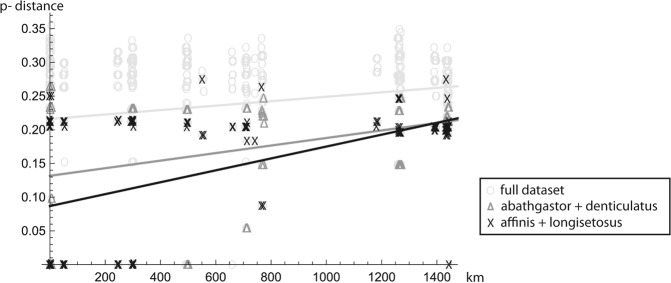
Correlation between genetic and geographic distances for the Pseudotanaidae species sampled. Symbols indicate comparison between all taxa (O), between samples from the ‘affinis + longisetosus’ clade (X) or between samples from the ‘abathagastor + denticulatus’ clade (Δ).

The 3D-model based on mean sea level data reveal an extremely heterogeneous deep sea landscape at the CCZ, with the presence of several seamounts and knolls (Fig. 2). In fact, two underwater mountain chains cross the studied area: one rise running east-to-west around latitude 17°N and another running south-southwest around longitude 120°W. The first isolates the APEI3 area (located around 18°N) from the remaining sampling sites, and includes seamountains about 4000 m high, reaching to 250 m under the surface (see Discussion). The second runs over the IOM area and separates the BGR area (located around 117°W) from the rest. Plotting the distribution of the newly identified taxa on the 3D spatial model revealed several species (*P*. *oloughlini*, *P*. *yenneferae*, *P*.*georgesandae* and the sister species *P*. *gaiae* and *P*. *uranos*) to be restricted to the APEI3 area. Another group of species were only found in the BGR and/or IOM areas (*P*. *romeo*, *P*. *mariae*, *B*. *apocalyptica*, virtual *Pseudotanais* sp. B and *P*. *chopini*). The virtual *Pseudotanais* sp. A, *P*. *julietae*, *P*. *geralti* and *P*. *kobro* were found together in the GSR area, although *P*. *kobro* was also collected in the BGR and IOM areas, and *P*. *geralti* was also found in the IOM area. The Spearman rank coefficient revealed a significant correlation between geographical and genetic distances for the complete dataset (ρ = 0.046; p-value = 0.032), and this spatial correlation was even higher when each well-supported phylogenetic clade ‘affinis + longisetosus’ (ρ = 0.121; p-value = 0.009) or ‘abathagastor + denticulatus’ (ρ = 0.224; p-value ≤ 0.001) was analysed independently. The linear fitting of an isolation by distance model gave similar results, with the genetic gradient being two times (for the ‘affinis + longisetosus’ clade) or even three times (for the ‘abathagastor + denticulatus’ clade) steeper than for the global dataset (Fig. 3).

### Morphological analyses and species description

**Family**: Pseudotanaidae Sieg, 1976

**Diagnosis**: Following Bird & Holdich (1989) and McLelland (2008), Pseudotanaidae can be characterized by: Eyelobes pigmented, rudimentary or absent. Medium body calcification. Pereon with six free pereonites, first reduced in length. Pleon with five free pleonites. Antennule with three articles. Antenna with six articles, articles 2 and 3 with or without stout spiniform setae. Mandible pars molaris broad or narrow, with or without terminal setation. Maxillule palp terminating with two setae and endite terminating with usually nine spiniform setae (two exceptions). Maxilla rudimentary. Maxilliped bases completely fused and endites completely or partially fused and bearing simple setae, cusps, or naked. Cheliped attached to body via sclerite. Chelae forcipate or not. Cheliped carpus with usually two inferior setae (three exceptions). Cheliped fixed finger usually with one inferior setae (four exceptions). Cheliped proximal dactylus seta present or absent. Marsupium formed by one pair of oostegites. Pereopods 2 to 6 carpus with or without modified blade-like setae. Pereopods 4 to 6 ischium with one or two setae, merus with one or two setae and dactylus fused with unguis forming claw. Pleopods usually elongate with terminal setae only (three exceptions). Uropod exopods and endopods with one or two articles or one pseudo-articulate article.

**Genus**: *Beksitanais* n. gen.

**Diagnosis**: Antennula article-3 with thickened rod seta. Antenna article 2 and 3 with seta; article-6 without thickened rod seta. Maxiliped palp article-4 without thickened rod seta. Chela forcipate with serrate incisive margin, propodus (palm) without small folds in distodorsal corner, cheliped with one interior seta on fixed finger. Pereopods 4–6 dactylus and unguis fused with a small hook on tip. Uropod exopod with one article, 0.5x endopod, endopod with pseudoarticulation.

Type species: *Beksitanais apocalyptica* n. sp.

**Etymology**: The genus is named to honour the famous Polish painter Zdzisław Beksiński.

Remarks: Beksitanais n. gen. is most similar to *Mystriocentrus*, but the presence of a thick rod seta on antennule article-3, lack of folds on distodorsal corner of the cheliped, absence of thick rod seta on antenna article-6, as well as lack of thick rod seta on maxilliped palp article-4 allow to distinguish both genera. *Beksitanais* can be separated from the genus *Akanthinotanais* by presence of blade-like spine on carpus of pereopod 2 and 3 and a forcipate chela. From the genus *Parapseudotanais* it can be distinguished by the presence of one interior seta on fixed finger and exopod uropod with one article only. Serrate inner margin on fixed finger and relative length of propodus of pereopod-1 allow to differentiate *Beksitanais* from *Pseudotanais*.

***Beksitanais apocalyptica*** **n**. **sp**.

Figures 4–8.

**Figure 4:**
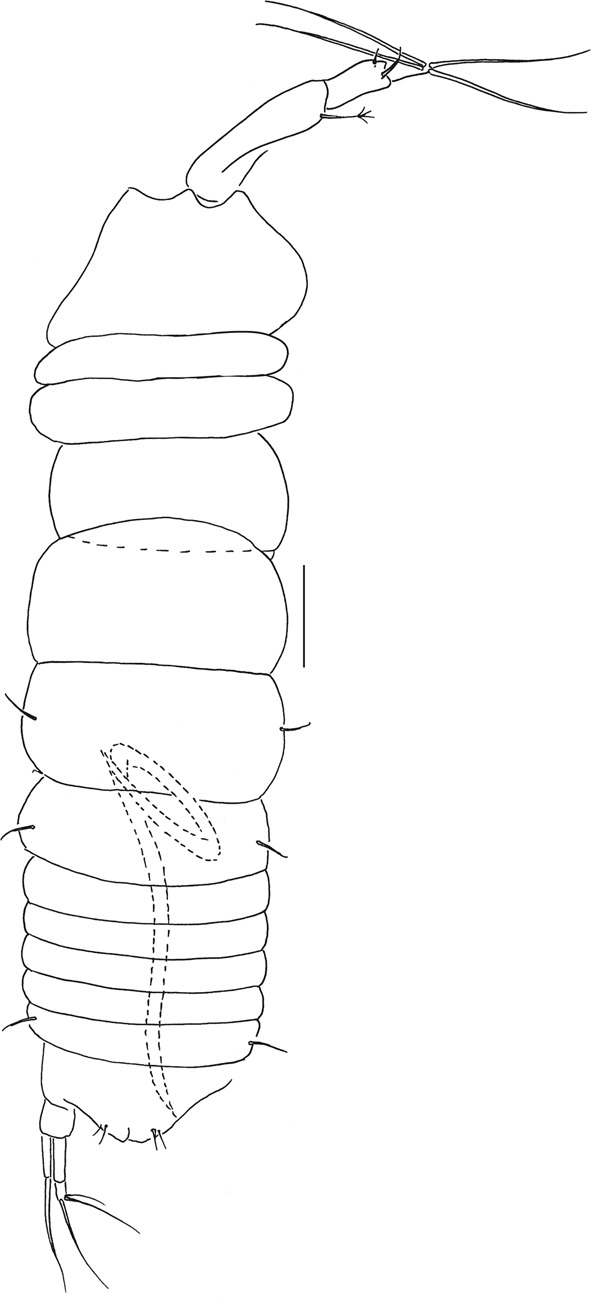
*Beksitanais apocalyptica* n. sp., ZMH K-56558, holotype, neuter, dorsal view in distal part of the animal parasitic nematode is observed. Scale bar: 0.1 mm.

**Figure 5:**
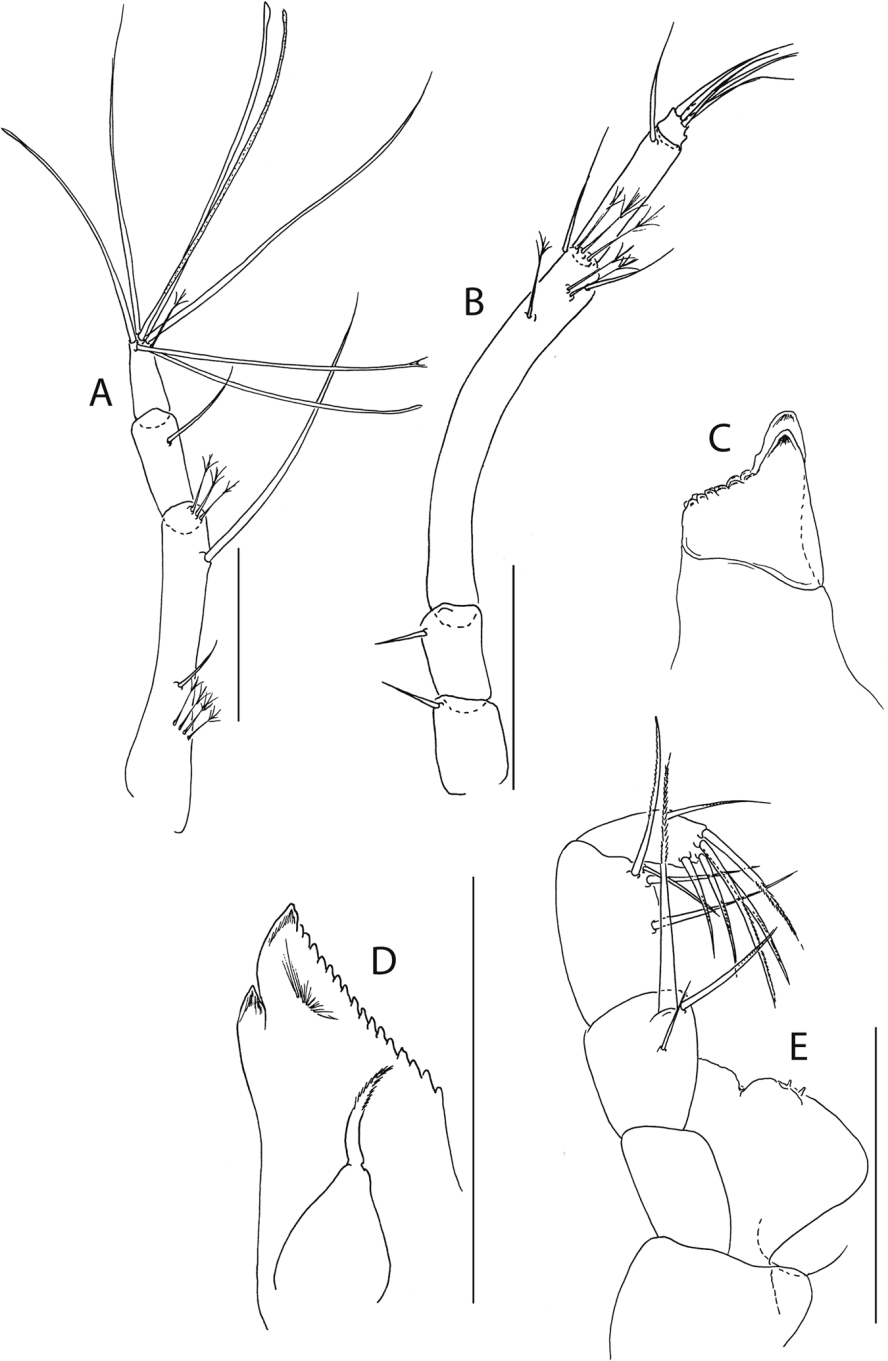
*Beksitanais apocalyptica* n. sp., ZMH K-56559, neuter. Mouthparts. (**A**), antennule; (**B**), antenna; (**C**), left mandible; (**D**), rigth mandible; (**E**), maxilliped. Scale bar: 0.1 mm.

**Figure 6:**
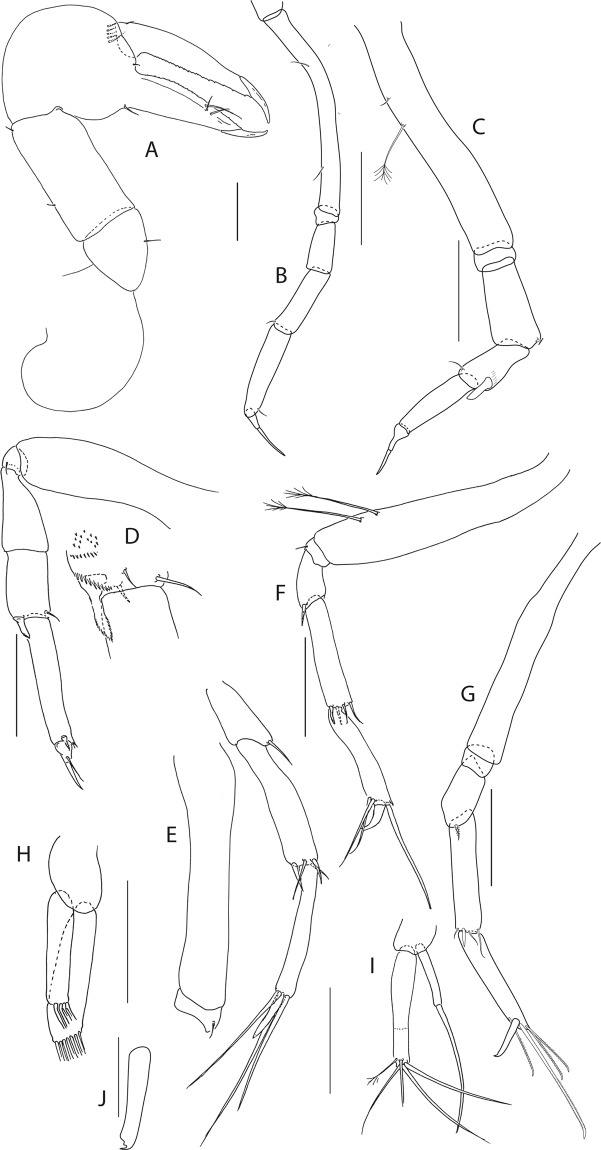
*Beksitanais apocalyptica* n. sp., ZMH K-56559, neuter. (**A**), cheliped; (**B**), pereopod-1; (**C**), pereopod-2; (**D**), pereopod-3; (**E**), pereopod-4; (**F**), pereopod-5; (**G**), pereopod-6; (**H**), pleopod; (**I**), uropod; (**J**), magnified dactylus and unguis for pereopods 4–6. Scale bars: 0.1 mm.

**Figure 7:**
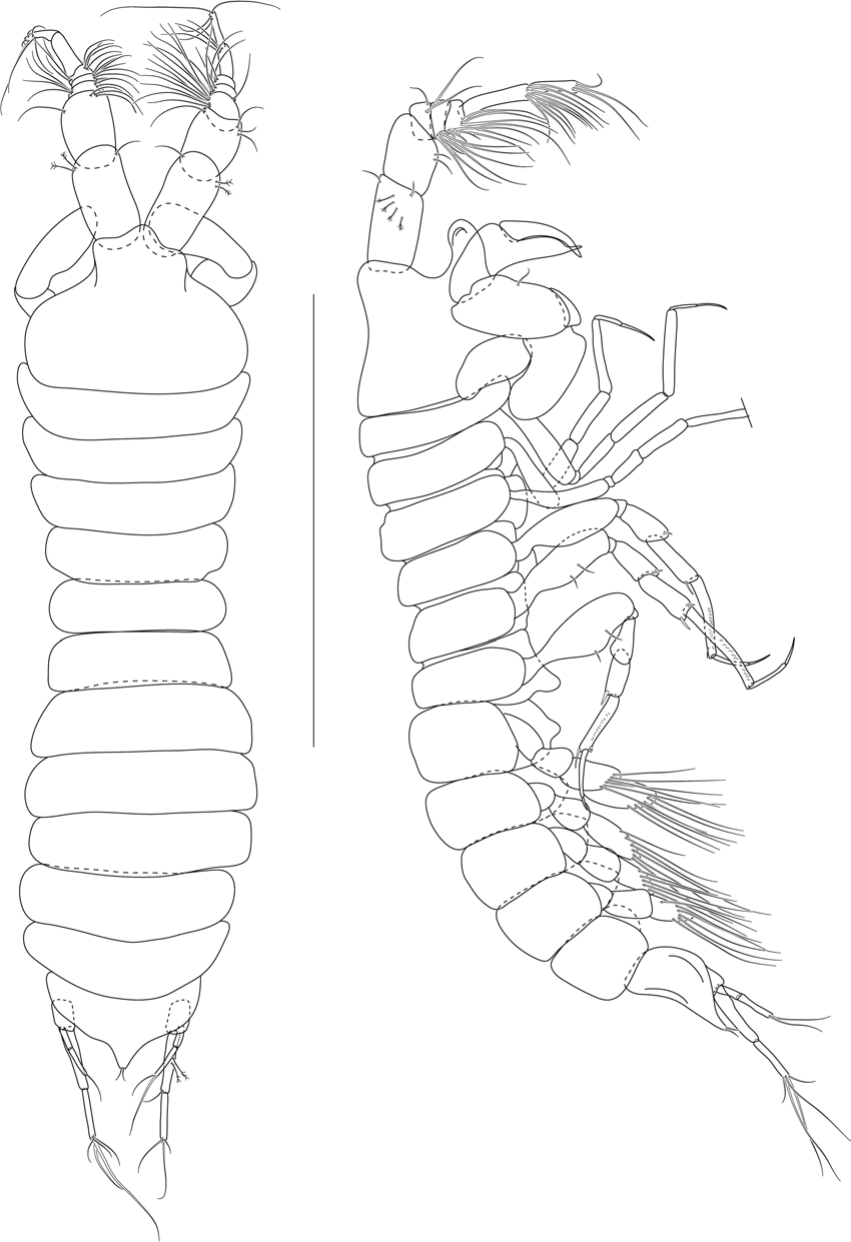
*Beksitanais apocalyptica* n. sp., ZMH K-56556, male. A, dorsal view; B, lateral view. Scale bar: 1 mm.

**Figure 8:**
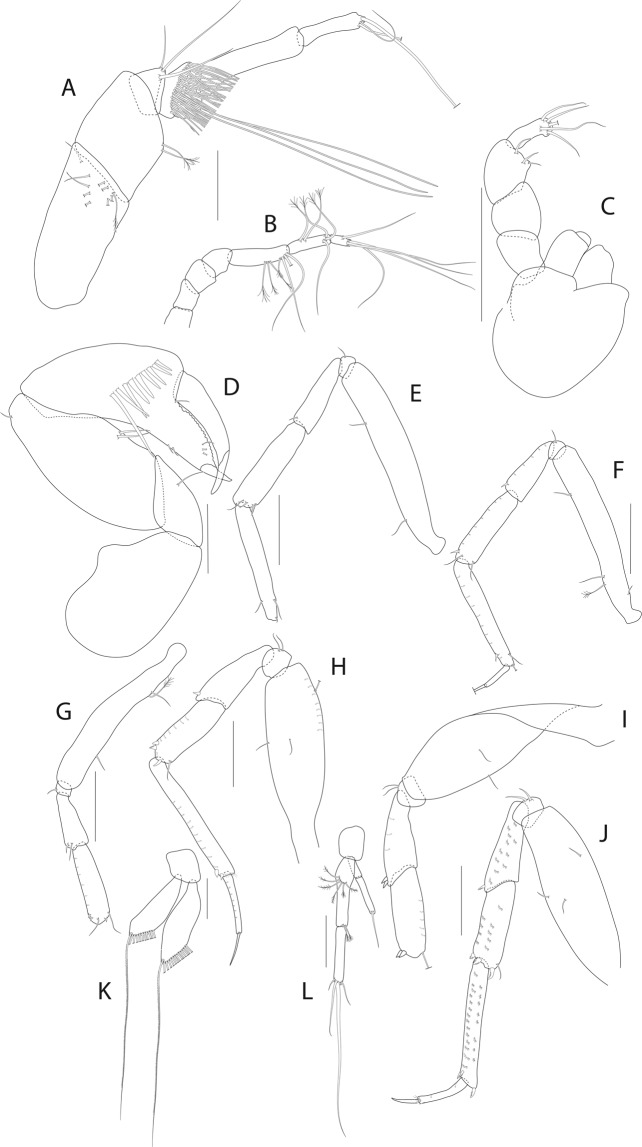
*Beksitanais apocalyptica* n. sp., ZMH K-56556, male. (**A**), antennule; (**B**), antenna; (**C**), maxilliped; (**D**), cheliped; (**E**), pereopod-1; (**F**), pereopod-2; (**G**), pereopod-3; (**H**), pereopod-4; (**I**), pereopod-5; (**J**), pereopod-6; (**K**), pleopod; (**L**), uropod. Scale bars: 0.1 mm.

Material examination. Holotype: neuter, BL = 0.9 mm, ZMH K-56558. St. 81, 11° 3.97′N, 119° 37.67′W, 4365 m, EBS, 1 Apr 2015.

Paratypes: two neuters, BL = 0.8 mm (one dissected), ZMH K-56557.

ZMH K-56558, ZMH K-56559 (dissected): adult (swimming male), BL = 1.8 mm (dissected), ZMH K-56556. St. 81, 11° 3.97′N, 119° 37.67′W, 4365 m, EBS, 1 Apr 2015; neuter, BL = 1 mm (dissected), ZMH K-56562. St. 128, 13° 51,10′N 123° 15,12′W, 4510.7 m, Box Core, 9 Apr 2015; two mancas, ZMH K-56560, ZMH K-56561. St. 137, 13° 51,36′N 123° 14,28′W, 4509 m, Box Core, 11 Apr 2015.

**Diagnosis:** Antenna article-6 and maxilliped palp article-4 without thickened rod seta. Uropod exopod with one article, 0.5x endopod; endopod with pseudoarticulation.

**Etymology:** The species is named by one of the period of artwork of Zdzisław Beksiński suffused by the post-apocalyptic images.

**Description of neuter**. BL = 0.9 mm. Body robust (Fig. 4), 3.9 L:W. Carapace 0.7 L:W, 3.6x pereonite-1, 0.2x BL. Pereonites 0.6x BL, pereonites-1–6: 0.2, 0.2, 0.5, 0.6, 0.5 and 0.4 L:W, respectively. Pleon short, 0.2x BL. Pleonites 0.8 L:W.

Antennule (Fig. 5A) article-1 0.6x total length, 7.0 L:W, 2.6x article-2, with one simple, four penicillate mid-length setae, strong subdistal seta and three penicillate distal setae; article-2 3.0 L:W, 1.4x article-3, with subdistal seta; article-3 2.4 L:W, with five simple, one bifurcate and one penicillate setae, and one aestetasc.

Antenna (Fig. 5B) article-1 1.2 L:W; article-2 0.8x article-3, with seta 0.7x the article; article-3 1.8 L:W, 0.2x article-4, with seta 0.5x the article; article-4 8.8 L:W, 2.7x article-5, with one simple and three penicillate subdistal setae, one simple and three penicillate setae distally; article-5 4.1 L:W, 5.8x article-6, with seta; article-6 0.8 L:W, with four setae.

Mouthparts. Left mandible (Fig. 5C) *lacinia mobilis* well developed, distally serrate, incisor distal margin serrate. Right mandible (Fig. 5D) incisor distal margin serrate, *lacina mobilis* merged to small process. Maxilliped (Fig. 5E) basis 0.7 L:W; endites partly merged, distal margin with two tubercles (gustatory cusps); article-2 inner margin with three setae; article-3 with three inner setae, article-4 with six setae: one subdistal, five distal.

Cheliped (Fig. 6A) slender; basis 1.3 L:W; merus with ventral seta; carpus 2.1 L:W, with dorso-distal and dorsosubproximal setae; chela forcipate; palm 1.2 L:W, with row of five setae on inner side; fixed finger distal spine pointed, regular size, with three ventral setae; dactylus 6.3 L:W, cutting edge serrate, proximal seta present.

Pereopod-1 (Fig. 6B) basis 10.4 L:W, 4.3x merus with two simple setae dorsally; ischium naked; merus 2.4 L:W, 0.7x carpus naked; carpus 3.4 L:W, 0.7x propodus, with one simple seta; propodus 5.4 L:W, 1.8x dactylus and unguis combined length, with one simple seta; dactylus 0.5x unguis.

Pereopod-2 (Fig. 6C) basis 6.5 L:W, 3.1x merus with one simple and one penicilate seta dorsally; ischium naked; merus 1.8 L:W, 1.3x carpus, with one simple seta; carpus 1.8 L:W, 0.7x propodus, with one simple seta and one blade-like spine, 0.3x propodus; propodus 4.2 L:W, 1.5x dactylus and unguis combined length; dactylus 1x unguis.

Pereopod-3 (Fig. 6D) basis broken; ischium with ventral seta; merus 2.1 L:W, 1.2x carpus naked; carpus 1.7 L:W, 0.5x propodus, with three simple and blade-like spine, 0.2x propodus; propodus 5.4 L:W, 2.5x dactylus and unguis combined length, with one spine; dactylus 0.7x unguis, dactylus with simple seta.

Pereopod-4 (Fig. 6E) basis 5.6 L:W, 3.5x merus; ischium with seta; merus 1.8 L:W, 0.5x carpus, with serrate seta; carpus 4.1 L:W, 1x propodus, with two simple setae, one rod seta 0.2x propodus, and one blade-like spine 0.2x propodus; propodus 6.6 L:W, 2.5x dactylus and unguis combined length, with three setae; dactylus and unguis fused with a small hook on tip.

Pereopod-5 (Fig. 6F) basis 5.6 L:W, 5.0x merus, with two ventral penicillate setae; ischium with ventral seta; merus 1.8 L:W, 0.4x carpus, with seta; carpus 5.0 L:W, propodus, with two simple setae, one rod seta 0.2x propodus, and one blade-like spine 0.3x propodus; propodus 4.8 L:W, 2.9x dactylus and unguis combined length, with two setae on ventral and seta on dorsal margin; dactylus and unguis fused with a small hook on tip.

Pereopod-6 (Fig. 6G) basis 7.5 L:W, 43.5x merus; ischium naked; merus 2.4 L:W, 0.6x carpus, with serrate seta; carpus 4.7 L:W, 1x propodus, with two simple setae, rod seta 0.3x propodus, and blade-like spine 0.2x propodus; propodus 5.6 L:W, 2.8x dactylus and unguis combined length, with four serrate setae; dactylus and unguis fused with a small hook on tip.

Pleopods (Fig. 6E) exopod with four, endopod with 7 plumose setae.

Uropod (Fig. 6F) peduncle 0.9 L:W; exopod one articled, 6.7 L:W, with strong seta 0.5x endopod; endopod article-1 3.9 L:W, article-2 2.4 L:W, with four simple and one penicillate seta.

**Male description**. BL = 1.8 mm. Body robust (Fig. 7A,B), 3.9 L:W. Carapace 0.7 L:W, 4.8x pereonite-1, 0.2x BL. Pereonites 0.3x BL, pereonites 1–6: 0.2, 0.2, 0.3, 0.3, 0.3 and 0.3 L:W, respectively. Pleon short, 0.5x BL. Pleonites 0.4 L:W.

Antennule (Fig. 8A) 7-articled; article-1 0.3x total length, 1.9 L:W, 1.7x article-2, with one penicillate and nine simple setae (six broken); article-2 wide, 2.5x article-3, with two penicillate setae; article-3 0.7 L:W, 0.9x article-4, with three setae; article-4 1.2 L:W, 0.8x article-5; article-5 0.7 L:W, 0.2x article-6; article-6 4.5 L:W, 1.6 article-7; article 4–6 with dense row of aestetascs; article-7 5.7 L:W, with three setae.

Antenna (Fig. 8B) 7-articled; article-1 fused to body; article-2 0.8x article-3; article-3 0.3x article-4; article-4 0.5 article-5; article-5 1.4x article-6, with three penicillate setae in mid-length and with one penicillate and three simple setae; article-6 2.2x article-7, with two penicillate setae in mid-length and with two penicillate and one simple seta distally; article-7 with subdistal seta and four distal setae.

Maxilliped (Fig. 8C) basis 0.9 L:W, endites separated, distal margin naked; article-3 with three setae; article-4 with five setae.

Cheliped (Fig. 8D) slender, basis 1.6 L:W; merus with seta; carpus 1.7 L:W, with dorso-distal seta and two ventral setae; chela non-forcipate; palm 1.7 L:W, with row of eight short and one long setae on inner side; fixed finger distal spine pointed, regular size, with three ventral setae, and two dorsal setae, cutting edge serrate, dactylus 4.3 L:W, proximal seta present.

Pereopod-1 (Fig. 8E) basis 6.2 L:W, 2.8x merus, with two setae; ischium with ventral seta; merus 3.6 L:W, 0.7x carpus, with one seta; carpus 4.0 L:W, 0.8x propodus, with four setae; propodus 7.5 L:W, with two setae.

Pereopod-2 (Fig. 8F) basis 6.4 L:W, 3.0x merus, with three simple and one penicillate setae; ischium with ventral seta; merus 2.5 L:W, 0.7x carpus, with spine; carpus 4.2 L:W, 0.7x propodus, with two simple setae and one spine; propodus 7.0 L:W, with two setae and one spine.

Pereopod-3 (Fig. 8G) basis 6.4 L:W, 3.4x merus, with two simple and one penicillate setae; ischium with ventral seta; merus 2.2 L:W, 0.6x carpus, with seta and spine; carpus 4.0 L:W, with two setae and three spines.

Pereopod-4 (Fig. 8H) basis 3.7 L:W, 2.5x merus, with three setae; ischium with two setae; merus 2.8 L:W, 0.9x carpus, with spine; carpus 2.8 L:W, 1.4x dactylus and unguis combined length, with two spines; dactylus 1.8x unguis.

Pereopod-5 (Fig. 8I) basis 3.2 L:W, 2.7x merus, with two simple setae; ischium with two setae; merus 2.4 L:W, 0.9x carpus, with two distal spines; carpus 2.7 L:W, with seta and two spines.

Pereopod-6 (Fig. 8J) basis 3.3 L:W, 2.2x merus, with three simple setae; ischium with two ventral setae; merus 2.6 L:W and carpus, with one seta and three spines; carpus 7.7 L:W, 1.5x dactylus and unguis combined length, with three spines; dactylus 1.6x unguis.

Pleopods (Fig. 8K) exopod with eleven, endopod with 14 plumose setae.

Uropod (Fig. 8L) peduncle 1.3 L:W; exopod with two articles, 0.6x endopod, article-1 3.3 L:W, article-2 5.5 L:W, with simple seta; endopod article-1 4.2 L:W, with row of six penicillate mid-length setae and two penicillate distal setae; article-2 7.0 L:W, with three short and one long setae.

**Distribution:** *B*. *apocalyptica* n. sp. is known from three stations located in the licence area of the consortium Interoceanometal (IOM) at 4365 m depth and in the Belgium license area (GSR) at 4510 m depth in the Central Pacific.

**Remarks:** In the holotype specimen, a parasitic nematode was observed in the distal part of the body (Fig. 4).

**Genus**: *Pseudotanais* G.O. Sars, 1882

Diagnosis: Antenna article-6 and maxilliped palp article-4 without rod (thickened) seta. Chela cutting edges simple; fixed finger with one seta. Pereopod 2–6 carpus with blade-like spine.

*Pseudotanais* species described in the present study are grouped into previously erected morpho-groups by Bird and Holdich (1989)^31^ and Jakiel *et al*. (2018)^32^. A list of characters that define each group are included before the species descriptions. An identification key is included at the end of the Results section as well to enable easier identification and clear separation of morpho-groups.

$$ \mbox{'} {\rm{affinis}}+{\rm{longisetosus}}\mbox{'}\,{\rm{group}}$$

**Diagnosis:** Antenna article 2–3 with spines. Mandible acuminate or wide. Chela non-forcipate. Pereopod-1 merus with long seta. Pereopod-2 carpus with long blade-like spine. Uropod slender with exopod uropod about 3/4^th^ the endopod or equal to endopod.

Species included: *Pseudotanais affinis* Hansen, 1887; *P*. *longisetosus* Sieg, 1977; *P*. *longispinus* Bird & Holdich, 1989; *P*. *macrochelis* Sars, 1882; *P*. *nipponicus* McLelland, 2007; *P*. *nordenskioldi* Sieg, 1977; *P*. *spatula* Bird & Holdich, 1989; *P*. *scalpellum* Bird & Holdich, 1989; *P*. *svavarssoni* Jakiel, Stępień & Błażewicz, 2018; *P*. *vitjazi* Kudinova-Pasternak, 1966; *Pseudotanais* sp. O (McLelland, 2008); *Pseudotanais* sp. P (McLelland 2008); *P*. *gaiae* n. sp.; *P*. *geralti* n. sp.; *P*. *julietae* n. sp.; *P*. *romeo* n. sp.; *P*. *uranos* n. sp.; *P*. *yenneferae* n. sp.

***Pseudotanais uranos*** **n**. **sp**.

Figures 9–11.

**Figure 9:**
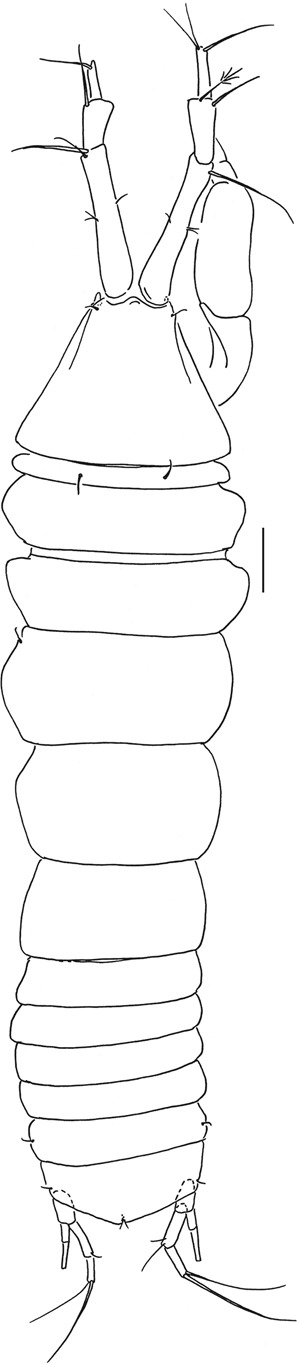
*Pseudotanais uranos* n. sp., ZMH K-56605, holotype neuter. Dorsal view. Scale bar: 0.1 mm.

**Figure 10:**
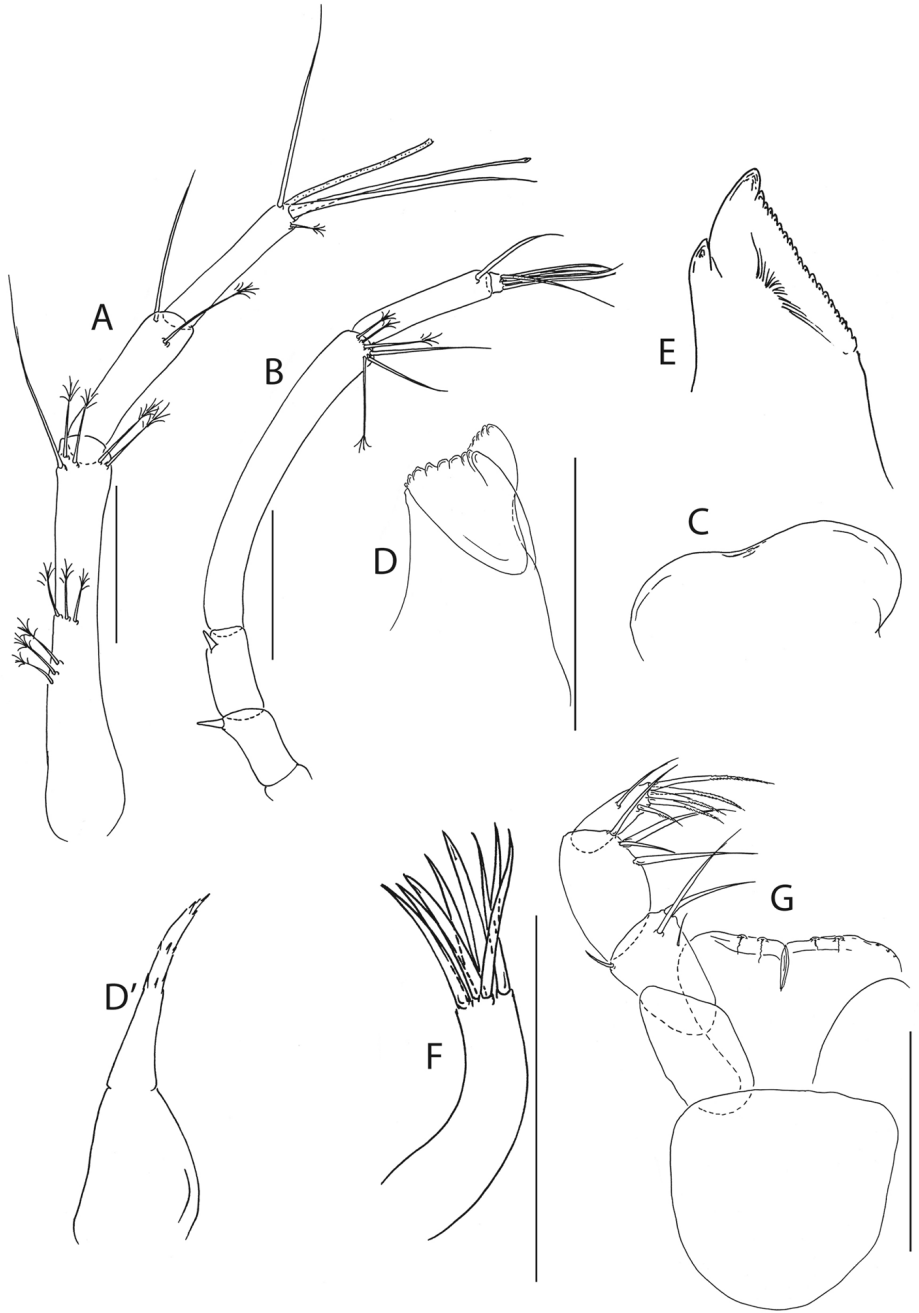
*Pseudotanais uranos* n. sp., ZMH K-56606, neuter. (**A**), antennule; (**B**), antenna; (**C**), labrum; (**D**), left mandible; D’ left molar; (**E**), right mandible; (**F**), maxillule; (**G**), maxilliped. Scale bar: 0.1 mm.

**Figure 11:**
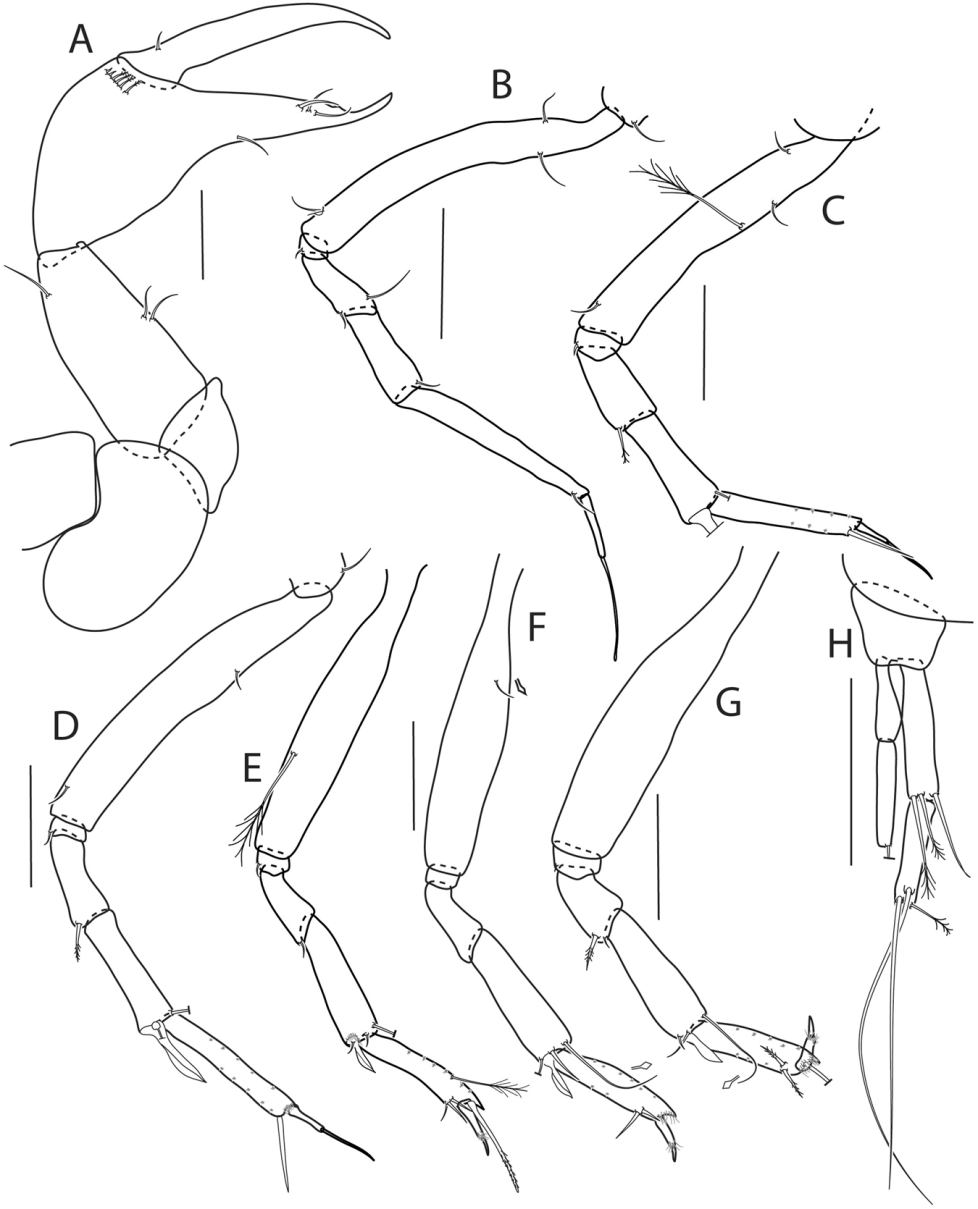
*Pseudotanais uranos* n. sp., ZMH K-56606, neuter (**A**), cheliped; (**B**), pereopod-1; (**C**), pereopod-2; (**D**), pereopod-3; (**E**), pereopod-4; (**F**), pereopod-5; (**G**), pereopod-6; (**H**), uropod. Insets at (**F**,**G**) show detail of tip of the rod seta. Scale bars: 0.1 mm.

**Material examined:** Holotype: neuter, BL = 1.5 mm, ZMH K-56606. St 197, 18° 48.66′N 128° 22.75′W, 4805 m, EBS, 22 Apr 2015.

**Paratypes:** four neuters, BL = 1.4–1.8 mm, ZMH K-56604 (dissected), ZMH K-56605, ZMH K-56607, ZMH K-56608. St 197, 18° 48.66′N 128° 22.75′W, 4805 m, EBS, 22 Apr 2015.

**Diagnosis:** Mandible molar acuminate without central spine. Pereopod-1 basis with three setae. Pereopod 5–6 carpus rod seta long (≥0.8x propodus).

**Etymology:** The name is dedicated to Uranos, the Greek god personifying the sky.

**Description of neuter**. BL = 1.5 mm. Body slender (Fig. 9), 4.0 L:W. Carapace 1.2 L:W, 6.8x pereonite-1, 0.2x BL. Pereonites 0.5x BL, pereonites-1–6: 0.2, 0.9, 0.4, 0.5, 0.6 and 0.5 L:W, respectively. Pleon short, 0.3x BL. Pleonites 0.9 L:W. Pleotelson 0.7x pereonite-6.

Antennule (Fig. 10A) article-1 0.5x total length, 6.8 L:W, 2.3x article-2, with six penicillate setae arranged in two rows at mid-length, and four penicillate and one simple setae; article-2 4.0 L:W, 1.1x article-3, with one penicillate and one simple setae; article-3 6.8 L:W, with one penicillate, one bifurcate and two simple setae, and with aestetasc distally.

Antenna (Fig. 10B) article-2 2.1 L:W; article-2 0.8x article-3, with spine 0.3x article-2; article-3 2.2 L:W, 0.3x article-4, with spine 0.2x the article-3; article-4 10.0 L:W, 2.4x article-5, with two simple and four penicillate setae distally; article-5 5.0 L:W, 10.0x article-6, with seta; article-6 0.7 L:W, with five setae.

Mouthparts. Labrum (Fig. 10C) hood-shape. Left mandible (Fig. 10D) *lacinia mobilis* well developed, distally serrate, incisor distal margin serrate, molar acuminate. Right mandible (Fig. 10E) incisor distal margin serrate, *lacina mobilis* merged to small process. Maxillule (Fig. 10F) with eight distal spines. Maxilliped (Fig. 10G) endites merged, with groove at mid-length, distal margin with two tubercles (gustatory cusps); palp article-2 inner margin with three setae, outer margin with seta; article-3 with four inner setae; article-4 with six distal and subdistal setae.

Cheliped (Fig. 11A) slender; basis 1.7 L:W, carpus 3.0 L:W, with two ventral and one dorsosubdistal setae; chela non-forcipate, palm 1.3 L:W, with row of six setae on inner side, fixed finger distal spine pointed, regular size, with three ventral setae; dactylus 6.5 L:W, ventral margin smooth, proximal seta present.

Pereopod-1 (Fig. 11B) coxa with seta; basis 9.3 L:W, with two ventral setae and one dorsal seta; ischium with ventral seta; merus 1.8 L:W, 1.5x carpus, with one short and one long setae; carpus 2.5 L:W, 0.5x propodus, with seta; propodus 10.2 L:W, with seta, 1.3x dactylus and unguis combined length; dactylus 0.6x unguis.

Pereopod-2 (Fig. 11C) basis 5.8 L:W, 3.4x merus, with two simple ventral setae, and with one simple and one penicillate setae dorsally; ischium with seta; merus 1.9 L:W, 0.8x carpus, with serrate seta; carpus 2.8 L:W, 0.8x propodus, with one seta and one blade-like spine (broken); propodus 7.0 L:W, 1.5x dactylus and unguis combined length, with distal seta and microtrichia on ventral margin; dactylus 0.6x unguis.

Pereopod-3 (Fig. 11D) coxa with seta; basis 6.7 L:W, 3.9x merus, with one ventral and one dorsal setae; ischium with ventral seta; merus 2.4 L:W, 0.7x carpus, with serrate seta; carpus 4.0 L:W, 0.8x propodus, with one simple and one wide-base seta and with blade-like spine 0.5x propodus; propodus 7.8 L:W, 1.5x dactylus and unguis combined length, with distal seta and microtrichiae on ventral margin; dactylus 0.7x unguis.

Pereopod-4 (Fig. 11E) basis 6.2 L:W, 4.1x merus, with penicillate ventral seta; ischium with seta; merus 2.5 L:W, 0.6x carpus, with seta; carpus 3.6 L:W, with two short and one rod setae, and with blade-like spine 0.3x propodus; propodus 5 L:W, 2.3x dactylus and unguis combined length, with one simple and two serrate setae subdistally, and with serrate seta distally 0.8x propodus and microtrichiae on ventral margin; dactylus 2.7x unguis.

Pereopod-5 (Fig. 11F) basis 5.6 L:W, 4.1x merus, with rod seta at mid-length; merus 3.0 L:W, 0.5x carpus; carpus 3.5 L:W, 1.3x propodus, with two simple and one rod setae 0.7x propodus, and with blade-like spine 0.4x propodus; propodus 4.5 L:W, 3.0x dactylus and unguis combined length, with two serrate subdistal setae, serrate distal seta (broken) and microtrichiae on ventral margin; dactylus 2.0x unguis.

Pereopod-6 (Fig. 11G) basis 5.5 L:W, 4.7x merus; ischium with ventral seta; merus 1.7 L:W, 0.5x carpus, with one simple and one serrate setae; carpus 3.0 L:W, 1.1x propodus, with two simple and one rod setae, and with blade-like spine 0.4x propodus, rod seta 0.8x propodus; propodus 4.0 L:W, 2.2x dactylus and unguis combined length, with two serrate setae subdistally, serrate distal seta broken and with microtrichiae on ventral margin; dactylus 1.7x unguis.

Uropod (Fig. 11H) peduncle 0.8 L:W; exopod with two articles, article-1 2.7 L:W; article-2 6.7 L:W with distal seta; endopod article-1 4.7 L:W, with one simple and two penicillate setae; article-2 5.5 L:W, with one penicillate and two simple setae. Exopod 0.7x endopod.

**Distribution:** *P*. *uranos* n. sp. is known only from APEI3 on the Clarion and Clipperton Fractures Zone, Central Pacific.

**Remarks:** Long rod seta on pereopods 5–6 of *P*. *uranos* n. sp. allows to distinguish this species from *Pseudotanais affinis*, *P*. *macrochelis*, *P*. *nordenskioldi*, *P*. *scalpellum*, *P*. *svavarssoni*, *P*. *vitjazi* and *Pseudotanais* sp. P (McLelland, 2008), which have short rod seta on pereopod 5–6 carpus. *P*. *uranos* has only three seta on basis of pereopod-1, whereas *P*. *longispinus* and *P*. *nipponicus* have more (5–7) setae. *P*. *uranos* n. sp. pereonite-1 is shorter than pereonite-2 whereas *P*. *longisetosus* has pereonite-1 as long as pereonite-2. Finally, *P*. *uranos* n. sp. has a semilong (0.5x propodus) blade-like spine on carpus of pereopod-3, while *P*. *spatula* and *Pseudotanais* sp. O^33^ have a long (≥=0.6x propodus) blade-like spine on carpus of pereopod-3.

***Pseudotanais gaiae*** **n**. **sp**.

Figure 12 and 13.

**Figure 12:**
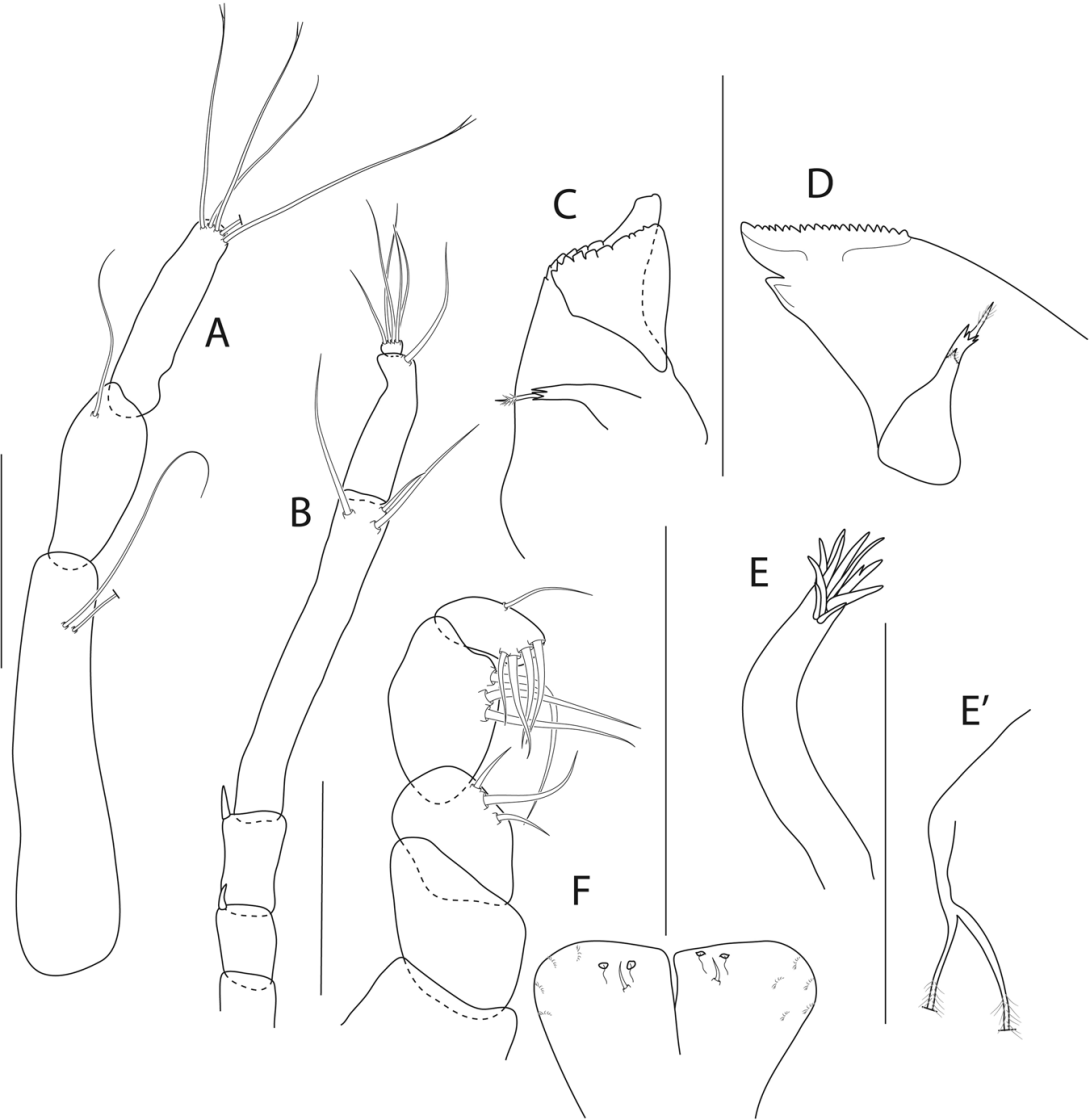
*Pseudotanais gaiae* n. sp., ZMH K-56576, holotype neuter. (**A**), antennule; (**B**), antenna; (**C**), left mandible; (**D**), right mandible; (**E**), maxillule; E’ endit; (**F**), maxilliped. Scale bar: 0.1 mm.

**Figure 13:**
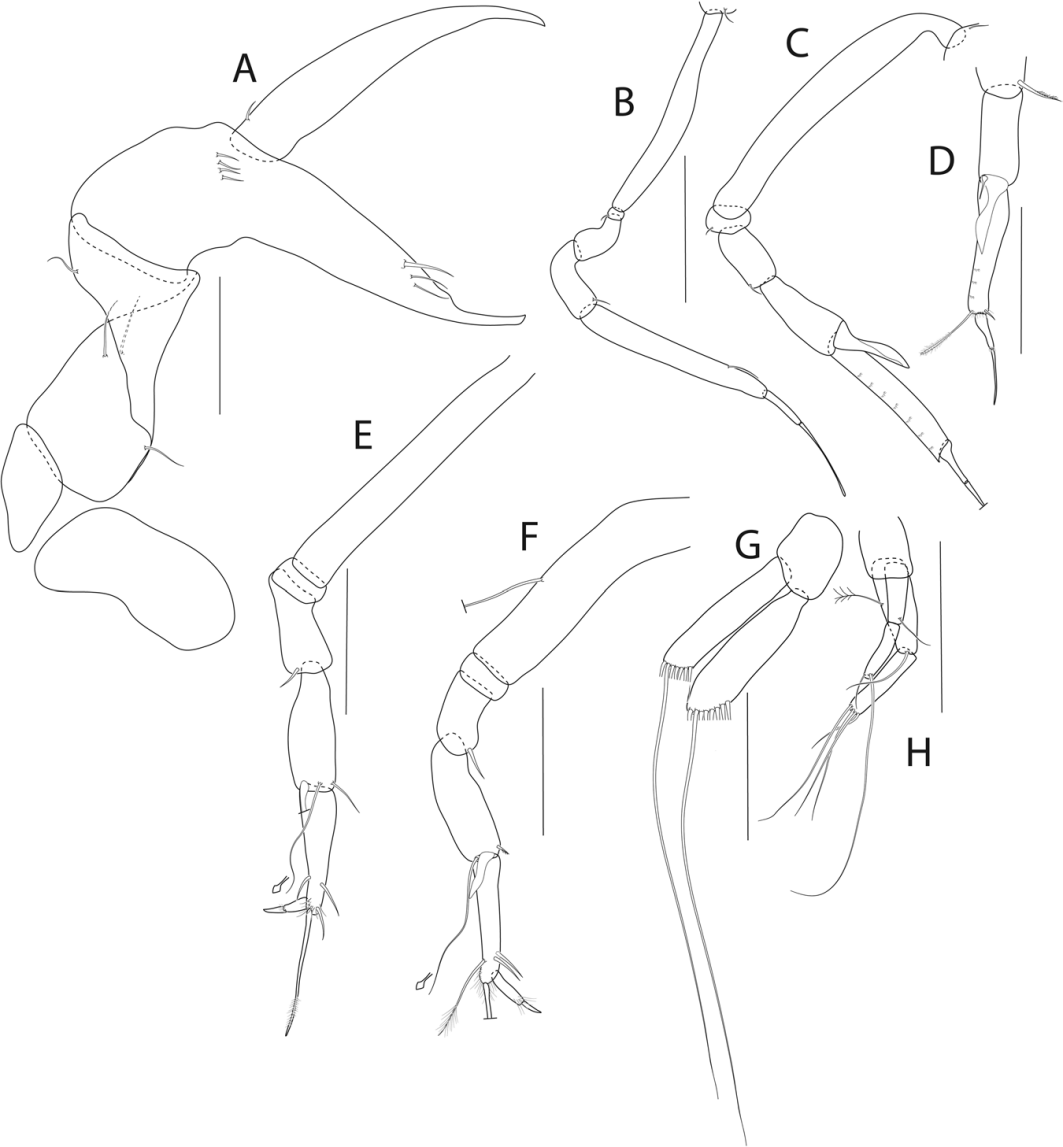
*Pseudotanais gaiae* n. sp., ZMH K-56576, holotype neuter. (**A**), cheliped; (**B**), pereopod-1; (**C**), pereopod-2; (**D**), pereopod-3; (**E**), pereopod-5; (**F**), pereopod-6; (**G**), pleopod; (**H**), uropod. Insets at (**E**,**F**) show detail of tip of the rod seta. Scale bar: 0.1 mm.

Material examined: Holotype: neuter (dissected), BL = 1.5 mm, ZMH K-56576. St 192, 18° 44.81′N 128° 21.87′W, 4877 m, EBS, 21 Apr 2015.

**Diagnosis:** Mandible molar acuminate with central, elongated spine. Pereopod-1 basis without setae. Pereopod 5–6 carpus rod seta long.

**Etymology:** The species is named after Gaia, the ancestral mother of all life – Mother Earth; the wife of Uranos.

**Description**. Antennule (Fig. 12A) article-1 0.5x total length, 5.0 L:W, 2.3x article-2, with two setae; article-2 0.4 L:W, 0.8x article-3; article-3 4.2 L:W, with one simple, three bifurcate and one broken setae distally.

Antenna (Fig. 12B) article-2 1.5 L:W, 0.8x article-3, with spine 0.3x the article-2; article-3 1.8 L:W, 0.3x article-4, with spine 0.3x the article-3; article-4 7.5 L:W, 2.2x article-5, with three simple setae; article-5 9.6 L:W, 9.0x article-6, with distal seta; article-6 0.7 L:W, with five setae.

Mouthparts. Left mandible (Fig. 12C) *lacinia mobilis* well developed and serrate distally, incisor distal margin serrate, molar pointed, with central, elongated spine. Right mandible (Fig. 12D) incisor distal margin serrate, *lacina mobilis* merged to small process; molar as in mandible left. Maxillule (Fig. 12E,E’) with eight simple and one bifurcate distal spines. Maxilliped (Fig. 12F) endites merged, with groove in the mid-length, distal margin with two tubercles (gustatory cusps) and seta; article-2 inner margin with three inner setae; article-3 with three setae, article-4 with five setae.

Cheliped (Fig. 13A) slender; basis 2.0 L:W; carpus 1.8 L:W, with two ventral setae and subdistal dorsal seta; palm 1.1 L:W, with row of four setae on inner side; fixed finger distal spine pointed, regular size, 2.3x palm, with three ventral setae; dactylus 6.0 L:W, proximal seta present.

Pereopod-1 (Fig. 13B) coxa with seta; basis 8.0 L:W; ischium with ventral seta; merus 2.2 L:W, 0.8x carpus; carpus 2.8 L:W with seta, 0.4x propodus; propodus 7.2 L:W, 2.4x dactylus and unguis combined length, with seta; dactylus 0.6x unguis.

Pereopod-2 (Fig. 13C) coxa with seta; basis 8.6 L:W, 10.0x merus; ischium with ventral seta; merus 2.0 L:W, 0.7x carpus, with seta; carpus 2.4 L:W, 0.6x propodus, with blade-like spine 0.6x propodus; propodus 5.8 L:W, with microtrichia.

Pereopod-3 (Fig. 13D) basis, ischium and merus broken (not seen); merus with serrate seta; carpus 2.6 L:W, 0.7x propodus, with wide-base seta and one blade-like spine, 0.5x propodus; propodus 5.2 L:W, 1.5x dactylus and unguis combined length, with one simple and one serrate seta and microtrichia on ventral margin; dactylus 0.7x unguis.

Pereopod-5 (Fig. 13E) basis 7.8 L:W, 3.2x merus; merus 2.1 L:W, 0.7x carpus, with serrate seta; carpus three L:W, 1.1x propodus, with one simple, one rod setae, and one blade-like spine (broken), rod seta propodus; propodus 3.7 L:W, 2.4x dactylus and unguis combined length, two serrate setae subdistally, one simple and one serrate setae distally 1x propodus; dactylus 1.2x unguis.

Pereopod-6 (Fig. 13F) basis 5.0 L:W, 3.5x merus, merus 2.0 L:W, 0.6x carpus, with seta; carpus 3.4 L:W, propodus, with serrate seta, rod seta propodus and blade-like spine 0.4x propodus; propodus 6.0 L:W, 2.0x dactylus and unguis combined length, with one penicillate and two serrate setae subdistally, and serrate seta distally; dactylus 2.0x unguis.

Pleopods (Fig. 13G) exopod with seven and endopod with eight plumose setae.

Uropod (Fig. 13H) 1.4 L:W, exopod with two articles, 0.7x endopod; article-1 2.7 L:W, with seta; article-2 3.6 L:W, with two setae; endopod article-1 3.0 L:W, with one mid-length penicillate and one distal setae; article-2 3.7 L:W, with four simple setae.

**Distribution:** *P*. *gaiae* n. sp. is known only from APEI3 of the Clarion and Clipperton Fractures Zone, Central Pacific.

**Remarks**: *Pseudotanais gaiae* n. sp. is most similar to *P*. *uranos* (Fig. 1) and therefore is distinguished from *Pseudotanais affinis*, *P*. *macrochelis*, *P*. *nordenskioldi*, *P*. *scalpellum*, *P*. *svavarssoni*, *P*. *vitjazi*, *Pseudotanais* sp. P (McLelland, 2008), *P*. *longispinus* and *P*. *nipponicus* by the same set of characters as *P*. *uranos* (see remarks under *P*. *uranos*). *P*.*gaiae* n. sp., with two prickly tubercles (gustatory cusps) and a seta in the maxilliped endites, is distinguished from *P*. *longisetosus,* which maxilliped endite is naked. *P*. *gaiae* n. sp. with short seta (0.2x carpus) on pereopod-1 carpus is separated from *P*. *spatula* that has pereopod-1 carpus with seta long (0.9x carpus). *P*. *gaiae* and *P*. *uranos* represent cryptic species, with minute morphological differences, that can be separated using molecular data. The main morphological character that allows distinguishing *P*. *gaiae* from *P*. *uranos* is the presence of a central elongated spine on the mandible molar.

***Pseudotanais julietae*** **n**. **sp**.

Figures 14–16.

**Figure 14:**
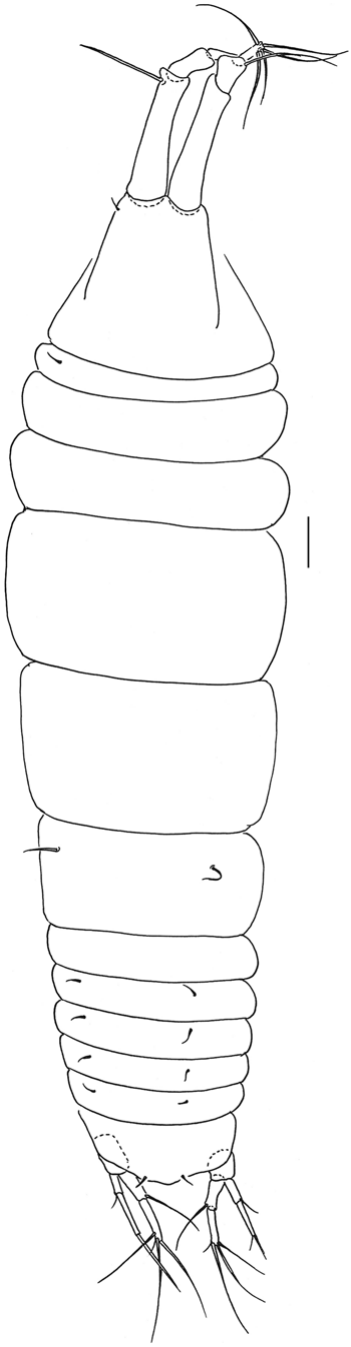
*Pseudotanais julietae* n. sp., ZMH K-56584, holotype neuter. Dorsal view. Scale bar: 1 mm.

**Figure 15:**
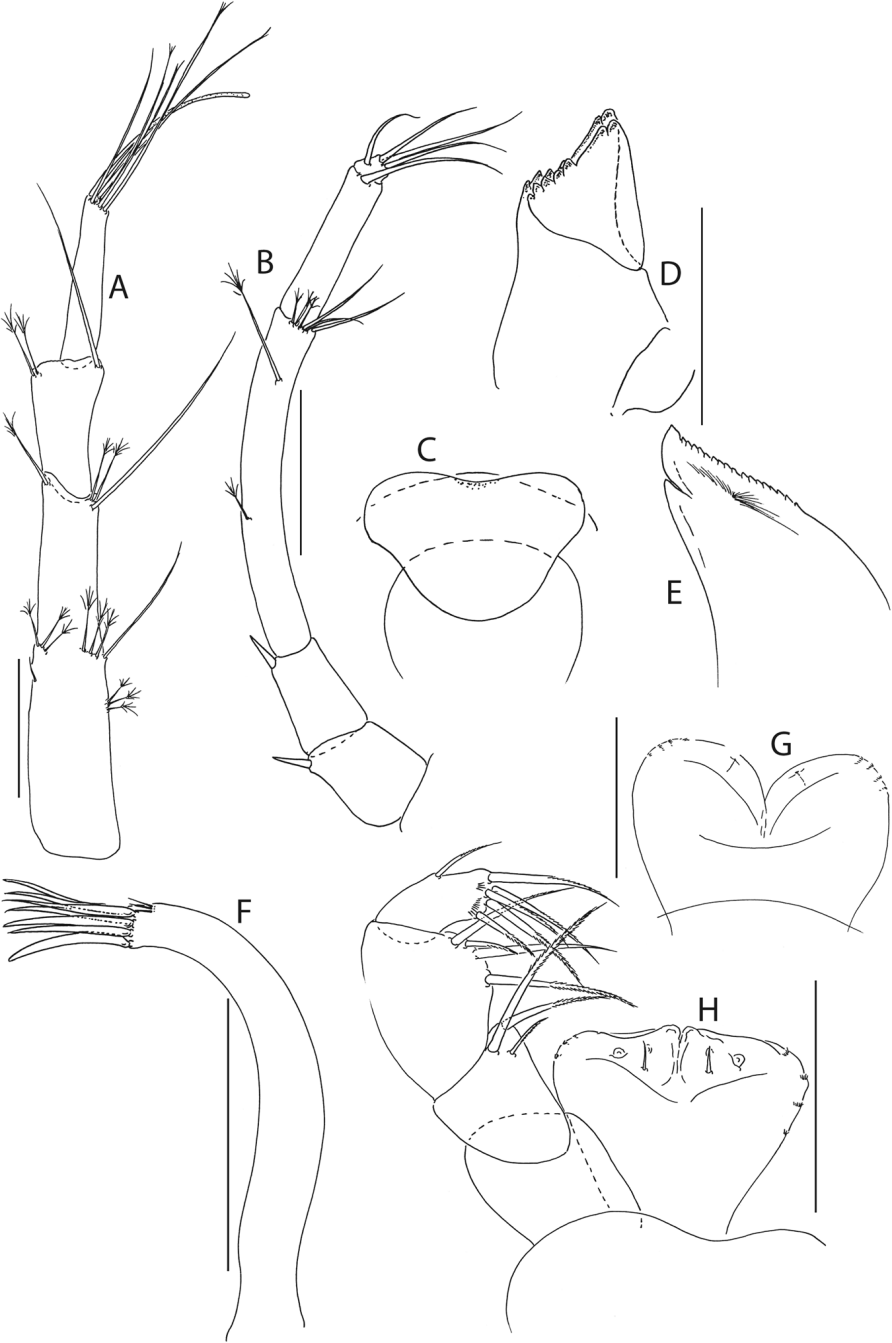
*Pseudotanais julietae* n. sp., ZMH K-56584, holotype neuter. (**A)**, antennule; (**B)**, antenna; (**C)**, labrum; (**D)**, left mandible; (**E)**, right mandible; (**F)**, maxillule; (**G)**, labium; (**H)**, maxilliped. Scale bar: 0.1 mm.

**Figure 16:**
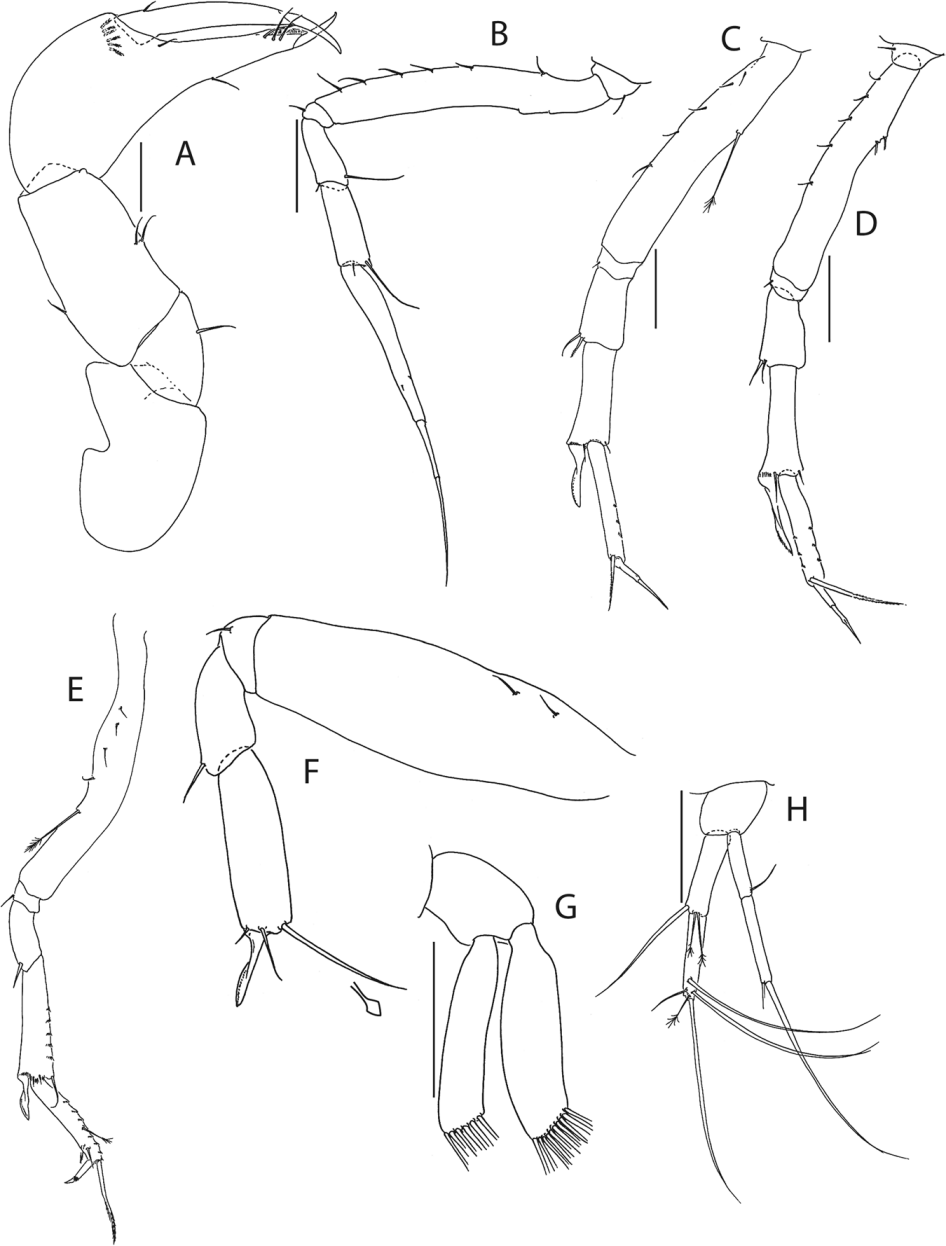
*Pseudotanais julietae* n. sp., ZMH K-56584, holotype neuter. (**A)**, cheliped; (**B)**, pereopod-1; (**C)**, pereopod-2; (**D)**, pereopod-3; (**E)**, pereopod-4; (**F)**, pereopod-6; (**G)**, pleopod; (**H)**, uropod. Inset at (**F)** show detail of tip of the rod seta. Scale bar: 0.1 mm.

**Material examined:** Holotype: neuter, BL = 1.8 mm (partly dissected), ZMH K-56584. St 133, 13° 50.98′N 1 23° 15.07′W, 4507 m, 10 Apr 2015.

**Diagnosis:** Maxilliped endites ornamented with two tubercles (gustatory cusps) and one seta. Pereopods 1–3 basis with six, five and five setae respectively. Pereopod 5–6 carpus with long distodorsal rod seta. Exopod of the uropod as long as endopod.

**Etymology:** The species is named after Juliet Capulet, the lover of Romeo from William Shakespeare’s tragedy *Romeo and Juliet*.

**Description of neuter**. BL = 1.8 mm. Body robust (Fig. 14), 3.4 L:W. Carapace 0.8 L:W, 8.0x pereonite-1, 0.2x BL. Pereonites 0.6x BL, pereonites-1–6: 0.1, 0.2, 0. 2, 0. 6, 0.6 and 0.5 L:W, respectively. Pleon short, 0.2x BL. Pleonites 0.9 L:W.

Antennule (Fig. 15A) article-1 0.6x total length, 4.5 L:W, 3.1x article-2, with one simple and nine penicillate mid-length setae, and with one simple and three penicillate distal setae; article-2 1.9 L:W, 0.8x article-3, with one simple and two penicillate setae distally; article-3 4.6 L:W, with one simple, four bifurcate setae, and one aestetasc.

Antenna (Fig. 15B) 1.3 L:W; article-1 not observed; article-2 1.2x article-3, with spine 0.4x the article-2; article-3 1.4 L:W, 0.3x article-4, with spine, 0.4x the article-3; article-4 7.8 L:W, 2.1x article-5, with penicillate mid-length seta, one penicillate subdistal seta, three simple and three penicillate distal setae; article-5 4.1 L:W, 6.6x article-6, with distal seta; article-6 0.7 L:W, with four simple setae.

Mouthparts. Labrum (Fig. 15C) hood-shape, setose. Left mandible (Fig. 15D) *lacinia mobilis* well developed and distally serrate, incisor distal margin serrate. Right mandible (Fig. 15E) incisor distal margin serrate, *lacina mobilis* merged to small process, molar lost during dissection. Maxillule (Fig. 15F) with 7 distal spines and three subdistal setae. Labium (Fig. 15G) lobes with distolateral corner weakly setose. Maxilliped (Fig. 15H) endites merged, with groove in mid-length, distal margin with two tubercles (gustatory cusps) and seta; palp with article-2 three inner serrate setae; article-3 with three setae; article-4 with six setae.

Cheliped (Fig. 16A) slender; basis 1.8 L:W; merus with simple seta; carpus 1.8 L:W, with two ventral setae and dorsal seta; chela non-forcipate; palm 1.6 L:W, with row of six setae on inner side; fixed finger distal spine pointed, regular size, with three ventral setae; dactylus 9.2 L:W, with proximal seta.

Pereopod-1 (Fig. 16B) coxa with seta; basis 6.9 L:W, with six ventral setae and with two dorsal setae (broken); ischium with ventral seta; merus 2.8 L:W, 0.8x carpus, with two setae; carpus 2.8 L:W, 0.5x propodus, with two (long and short) setae; propodus 7.0 L:W, 1.1x dactylus and unguis combined length, with two setae; dactylus 0.5x unguis.

Pereopod-2 (Fig. 16C) basis 5.8 L:W, 3.7x merus with five ventral setae and dorsal penicillate seta; ischium with ventral seta; merus 1.6 L:W, 0.7x carpus, with two setae; carpus 2.7 L:W, 0.8x propodus, with two simple setae and blade-like spine 0.6x propodus; propodus 8.0 L:W, 1.7x dactylus and unguis combined length, with serrate distal seta and microtrichia on ventral margin; dactylus 0.7x unguis.

Pereopod-3 (Fig. 16D) coxa with seta; basis 5.6 L:W, 3.3x merus, with five ventral setae and two dorsal setae (broken); ischium with ventral seta; merus 1.7 L:W, 0.7x carpus, with two setae; carpus 3.5 L:W, 0.9x propodus, with two simple setae and with one blade-like spine 0.7x propodus; propodus 8.0 L:W, 1.6x dactylus and unguis combined length, with serrate distal seta and microtrichia on ventral margin; dactylus 0.7x unguis.

Pereopod-4 (Fig. 16E) basis 7.1 L:W, 4.1x merus, with four simple and one penicillate setae ventrally; ischium with ventral seta; merus 2.8 L:W, 0.6x carpus, with seta; carpus 4.6 L:W, 1.1x propodus, with two simple setae, one rod setae 0.4x propodus and one blade-like spine 0.4x propodus; propodus 5.0 L:W, 2.9x dactylus and unguis combined length, with one simple, one serrate and one penicillate setae subdistally, one serrate distal seta 0.7x propodus, and microtrichia on ventral margin; dactylus 2.5x unguis.

Pereopod-6 (Fig. 16F) basis 3.1 L:W, 3.4x merus, with two ventral setae; ischium with ventral seta; merus 2.0 L:W, 0.6x carpus, with seta; carpus 3.3 L:W, with two simple and one rod setae, and one blade-like spine.

Pleopods (Fig. 16G) exopod with six and endopod with 10 plumose setae.

Uropod (Fig. 16H) peduncle 0.9 L:W, exopod 0.9x endopod with two articles; article-1 4.3 L:W, with seta; article-2 8.5 L:W, with two setae; endopod article-1 4.2 L:W, with one simple, one penicillate setae distally; article-2 5.7 L:W, with four simple and one penicillate setae.

**Distribution:** *P*. *julietae* n. sp. is known from the Belgium licence area (GSR) of the Central Pacific.

**Remarks:** *P*. *julietae* can be distinguished from all other species of ‘affinis + longisetosus’ group because the exopod in uropods is always shorter than in all other members of the group (*Pseudotanais affinis*; *P*. *macrochelis*; *P*. *nordenskioldi*; *P*. *scalpellum*; *P*. *svavarssoni*; *P*. *vitjazi*; *Pseudotanais* sp. P; *P*. *longisetosus*; *P*. *longispinus*; *P*. *nipponicus*; *P*. *spatula*).

***Pseudotanais romeo*** **n**. **sp**.

Figures 17–19.

**Figure 17:**
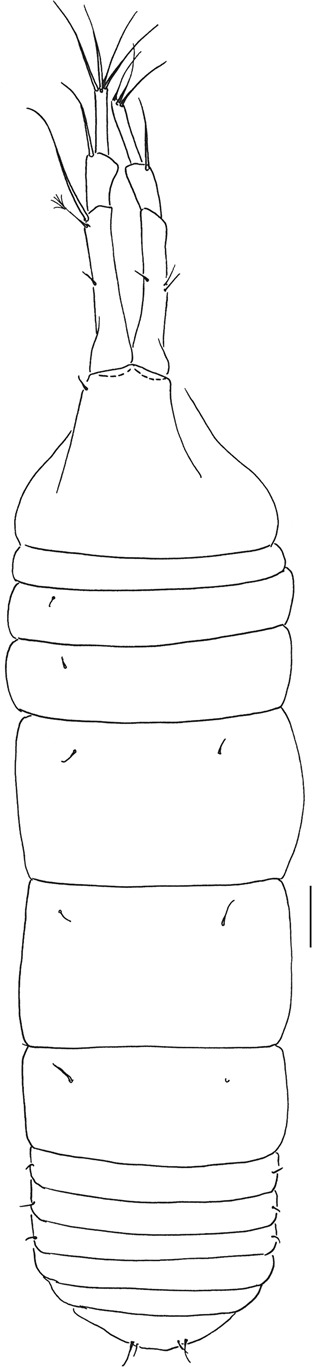
*Pseudotanais romeo* n. sp., ZMH K-56601, holotype neuter. Dorsal view. Scale bar: 1 mm.

**Figure 18:**
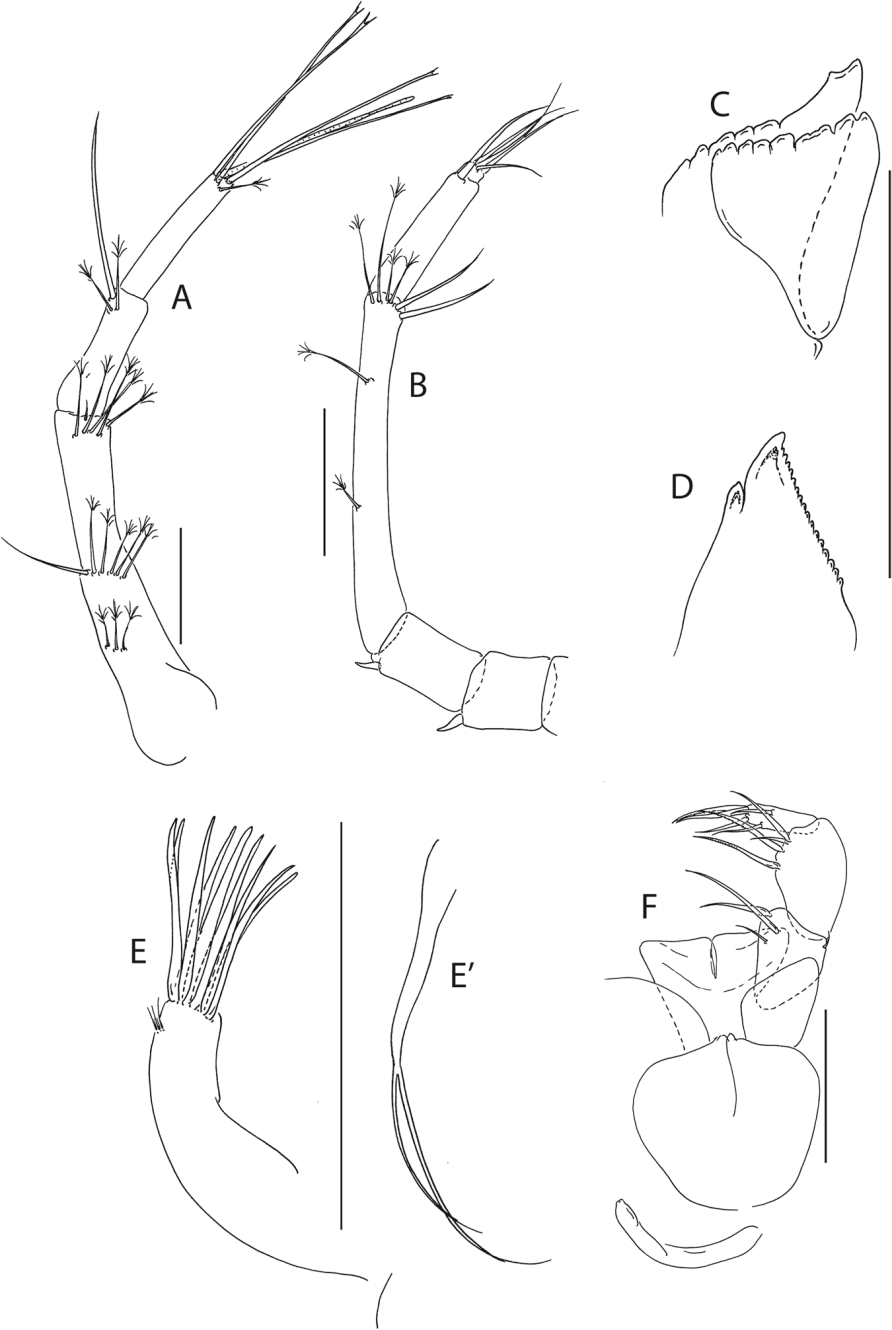
*Pseudotanais romeo* n. sp., ZMH K-56600, neuter. (**A)**, antennule; (**B)**, antenna; (**C)**, left mandible; (**D)**, right mandible; (**E)**, maxillule; E’ endit. Scale bar: 0.1 mm.

**Figure 19:**
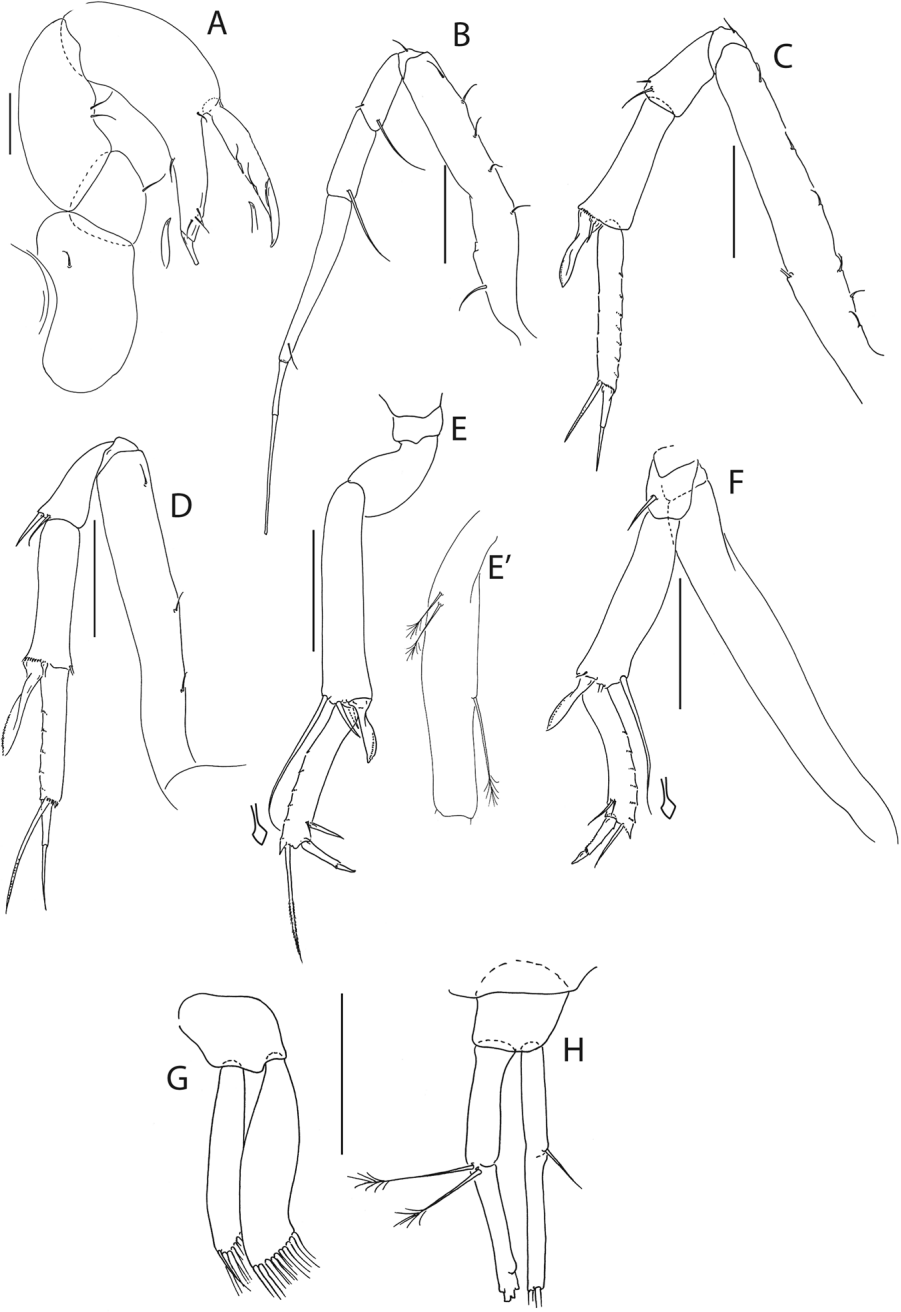
*Pseudotanais romeo* n. sp., ZMH K-56600, neuter. (**A)**, cheliped; (**B)**, pereopod-1; (**C**), pereopod-2; (**D)**, pereopod-3; (**E)**, pereopod-4; E’, basis of pereopod-4; (**F)**, pereopod-5; (**G)**, pleopod; (**H)**, uropod. Insets at (**E**,**F**) show detail of tip of the rod seta. Scale bar: 0.1 mm.

**Material examined:** Holotype: neuter, 1.7 mm, ZMH K-56601. St 24, 11° 51.52′N 117° 1.19′W, 4100 m, 22 Mar 2015.

Paratypes: neuter, BL = 1.6 mm, ZMH K-56599. St 20, 11° 49.81′N 117° 0.28′W, 4093 m, 22 Mar 2015; three neuters, BL = 1.4–1.8 mm (one dissected), ZMH K-56600 (dissected), ZMH K-56602, ZMH K-56603. St 24, 11° 51.52′N 117° 1.19′W, 4100 m, 22 Mar 2015.

**Diagnosis:** Maxilliped endite naked. Cheliped cutting edge on dactylus with two spines. Pereopods 1–3 basis with five, six and three setae respectively. Pereopod 5–6 carpus with long distodorsal rod seta. Exopod of the uropod as long as endopod.

**Etymology:** The species is named after Romeo Montague, the lover of Juliet from William Shakespeare’s tragedy *Romeo and Juliet*.

**Description of neuter**. BL = 1.7 mm. Body robust (Fig. 17), 3.3 L:W. Carapace 0.6 L:W, 6.2x pereonite-1, 0.2x BL. Pereonites 0.6x BL, pereonites-1–6: 0.1, 0.2, 0.3, 0.6, 0.6 and 0.4 L:W, respectively. Pleon short, 0.1x BL. Pleonites 0.6 L:W.

Antennule (Fig. 18A) article-1 0.6x total length, 7.0 L:W, 2.7x article-2, with one simple, eight penicillate mid-length setae and five penicillate distal setae; article-2 3.2 L:W, 0.9x article-3, with one simple and two penicillate distal setae; article-3 5.8 L:W, with one penicillate and four bifurcate setae, and one aestetasc.

Antenna (Fig. 18B) article-2 1.1 L:W, 0.7x article-3, with spine 0.4x the article-2; article-3 1.7 L:W, 0.3x article-4, with spine 0.2x the article-3; article-4 7.8 L:W, 2.5x article-5, with two penicillate mid-length setae, and two simple and four penicillate setae distally; article-5 4.0 L:W, 9.3x article-6, with distal seta; article-6 0.6 L:W, with four simple setae.

Mouthparts. Left mandible (Fig. 18C) *lacinia mobilis* well developed, distally serrate, incisor distal margin serrate. Right mandible (Fig. 18D) incisor distal margin serrate, *lacina mobilis* merged to a small process. Maxillule (Fig. 18E) with nine distal spines, endit with two distal setae (Fig. 18E’). Maxilliped (Fig. 18F) basis with groove 0.9 L:W, endites merged, with a groove in mid-length, naked; palp article-2 inner margin with three inner setae, outer margin with seta; article-3 with three setae; article-4 with five setae. Epignath (Fig. 18G) distally rounded.

Cheliped (Fig. 19A) robust; basis 1.6 L:W, with distoproximal seta; merus with seta; carpus 2.3 L:W, with two ventral setae; chela non-forcipate; palm 2.0 L:W; fixed finger distal spine pointed, regular size, with three ventral setae; dactylus 6.4 L:W, cutting edge with two spines.

Pereopod-1 (Fig. 19B) basis 7.5 L:W, with ventral seta and five dorsal setae; ischium with ventral seta; merus 3.0 L:W, 9.0x carpus, with two (long and short) setae; carpus 2.8 L:W, 0.5x propodus, with long seta; propodus 7.0 L:W, dactylus and unguis combined length, with seta; dactylus 0.1x unguis.

Pereopod-2 (Fig. 19C) basis 6.4 L:W, 4.7x merus, with six ventral setae and one dorsal seta; ischium with ventral seta; merus 1.6 L:W, 0.5x carpus, with two setae; carpus 2.7 L:W, 0.8x propodus, with seta and blade-like spine, 0.5x propodus; propodus 6.4 L:W, 1.8x dactylus and unguis combined length, with serrate seta and microtrichia on ventral margin; dactylus as long as unguis.

Pereopod-3 (Fig. 19D) basis 5.9 L:W, 3.6x merus, with three ventral setae; ischium naked; merus 2.0 L:W, 0.6x carpus, with two setae; carpus 3.6 L:W, 1.1x propodus, with one seta (broken), one spine (broken) and one blade-like spine 0.8x propodus; propodus 5.4 L:W, 2.2x dactylus and unguis combined length, with seta and microtrichia on ventral margin; dactylus 0.7x unguis.

Pereopod-4 (Fig. 19E,E’) basis 5.6 L:W, 3.3x merus, with penicillate ventral seta and two penicillate dorsal setae; ischium naked, merus 1.7 L:W, 0.4x carpus; carpus 5.6 L:W, 1.5x propodus, with rod setae as long as propodus, two spines and with blade-like spine 0.4x propodus; propodus 6 L:W, 2.5x dactylus and unguis combined length, with two ventral setae, one serrate dorsal seta 0.8x propodus and microtrichia on ventral margin; dactylus 2.0x unguis.

Pereopod-5 (Fig. 19F) basis 7.3 L:W, 7.3x merus; ischium naked; merus 1.1 L:W, 0.3x carpus, with seta; carpus 3.5 L:W, 1.2x propodus, with two simple setae, one rod seta 0.9x propodus, and with blade-like spine 0.5x propodus; propodus 6.0 L:W, 2.5x dactylus and unguis combined length, with two simple setae on ventral margin, one seta on dorsal margin, and microtrichia on ventral margin; dactylus 2.0x unguis.

Pleopods (Fig. 19G) exopod with seven and endopod with 10 plumose setae.

Uropod (Fig. 19H) peduncle 1.0 L:W; exopod 0.9x endopod, with two articles; article-1 4.6 L:W, with seta; article-2 6.2 L:W, with two setae; endopod article-1 4.3 L:W, with two penicillate distal setae; article-2 7.0 L:W, with five distal setae (broken).

**Distribution:** *P*. *romeo* n. sp. is known from the Belgium licence area (GSR) of the Central Pacific.

**Remarks:** *Pseudotanais romeo* n. sp. is morphologically and genetically most similar to *P*. *julietae* (Fig. 1) and it is distinguished from all other members of the ‘affinis + longisetosus’ group by the same character set as *P*. *julietae* (see remarks under *P*. *julietae*). *P*. *romeo* is distinguished from *P*. *julietae* by the number of setae on basis of pereopod 1–3: 5, 6, 3 and 6, 5, 5, respectively. *P*. *romeo* has naked maxillped endites whereas *P*. *julietae* has maxilliped endites ornamented with two tubercles (gustatory cusps) and one seta. The presence of two spines on cutting edge of the cheliped in *P*. *romeo* also allow to separate it from *P*. *julietae* with smooth cutting edge.

***Pseudotanais yenneferae*** **n**. **sp**.

Figures 20–22.

**Figure 20:**
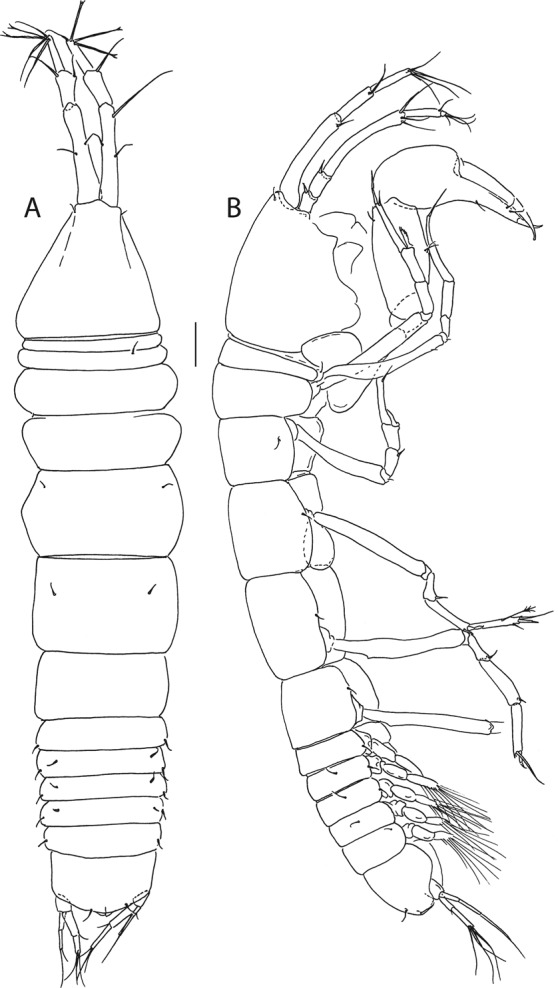
*Pseudotanais yenneferae* n. sp., ZMH K-56609, holotype female. A, dorsal view; B lateral view. Scale bar: 1 mm.

**Figure 21:**
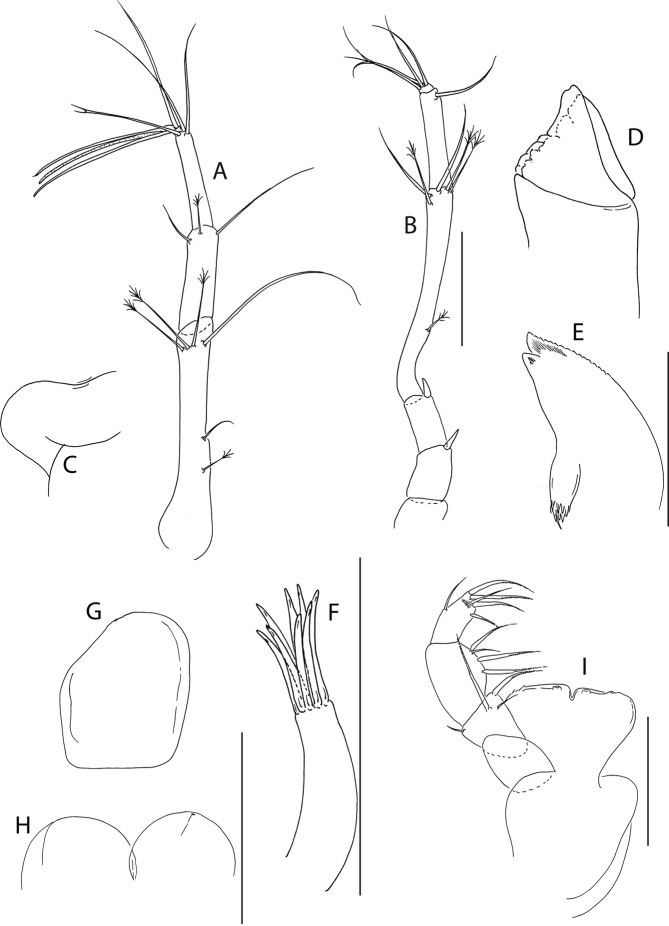
*Pseudotanais yenneferae* n. sp., ZMH K-56616, neuter. (**A**), antennule; (**B**), antenna; (**C**), labium; (**D**), left mandible; (**E**), right mandible; (**F**), maxillule; (**G**), maxilla; (**H**), labium; (**I**), maxilliped: (**J**), epignath. Scale bar: 0.1 mm.

**Figure 22:**
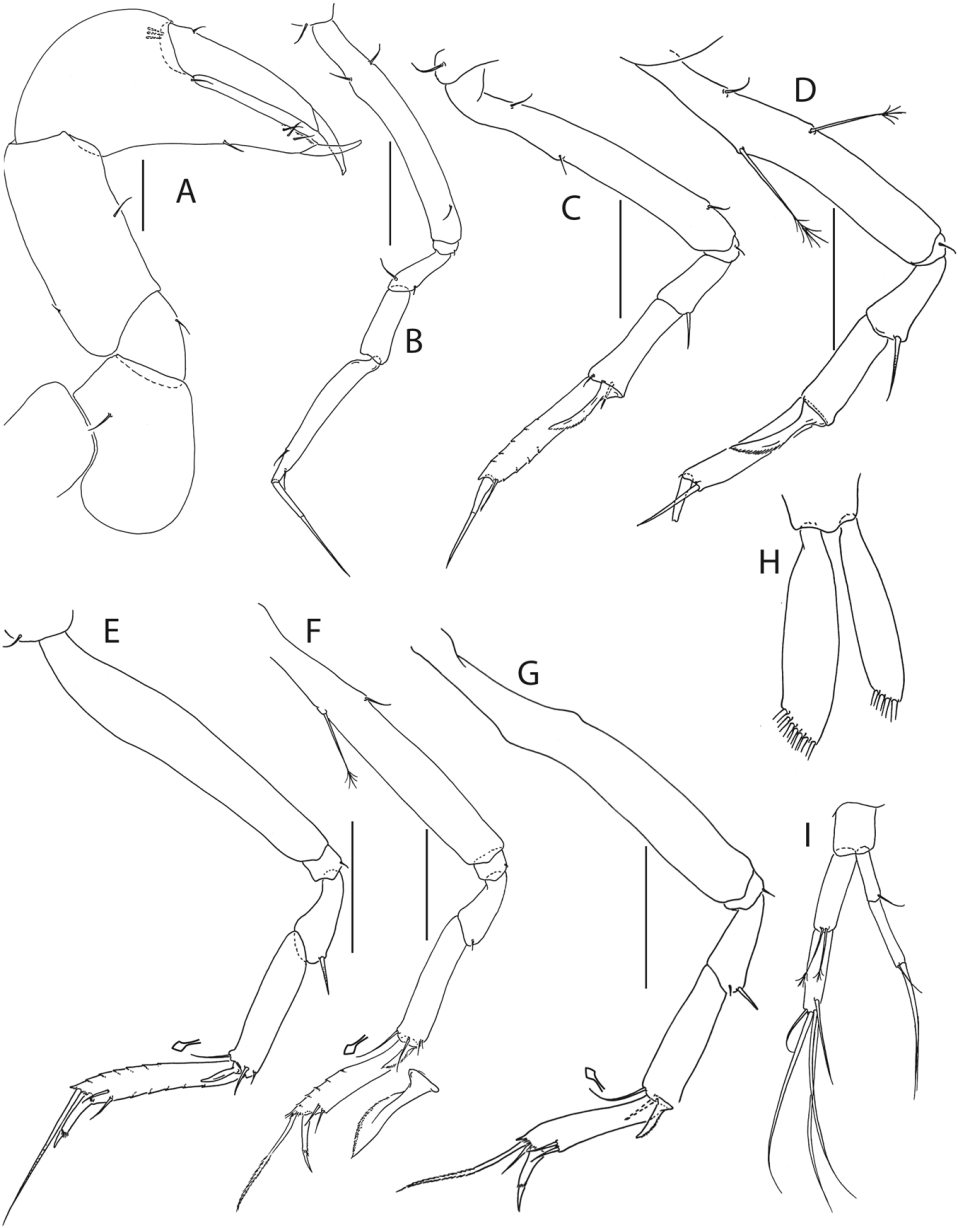
*Pseudotanais yenneferae* n. sp., ZMH K-56616, neuter. (**A**), cheliped; (**B**), pereopod-1; (**C**), pereopod-2; (**D**), pereopod-3; (**E**), pereopod-4; (**F**), pereopod-5; (**G**), pereopod-6; (**H**), pleopod; (**I**), uropod. Insets at (**E**–**G**) show detail of tip of the rod seta; on (**F**) a magnification of the blade-like spine. Scale bar: 0.1 mm.

**Material examined:** Holotype: female, BL = 1.5 mm, ZMH K-56609. St. 197, 18° 48.66′N 128° 22.75′W, 4805 m, 21 Apr 2015.

Paratype: neuter, BL = 1.1 mm, ZMH K-56618. St. 192, 18° 44.81′N 128° 21.87′W, 4877 m, 21 Apr 2015; eight neuters BL = 1.3–1.9 mm (one dissected), ZMH K-56610, ZMH K-56611, ZMH K-56612, ZMH K-56613, ZMH K-56614, ZMH K-56615, ZMH K-56616 (disstected), ZMH K-56617. St.197, 18° 48.66′N 128° 22.75′W, 4805 m, 22 Apr 2015.

**Diagnosis:** Mandible molar wide. Pereopod-1 basis with two setae. Pereopod 5–6 carpus with short distodorsal rod seta.

**Etymology:** The species is named after the female protagonist partner of Polish fantasy novel ‘Wiedźmin’ (eng. The Witcher) written by Andrzej Sapkowski.

**Description of neuter**. BL = 1.5 mm. Body slender (Fig. 20A,B), 4.4 L:W. Carapace 0.9 L:W, 7.2x pereonite-1, 0.2x BL. Pereonites 0.5x BL pereonites-1–6: 0.1, 0.3, 0.3, 0.5, 0.6 and 0.5 L:W, respectively. Pleon short, 0.2x BL. Pleonites 1.1 L:W.

Antennule (Fig. 21A) article-1 0.5x total length, 7.6 L:W, 2.3x article-2, with one simple, one penicillate seta at mid-length, and one simple, three penicillate setae distally; article-2 3.6 L:W, 1.1x article-3, with two simple and one penicillate setae distally; article-3 5.4 L:W, with three simple, three bifurcate setae and one aestetasc.

Antenna (Fig. 21B) article-2 1.4 L:W; article-3, with spine 0.4x the article-2; article-3 1.9 L:W, 0.2x article-4, with spine 0.3x the article-3; article-4 10.0 L:W, 2.2x article-5, with penicillate mid-length seta and two simple, and three penicillate setae distally; article-5 5.4 L:W, 13.5x article-6, with distal seta; article-6 0.5 L:W, with five setae.

Mouthparts. Labrum (Fig. C) naked. Left mandible (Fig. 21D) *lacinia mobilis* well developed and distally serrate, incisor distal margin serrate. Right mandible (Fig. 21E) incisor distal margin serrate, *lacina mobilis* merged to a small process. Maxillule (Fig. 21F) with 8 distal spines. Maxilla (Fig. 21G) semioval. Labium (Fig. 21H) lobe distolateral corner naked. Maxilliped (Fig. 21I) basis 0.9 L:W; endites partly merged, distal margin, with tubercles (gustatory cusps); palp article-2 inner margin, with three setae, outer margin with seta; article-3 with three setae; article-4 with six setae. Epignath not seen.

Cheliped (Fig. 22A) slender; basis 1.6 L:W, with distoproximal seta; merus with seta, carpus 2.3 L:W, with ventral and subproximal setae; chela non-forcipate; palm 1.3 L:W, with row of three setae on inner side; fixed finger distal spine pointed, regular size, with three ventral setae; dactylus 5 L:W, cutting edge smooth, proximal seta present.

Pereopod-1 (Fig. 22B) coxa with seta; basis 8.3 L:W, with two ventral and one dorsal seta; ischium with ventral seta; merus 2.2 L:W and, 0.7x carpus with two seta; carpus 2.5 L:W, 0.4x propodus; propodus 8.2 L:W, 1.3x dactylus and unguis combined length, with two setae; dactylus 0.7x unguis.

Pereopod-2 (Fig. 22C) coxa with seta; basis 9.1 L:W, 4.9x merus with two ventral seta and one dorsal seta; ischium with ventral seta; merus 1.9 L:W, 0.6x carpus, with seta; carpus 2.6 L:W, 0.7x propodus, with two simple and one blade-like spine, 0.5x propodus; propodus six L:W, 2x dactylus and unguis combined length, with distal seta and microtrichia on ventral margin; dactylus 0.6x unguis.

Pereopod-3 (Fig. 22D) basis 6.5 L:W, 4.1x merus, with one simple and one penicillate ventral setae and penicillate dorsal seta; ischium with ventral seta; merus 1.8 L:W, 0.8x carpus, with seta; carpus 2.6 L:W, 0.7x propodus, with blade-like spine 0.7x propodus; propodus 4.7 L:W, with seta.

Pereopod-4 (Fig. 22E) basis 7.0 L:W, 4.4x merus; ischium with ventral seta; merus 2.0 L:W, 0.5x carpus, with seta; carpus 3.6 L:W, 0.9x propodus, with one simple and one rod setae 0.3x propodus, one spine and one blade-like spine 0.2x propodus,; propodus 5.2 L:W, 2.6x dactylus and unguis combined length, with one simple and one serrate seta 1x propodus and microtrichia on ventral margin; dactylus 3.0x unguis.

Pereopod-5 (Fig. 22F) basis 7.8 L:W, 1.2x merus, with simple ventral seta and penicillate dorsal seta; ischium with ventral seta; merus 2.0 L:W, 0.6x carpus, with seta; carpus 4.0 L:W, 0.9x propodus, with three simple setae, one rod seta 0.4x propodus, and one blade-like spine 0.3x propodus; propodus 5.0 L:W, 1.9x dactylus and unguis combined length, with two ventral seta and one serrate dorsal seta 0.9x propodus and microtrichia on ventral margin; dactylus 0.2x unguis.

Pereopod-6 (Fig. 22G) basis 7.6 L:W, 5.2x merus; ischium with ventral seta; merus 2.0 L:W, 0.6x carpus, with two setae; carpus 3.5 L:W, propodus, with one simple, one sensory 0.4x propodus and one blade-like spine 0.3x propodus; propodus 4.0 L:W, 2.1x dactylus and unguis combined length, with two simple ventral setae, one simple, and one serrate dorsal setae 1.1x propodus; dactylus 1.6x unguis.

Pleopods (Fig. 22H) exopod with four, endopod with seven plumose setae.

Uropod (Fig. 22I) peduncle 1.2 L:W; exopod 0.8x endopod, with two articles; article-1 5.5 L:W, with seta; article-2 7.0 L:W, with two setae; endopod article-1 3.4 L:W, with two distal penicillate setae; article-2 4.0 L:W, with five setae.

**Distribution:** *P*. *yenneferae* n. sp. is known only from APEI3 of the Clarion and Clipperton Fractures Zone, Central Pacific.

**Remarks**: *P*. *yenneferae* n. sp. with short rod setae on pereopods 5–6 carpus can be distinguished from *P*. *longisetosus*, *P*. *longispinus*, *P*. *nipponicus*, *P*. *spatula*, *Pseudotanais* sp. O, *Pseudotanais romeo* and *P*. *julietae*, which have long rod setae on pereopods 5–6 carpus. Also, it can be distinguished from. *P*. *affinis*, *P*. *macrochelis* and *P. nordenskioldi*, *P*. *scalpellum*, *P*. *svavarssoni*, *P*. *vitjazi* and *Pseudotanais* sp. P (McLelland, 2007) by the wider molar of the mandible.

***Pseudotanais geralti*** **n**. **sp**.

Figures 23–25.

**Figure 23:**
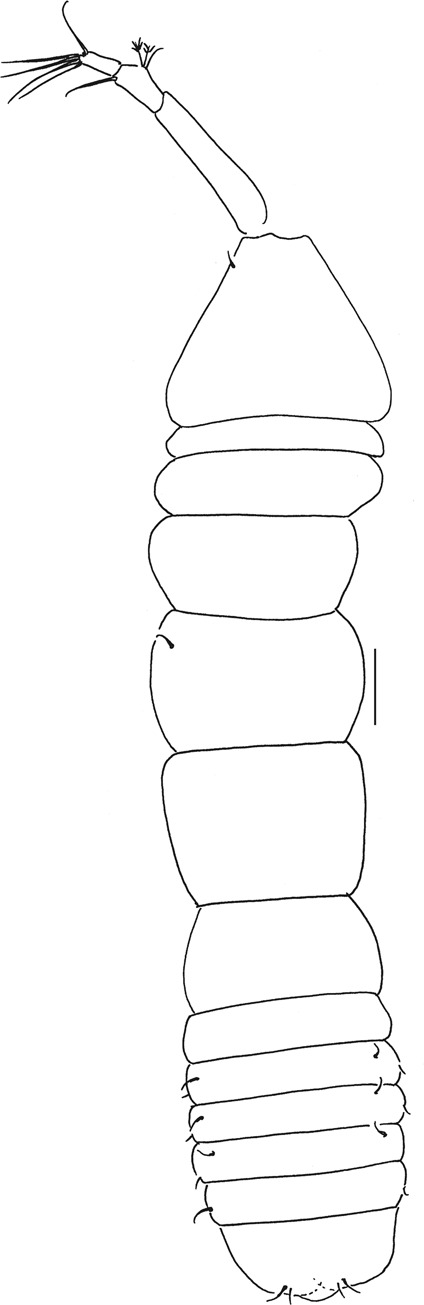
*Pseudotanais geralti* n. sp., ZMH K-56578, holotype neuter. Dorsal view. Scale bar: 1 mm.

**Figure 24:**
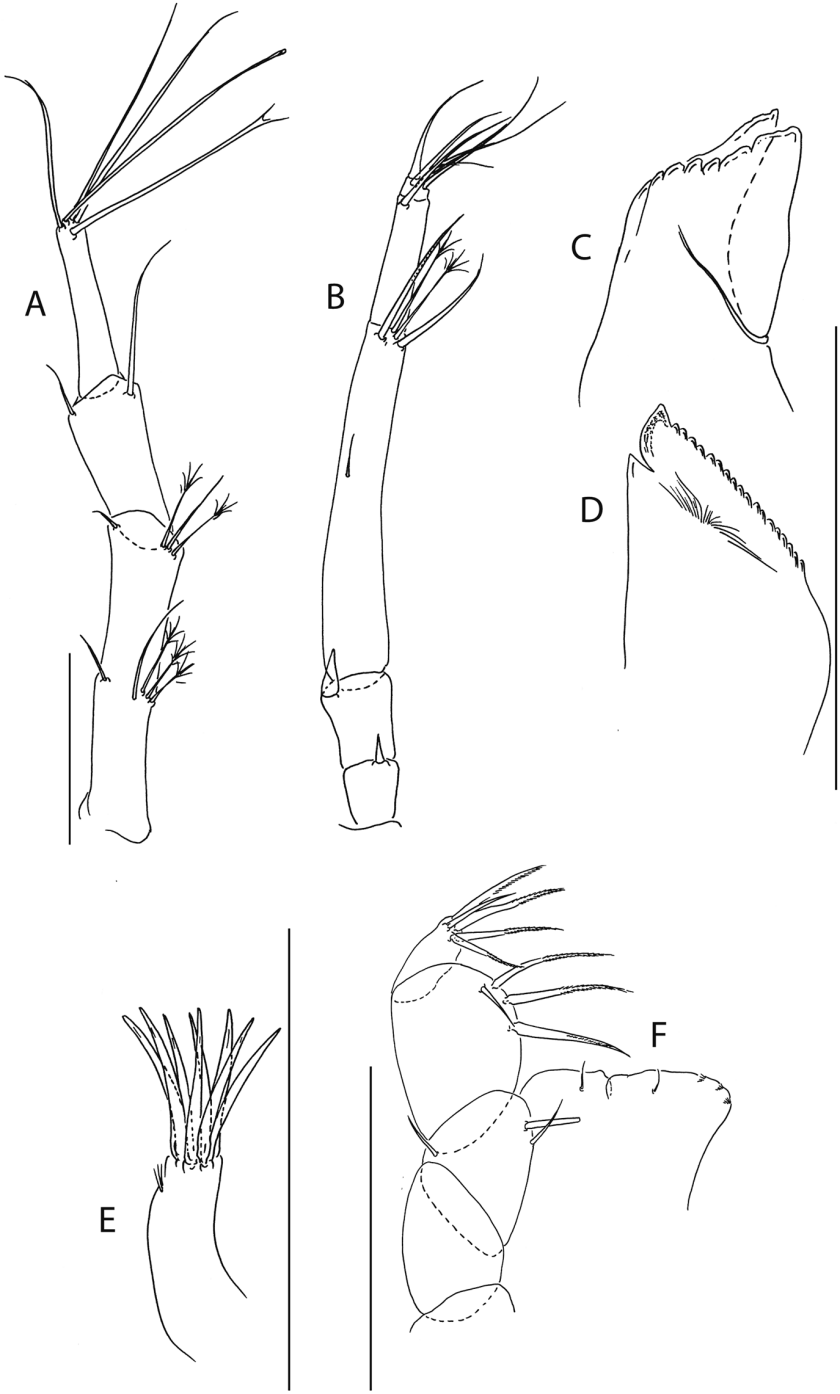
*Pseudotanais geralti* n. sp., ZMH K-56581, neuter. (**A**), antennule; (**B**), antenna; (**C**), left mandible; (**D**), right mandible; (**E**), maxillule; (**F**), maxilliped. Scale bar: 0.1 mm.

**Figure 25:**
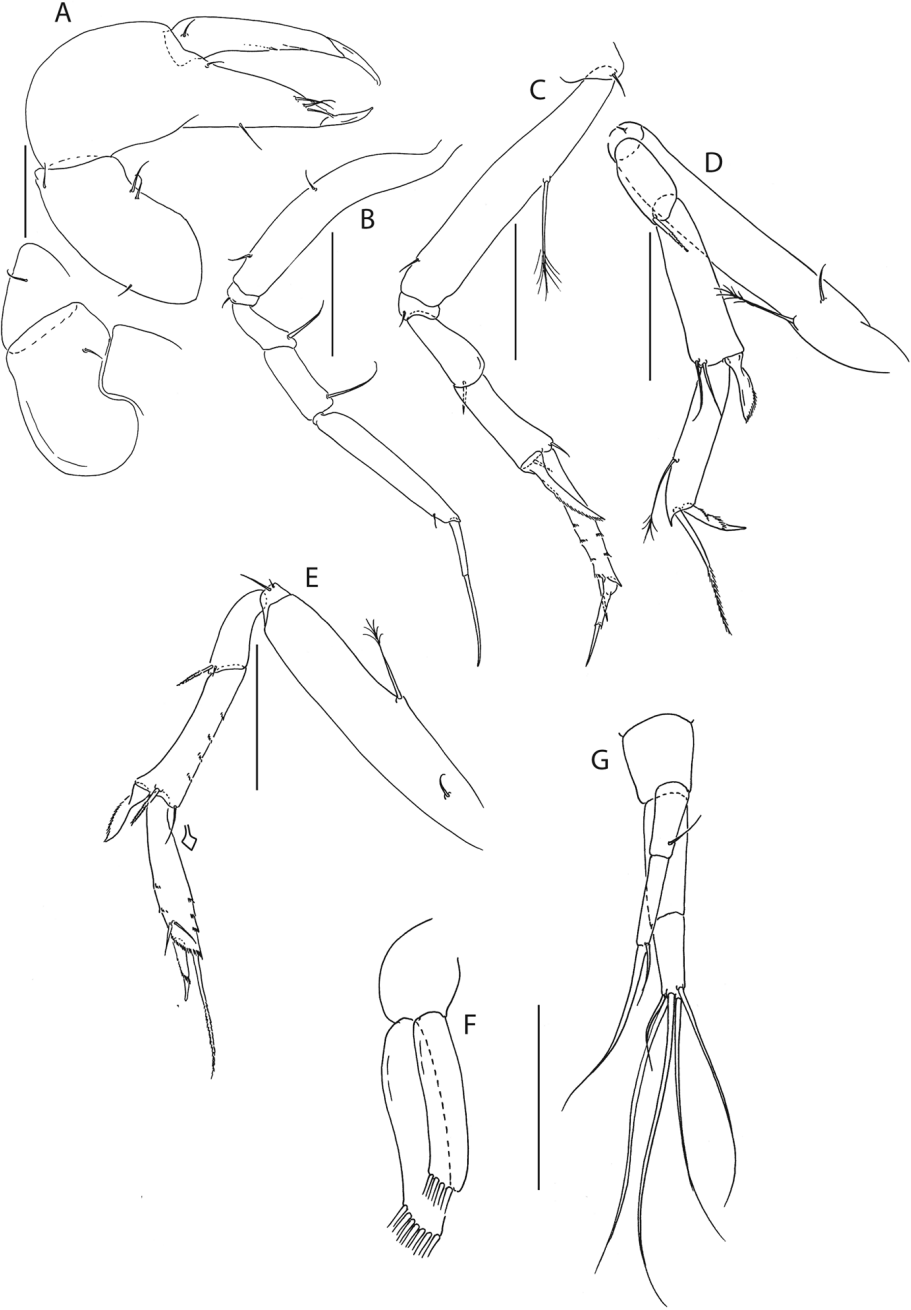
*Pseudotanais geralti* n. sp., ZMH K-56581, neuter. (**A**), cheliped; (**B**), pereopod-1; (**C**), pereopod-2; (**D**), pereopod-5; (**E**), pereopod-6; (**F**), pleopod; (**G**), uropod pereopod-6. Inset at (**E**) show detail of tip of the rod seta. Scale bar: 0.1 mm.

**Material examined:** Holotype: neuter, BL = 1.4 mm, ZMH K-56578 (partly disseceted). St 81, 11° 3.97′N 119° 37.67′W, 4365 m, 1 Apr 2015.

Paratypes: neuter, BL = 1.1 mm, ZMH K-56579 (partly dissected). St. 81, 11° 3.97′N 119° 37.67′W, 4365 m, 1 Apr 2015; three neuters, BL = 1.1–1.3 mm, ZMH K-56581 (dissected), ZMH K-56582, ZMH K-56583. St. 99, 11° 2.61′N 119° 39.52′W, 4401 m, 4 Apr 2015; neuter, BL = 1.1 mm, ZMH K-56580. St. 117, 13° 52.39′N 123° 15.30′W, 4496 m, 7 Apr 2015.

**Diagnosis:** Mandible molar wide. Pereopod-1 basis with two setae. Pereopod 5–6 carpus with short distodorsal rod seta.

**Etymology:** The species is named after the character from a Polish fantasy novel ‘Wiedźmin’ (eng. ‘The Witcher’) written by Andrzej Sapkowski.

**Description of neuter**. BL = 1.4 mm. Body slender (Fig. 23), 4.7 L:W. Carapace 0.8 L:W, 5.2x pereonite-1, 0.2x BL. Pereonites 0.5x BL, pereonites-1–6: 0.2, 0.3, 0.5, 0.6, 0.8 and 0.5 L:W, respectively. Pleon short, 0.2x BL. Pleonites 0.9 L:W.

Antennule (Fig. 24A) article-1 0.5x total length, 5.0 L:W, 1.9x article-2, with two simple and three mid-length penicillate setae, and two simple and two penicillate distal setae; article-2 2.5 L:W, 0.9x article-3, with two setae; article-3 5.7 L:W, with three simple, one bifurcate seta and one aestetasc.

Antenna (Fig. 24B) article-2 1.8 L:W; 1.1x article-3, with spine 0.3x article-2; article-3 1.3 L:W, article-4, with spine 0.6x article-3; article-4 1.4 L:W, 0.6x article-5, with simple mid-length seta, two simple and two penicillate distal setae; article-5 4.0 L:W, 7.0x article-6, with seta; article-6 0.8 L:W, with five setae.

Mouthparts. Left mandible (Fig. 24C) *lacinia mobilis* well developed and distally serrate, incisor distal margin gently serrate. Right mandible (Fig. 24D) incisor distal margin serrate, *lacina mobilis* merged to a small process. Maxillule (Fig. 24E) with 8 distal spines and three subdistal setae. Maxilliped (Fig. 24F) endites partly merged, distal margin without tubercles (gustatory cusps) and seta; palp article-1 naked; palp article-2 inner margin with two setae, outer margin, with seta; article-3 with four setae; article-4 with five setae.

Cheliped (Fig. 25A) slender; basis 1.7 L:W, with distoproximal seta; merus, with seta; carpus 2.39 L:W, with two ventral setae, and with distal and subproximal setae dorsally; chela non-forcipate; palm 1.5 L:W; fixed finger distal spine pointed, regular size, with three ventral setae; dactylus 5.3 L:W, cutting edge with two spines, proximal seta present.

Pereopod-1 (Fig. 25B) basis 6.1 L:W, with two ventral setae; ischium with ventral seta; merus 2.2 L:W, 0.7x carpus, with seta; carpus 2.6 L:W, 0.4x propodus, with seta; propodus 7.2 L:W, 1.2x dactylus and unguis combined length, with seta; dactylus 0.6x unguis.

Pereopod-2 (Fig. 25C) coxa with seta; basis 5.7 L:W, 3.1x merus, with ventral seta and penicillate dorsal seta; ischium with ventral seta; merus 2.5 L:W, 0.9x carpus, with seta; carpus 2.7 L:W, 0.7x propodus, with two simple setae and blade-like spine, 0.7x propodus; propodus 7.2 L:W, 1.2x dactylus and unguis combined length, with seta and microtrichia on ventral margin, dactylus 0.6x unguis.

Pereopod-5 (Fig. 25D) basis 6.4 L:W, 4.1x merus, with penicillate ventral seta and with simple dorsal seta; ischium with ventral seta; merus 1.7 L:W, 0.5x carpus, with seta; carpus 3.7 L:W, 1.2x propodus, with one simple seta, one sensory 0.4x propodus, and one blade-like spine 0.4x propodus; propodus 4.6 L:W, 2.1x dactylus and unguis combined length, with penicillate seta at mid-length and serrate seta distally; dactylus 0.1x unguis.

Pereopod-6 (Fig. 25E) basis 4.1 L:W, 3.4x merus, with one simple and one penicillate setae ventrally; ischium with one short and one long setae; merus 2.2 L:W, 0.6x carpus, with one short and one long serrate setae; carpus 3.7 L:W, 0.9x propodus, with one serrate, one rod setae 0.3x propodus and one blade-like spine 0.45x propodus; propodus 5.2 L:W, 2.4x dactylus and unguis combined length, with two ventral setae, and one serrate dorsal seta 0.8x propodus, and microtrichia on ventral margin; dactylus 1.6x unguis.

Pleopods (Fig. 25F) exopod with four; endopod with 7 plumose setae.

Uropod (Fig. 25G) 1.2 L:W; exopod 0.6x endopod, with two articles; article-1 3.2 L:W, with seta; article-2 4.7 L:W, with two setae; endopod article-1 3.1 L:W; article-2 2.8 L:W, with five setae.

**Distribution:** *P*. *geralti* n. sp. is known from the Belgium (GSR) and Interoceanmetal (IOM) licence areas of the Central Pacific.

**Remarks:** *P*. *geralti* can be distinguished from the other species in this group by the same characters as listed in *P*. *yenneferae*. *P*. *geralti* is morphologically closer to *P*. *yenneferae* from which is distinguished by its relatively long dorso-distal seta on merus of pereopod-1 (short in *P*. *yenneferae*), and shorter cheliped carpus (at least twice as wide in *P*. *yenneferae*).

$$ \mbox{'} {\rm{denticulatus}}+{\rm{abathagastor}}\mbox{'}\,{\rm{group}}$$

**Diagnosis:** Antenna article 2–3 with spines or setae. Mandible molar wide or acuminate. Chela non-forcipate. Pereopod-1 basis with few (1–3) setae. Merus and carpus distodorsal seta short. Pereopod-2 with short, semilong or long blade-like spine on carpus. Pereopods 5–6 carpus distodorsal seta short. Unguis of pereopod 4–6 elongated. Uropod slender, exopod longer or slightly shorter than endopod

**Species included:** *Pseudotanais corollatus* Bird & Holdich, 1984; *P*. *denticulatus* Bird & Holdich, 1984; *P*. *abathagastor* Błażewicz-Paszkowycz, Bamber & Jóźwiak, 2013; *Pseudotanais* sp. C (McLelland 2008); *Pseudotanais chopini* n. sp.; *Pseudotanais georgesandae* n. sp.; *Pseudotanais chaplini* n. sp.; *Pseudotanais oloughlini* n. sp.; *P*. *mariae* n. sp.

**Remarks:** The ‘*denticulatus + abathagasthor*’ group can be distinguished from the ‘affinis + longisetosus’ group by the presence of a long seta on merus pereopod-1 in the ‘affinis + longisetosus’ clade.

***Pseudotanais georgesandae*** **n**. **sp**.

Figures 26 and 27.

**Figure 26:**
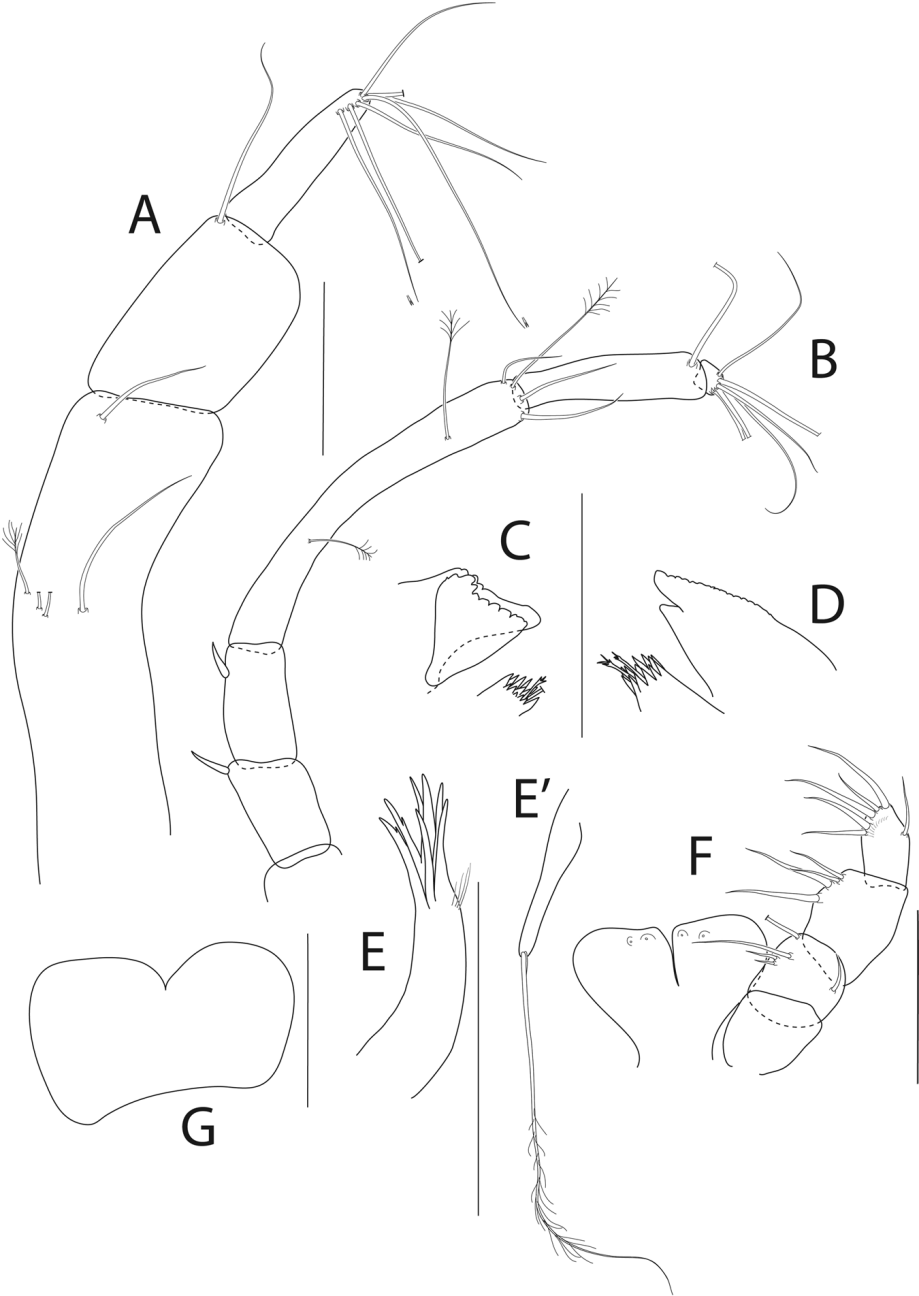
*Pseudotanais georgesandae* n. sp., ZMH K-56577, holotype neuter. (**A**), antennule; (**B**), antenna; (**C**), left mandible; (**D**), right mandible; (**E**), maxillule; E’, endit; (**F**), maxilliped (**G**), labium. Scale bar: 0.1 mm.

**Figure 27:**
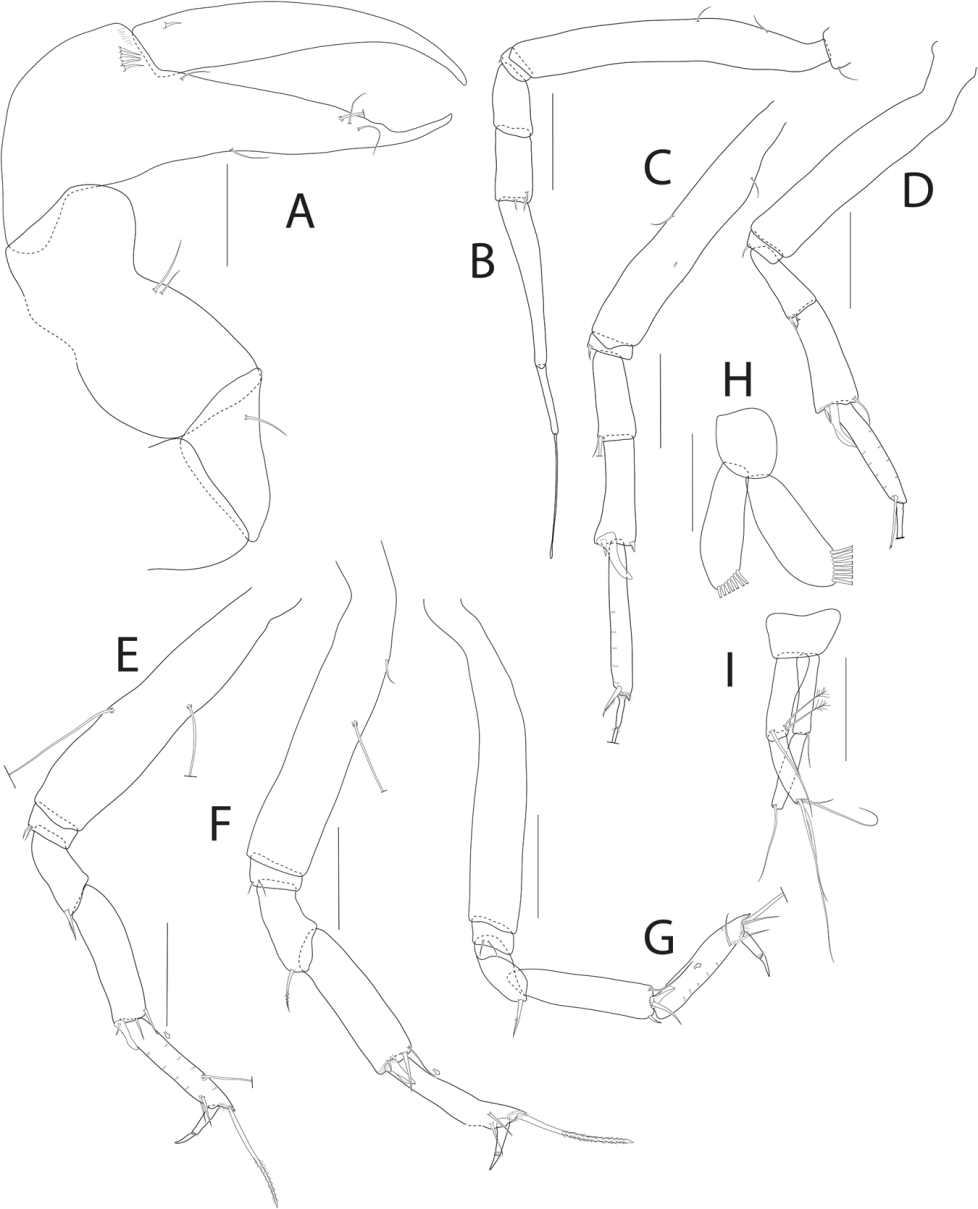
*Pseudotanais georgesandae* n. sp., ZMH K-56577, holotype neuter. (**A**), cheliped; (**B**), pereopod-1; (**C**), pereopod-2; (**D**), pereopod-3; (**E**), pereopod-4; (**F**), pereopod-5; (**G**), pereopod-6; (**H**), pleopod; (**I**), uropod. Insets at (**E**,**F**) show detail of tip of the rod seta. Scale bar: 0.1 mm.

**Material examined:** Holotype: neuter BL = 1.5 mm, ZMH K-56577 (partly dissected). St 192, 18° 44.81′N 128° 21.87′W, 4877 m, 21 Apr 2015.

**Diagnosis:** Mandible molar wide. Antenna article 2 and 3 with spine. Pereopod-2 carpus with short blade-like spine. Uropod exopod slightly shorter than endopod.

**Etymology:** The species is named in recognition of Amantine Lucile Aurore Dupin known as George Sand, a French novelist and essayist, well known for her partnership with the composer and pianist Frédéric Chopin.

**Description of neuter**. Antennule (Fig. 26A) 3.2 L:W, 2.3x article-2, article-2 1.4 L:W, 1.1x article-3, article-3 4.0 L:W, with five simple and two bifurcate setae.

Antenna (Fig. 26B) 1.4 L:W; article-2 0.8x article-3; article-3 1.7 L:W, 0.3x article-4; article-4 8.4 L:W, 2.0x article-5; article-5 4.0 L:W, 8.0x article-6; article-6 wide.

Mouthparts. Left mandible (Fig. 26C) *lacinia mobilis* well developed and serrate distally. Right mandible (Fig. 26D) molar wide with two spines in the middle. Maxillule (Fig. 26E,E’) with five simple and two bifurcate distal spines with four subdistal setae. Maxilliped (Fig. 26F) endites merged with groove in the mid-length, distal margin with two tubercles (gustatory cusps); palp article-2 inner margin with four setae, outer margin with seta; article-3 with four setae, article-4 with five setae on inner margin and one seta on outer margin. Labium (Fig. 26G) lobes distolateral corner naked.

Cheliped (Fig. 27A) slender; carpus 1.8 L:W, with two ventral setae; chela non-forcipate; palm 1.8 L:W, 1.2x palm; dactylus 5.7 L:W with proximal seta.

Pereopod-1 (Fig. 27B) basis 7.7 L:W; merus 1.6 L:W, 0.8x carpus; carpus 2.3 L:W, 0.4x propodus with three setae; propodus 6.3 L:W, 0.8x dactylus and unguis combined length; dactylus 0.6x unguis.

Pereopod-2 (Fig. 27C) basis 5.9 L:W, 3.1x merus; merus 2.8 L:W, 0.8x carpus; carpus 3.4 L:W, 0.7x propodus, with blade-like spine 0.3x propodus; propodus 7.0 L:W.

Pereopod-3 (Fig. 27D) basis 6.0 L:W, 3.3x merus; ischium with seta; merus 2.6 L:W, 0.8x carpus with one simple seta and one serrate spine; carpus 2.8 L:W, 0.8x propodus with one simple seta, one serrate seta, one spine and one blade-like spine 0.4x propodus; propodus 5.4 L:W with serrate spine and microtrichia on ventral margin.

Pereopod-4 (Fig. 27E) basis 5.0 L:W, 4.0x merus with two plumoe setae; ischum with two setae; merus 2.2 L:W, 0.6x carpus with one serrate spine; carpus 3.2 L:W, propodus, with one simple seta, one rod seta 0.2x propodus, one serrate spine and one blade-like spine 0.2x propodus; propodus 5.8 L:W, 2.2x dactylus and unguis combined length with serrate seta 0.9x propodus.

Pereopod-5 (Fig. 27F) basis 5.7 L:W, 3.7x merus; merus 2.2 L:W, 0.6x carpus; carpus 3.3 L:W, 1.2x propodus, with one rod seta 0.3x propodus and one blade-like spine 0.2x propodus; propodus 5.3 L:W, 2.6x dactylus and unguis combined length with serrate seta 0.9x propodus; dactylus 2.0x unguis.

Pereopod-6 (Fig. 27G) basis 6.4 L:W, 4.8x merus; merus 2.0 L:W, 0.5x carpus; carpus 4.0 L:W, 1.2x propodus, with one rod seta 0.5x propodus and one blade-like spine 0.2x propodus; propodus 4.5 L:W, 2.2x dactylus and unguis combined length; dactylus 1.4x unguis.

Pleopods (Fig. 27H) exopod with seven, endopod with eight plumose setae.

Uropod (Fig. 27I) peduncle 1.5 L:W; exopod article-1 6.0 L:W with seta; article-2 5.6 L:W with at least one seta (other broken); endopod article-1 3.5 L:W with one simple and two penicillate; article-2 4.0 L:W with four setae. Exopod 0.9x endopod.

**Distribution:** *P*. *georgesandae* n. sp. is known only from APEI3 of the Clarion and Clipperton Fractures Zone, Central Pacific.

**Remarks**: *Pseudotanais georgesandae* n. sp. can be distinghuished from all the other members of the ‘denticulatus + abathagastor’ group by the wide mandible molar. The molar of *P*. *georgesandae* has two bifurcate long spines, which are absent in *P*. *corollatus* and *P*. *denticulatus*. The molar of *Pseudotanais* sp. C has one straight spine.

***Pseudotanais chopini*** **n**. **sp**.

Figures 28–30.

**Figure 28:**
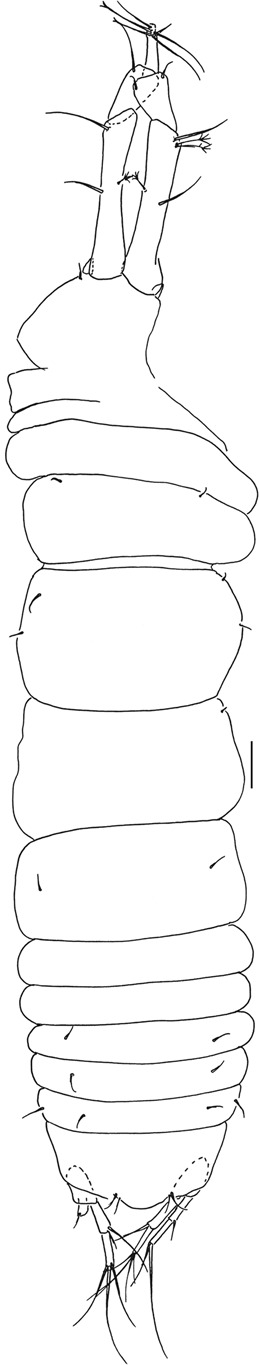
*Pseudotanais chopini* n. sp., ZMH K-56568, holotype neuter. Dorsal view. Scale bar: 1 mm.

**Figure 29:**
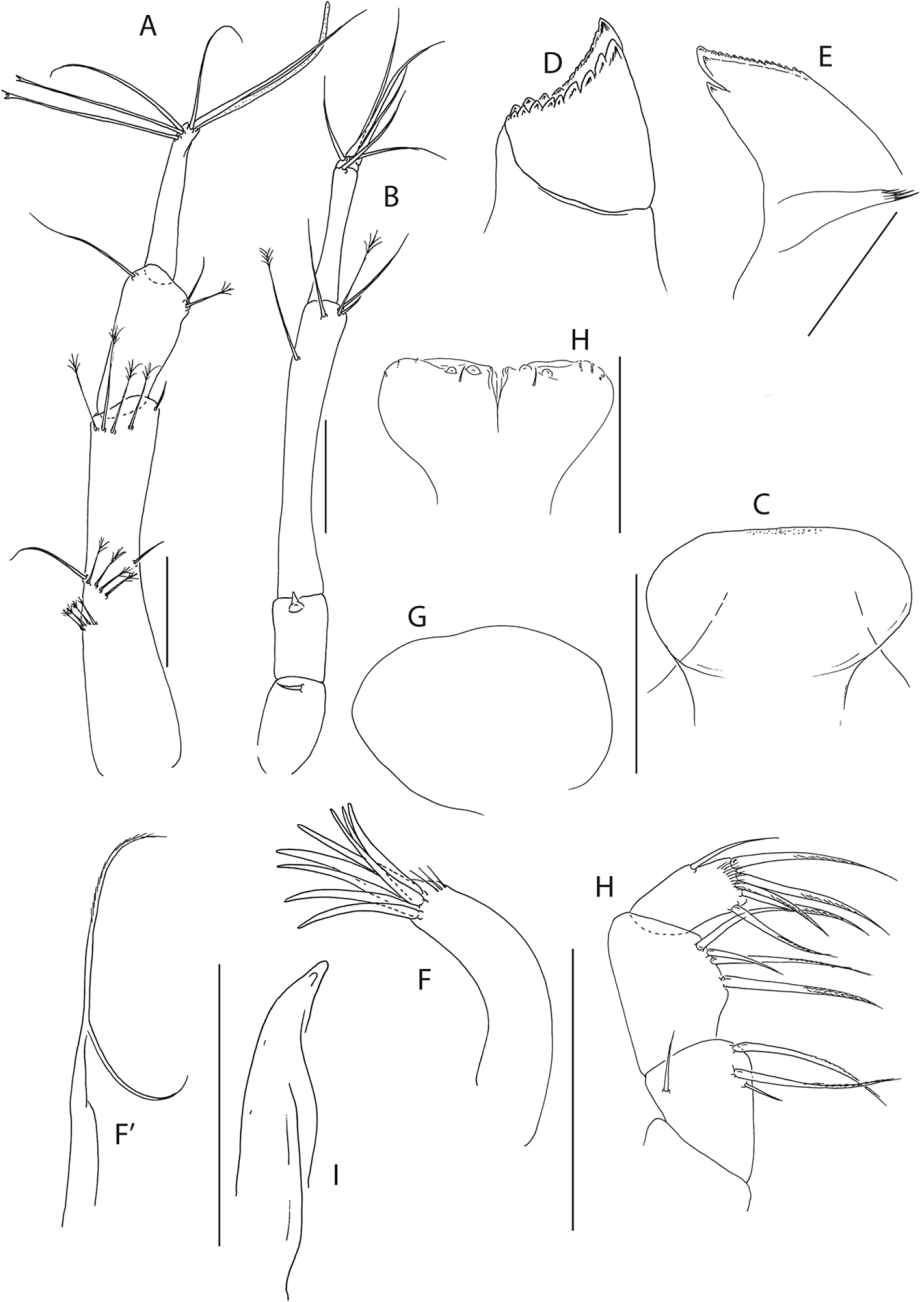
*Pseudotanais chopini* n. sp., ZMH K-56573, neuter. (**A**), antennule; (**B**), antenna; (**C**), labrum; (**D**), left mandible; (**E**), right mandible; (**F**), maxillule; F’ endit; (**G**), maxilla; (**H**), maxilliped: (**I**), epignath. Scale bar: 0.1 mm.

**Figure 30:**
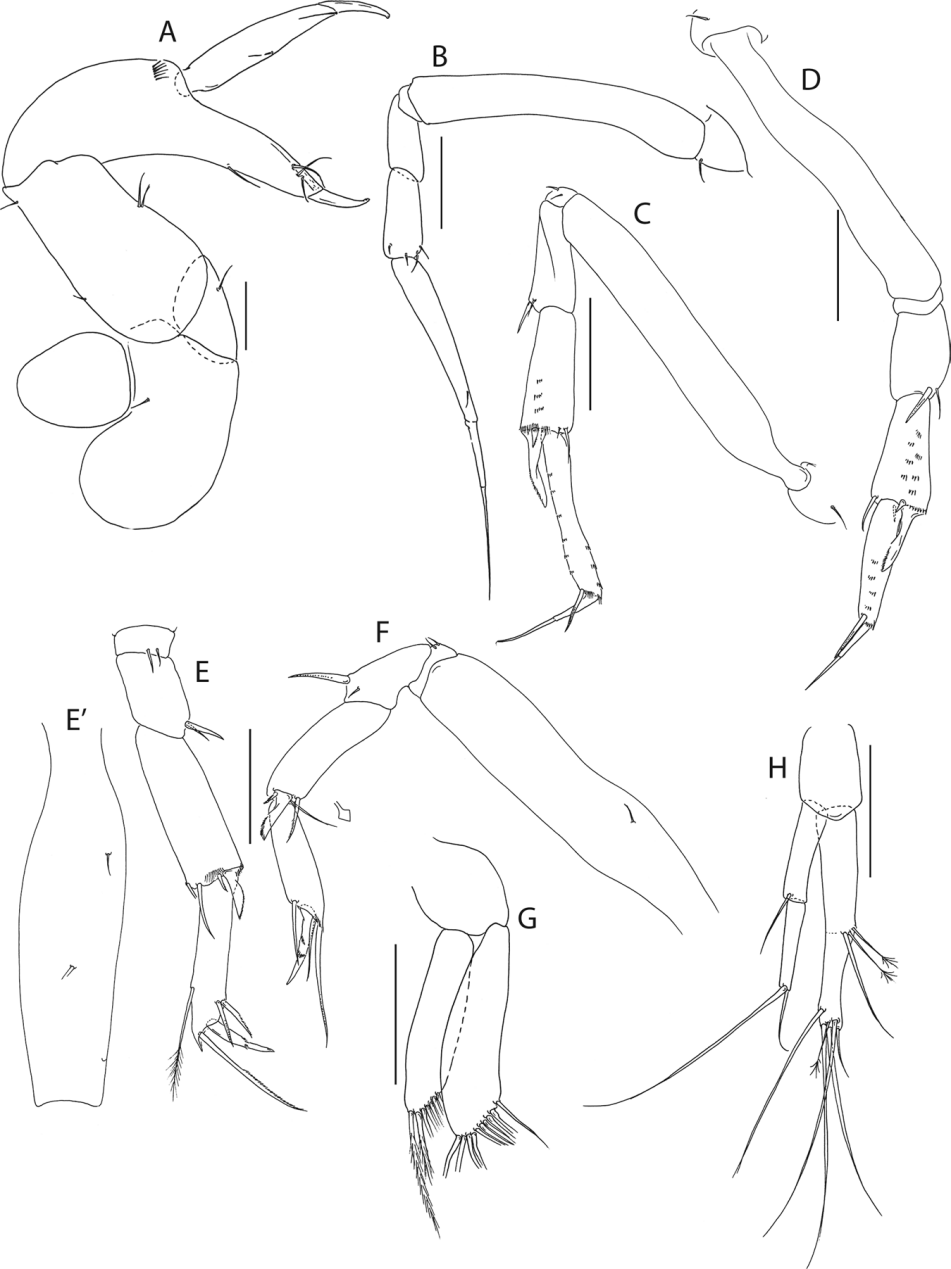
*Pseudotanais chopini* n. sp., ZMH K-56573, neuter. (**A**), cheliped; (**B**), pereopod-1; (**C**), pereopod-2; (**D**), pereopod-3; (**E**), pereopod-4; E’ basis of pereopod-3; (**F**), pereopod-6; (**G**), pleopod; (**H**), uropod. Inset at (**F**) show detail of tip of the rod seta. Scale bar: 0.1 mm.

**Material examined:** Holotype: neuter, BL = 1.9 mm, ZMH K-56568. St 24, 11° 51.52′N 117° 1.19′W, 4100 m, 22 Mar 2015.

**Paratypes:** three neuters, BL = 1.1–2 mm, ZMH K-56565, ZMH K-56566, ZMH K-56567. St 20, 11° 49.81′N 117° 0.28′W, 4093 m, 22 Mar 2015; two neuters, BL = 1.5–2 mm, ZMH K-56569, ZMH K-56570. St 24, 11° 51.52′N 117° 1.19′W, 4100 m, 22 Mar 2015; two neuters BL = 1.8–1.9 mm, ZMH K-56573 (dissected), ZMH K-56574. 50, 11° 49.92′N 117° 29.31′W, 4330 m, 27 Mar 2015; two neuters, BL = 1.2–1.3 mm, ZMH K-56571, ZMH K-56572. St 59, 11° 48.55′N 117° 29.03′W, 4342 m, 28 Mar 2015; neuter, BL = 1.2 mm, ZMH K-56575. St 99, 11° 2.61′N 119° 39.52′W, 4401 m, 4 Apr 2015.

**Diagnosis:** Mandible molar acuminate. Antenna article 2 and 3 with spine. Pereopod-2 with semilong blade-like spine. Uropod exopod slightly shorter than endopod.

**Etymology:** The species is dedicated to Frédéric Chopin, a Polish composer and virtuoso pianist.

**Description**. BL = 1.9 mm. Body robust (Fig. 28), 3.7 L:W. Carapace 0.6 L:W, 6.2x pereonite-1, 0.1x BL. Pereonites 0.58x BL, pereonites-1–6: 0.1, 0.2, 0.4, 0.6, 0.5 and 0.5 L:W, respectively. Pleon short, 0.2x BL. Pleonites 0.8 L:W.

Antennule (Fig. 29A) article-1 0.5x total length, 6.0 L:W, 2.8x article-2, with two simple and nine penicillate mid-length setae, one simple and four penicillate distal setae; article-2 2.0 L:W, 0.8x article-3, with two simple and one penicillate distal setae; article-3 6.8 L:W, with three simple, two bifurcate setae and one aestetasc.

Antenna (Fig. 29B) 1.7 L:W; article-2 1.2x article-3, with seta, 0.3x the article; article-3 1.3 L:W, 0.3x article-4, with spine 0.2x the article; article-4 6.9 L:W, 2.2x article-5, with penicillate subdistal seta, and three simple and one penicillate setae distally; article-5 4.7 L:W, 14x article-6, with distal seta; article-6 0.4 L:W, with five simple setae.

Mouthparts. Labrum (Fig. 29C) hood-shaped, naked. Left mandible (Fig. 29D) *lacinia mobilis* well developed and serrate distally, incisor distal margin gently serrate molar broken during dissection. Right mandible (Fig. 29E) incisor distal margin serrate, *lacina mobilis* merged to a small process. Maxillule (Fig. 29F,F’) with eight distal spines and three subdistal setae, endite with two setae. Maxilla (Fig. 29G) semioval. Maxilliped (Fig. 29H,H’) endites merged with groove in the mid-length, distal margin with two tubercles (gustatory cusps) and with seta; palp article-2 inner margin with three setae, outer margin with seta; article-3 with three setae, article-4 with six setae. Epignath (Fig. 29I) distally pointed.

Cheliped (Fig. 30A) basis 1.6 L:W, with distoproximal seta; merus with seta; carpus 2.3 L:W, with two ventral setae, and with one dorsodistal and one dorsosubproximal setae; chela non-forcipate; palm 2.2 L:W, with row of six setae on inner side; fixed finger distal spine pointed, with three ventral setae; dactylus 6.7 L:W.

Pereopod-1 (Fig. 30B) coxa with seta; basis 6.8 L:W; merus 2.4 L:W and 0.9x carpus; carpus 2.5 L:W, 0.7x propodus, with four setae; propodus 6.8 L:W, 1.5x dactylus and unguis combined length, with seta; dactylus 0.8x unguis.

Pereopod-2 (Fig. 30C) coxa with seta; basis 6.7 L:W, 3.9x merus; ischium with two ventral setae; merus 1.42 L:W, 0.8x carpus, with two setae; carpus 1.8 L:W, 0.9x propodus, with two setae, one spine and one blade-like spine 0.5x propodus; propodus 6.8 L:W, 1.5x dactylus and unguis combined length, with seta and microtrichia on ventral margin; dactylus 0.7x unguis.

Pereopod-3 (Fig. 30D) coxa with seta; basis 6.7 L:W, 3.9x merus; merus 1.4 L:W, 0.8x carpus, with two setae; carpus 1.8 L:W, 0.9x propodus, with two simple setae, one spine and one blade-like spine 0.6x propodus; propodus 4.2 L:W, 1.4x dactylus and unguis combined length, with seta and microtrichia on ventral margin; dactylus as long as unguis.

Pereopod-4 (Fig. 30E,E’) basis 3.8 L:W, 4.5x merus, with two simple ventral setae; ischium with two ventral setae; merus 1.5 L:W, 0.5x carpus, with two setae; carpus 0.9 L:W, 1.1x propodus, with one simple and one sensory (broken) setae, and with one spine and one blade-like spine 0.3x propodus; propodus 4.7 L:W, 2.1x dactylus and unguis combined length, with two serrrated setae on ventral margin, one penicillate and one serrate seta on dorsal margin 1x propodus; dactylus 1.7x unguis.

Pereopod-6 (Fig. 30F) basis 5.0 L:W, 3.7x merus, with ventral seta; ischium with two ventral seta; merus 1.8 L:W, 0.7x carpus, with two setae; carpus 3.0 L:W, 1.1x propodus, with one serrate and one rod setae 0.4x propodus, and with one spine and one blade-like spine 0.4x propodus; propodus 3.1 L:W, 1.6x dactylus and unguis combined length, with simple ventral seta and two serrate dorsal setae; dactylus 1.7x unguis.

Pleopods (Fig. 30G) exopod with seven, endopod with ten plumose setae.

Uropod (Fig. 30H) peduncle 1.5 L:W, exopod with two articles, 0.9x endopod; article-1 4.0 L:W, with simple seta; article-2 6 L:W, with two setae; endopod article-1 3.6 L:W, with one simple and two penicillate setae; article-2 3.8 L:W, with five simple and one penicillate seta.

**Distribution:** *P*. *chopini* n. sp. is known from the Belgium (GSR) and Interoceanmetal (IOM) licence areas of the Central Pacific.

**Remarks:** The acuminate mandible molar distinguishes *P*. *chopini* from other members of the ‘denticulatus + abathagastor’ group, such as *P*. *abathagastor*, *P*. *corollatus*, *P*. *denticulatus* and *P*. *georgesandae*, which have wide molars. *Pseudotanais chopini* can be further distinguished from *Pseudotanais* sp. C by the presence of a semilong (0.5x propodus) blade-like spine in pereopod-2 (long in *Pseudotanais* sp. C).

***Pseudotanais chaplini*** **n**. **sp**.

Figures 31 and 32.

**Figure 31:**
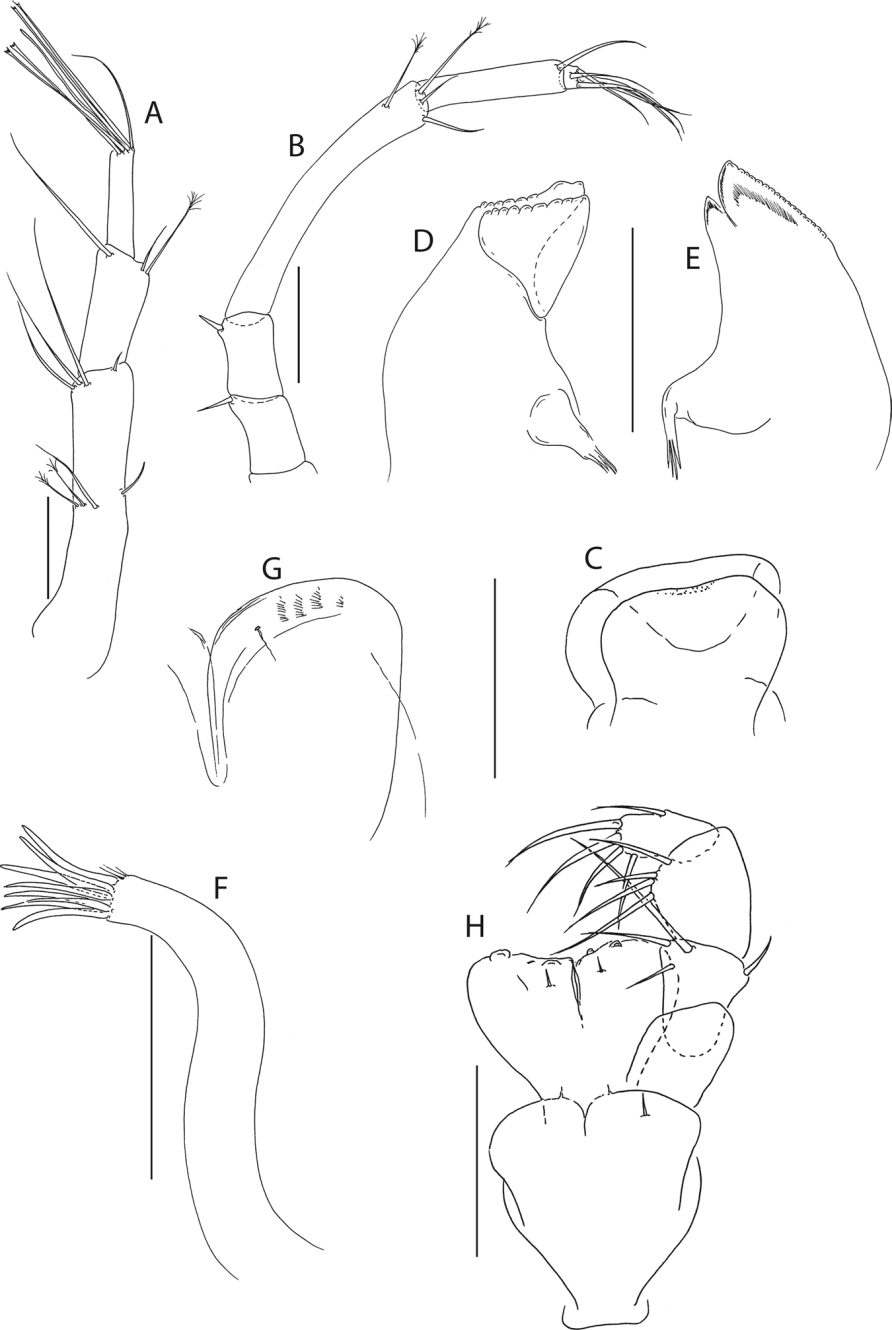
*Pseudotanais chaplini* n. sp., ZMH K-56564, holotype neuter. (**A**), antennule; (**B**), antenna; (**C**), labrium; (**D**), left mandible; (**E**), right mandible; (**F**), maxillule; (**G**), labium; (**H**), maxilliped. Scale bar: 0.1 mm.

**Figure 32:**
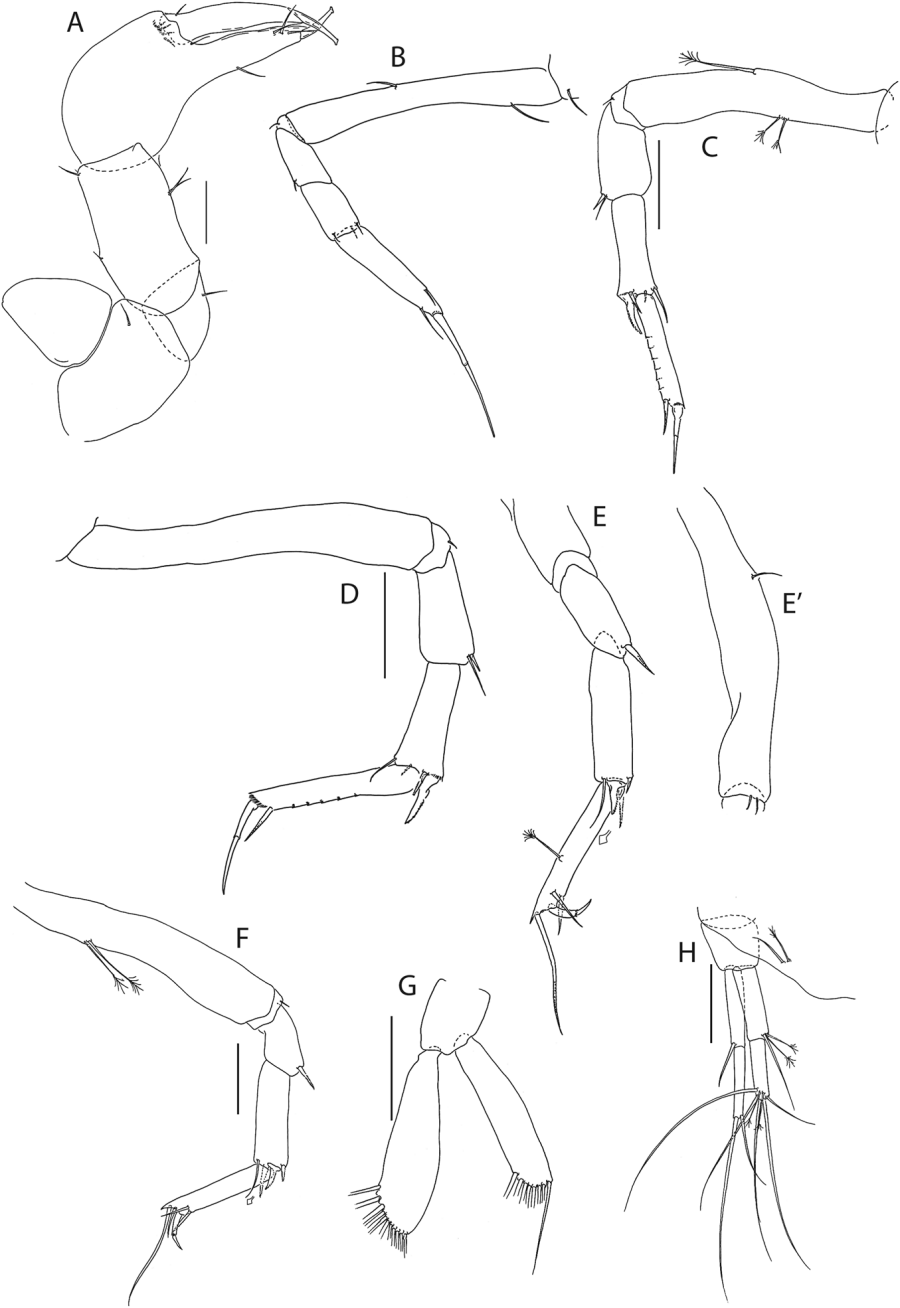
*Pseudotanais chaplini* n. sp., ZMH K-56564, holotype neuter. (**A**), cheliped; (**B**), pereopod-1; (**C**), pereopod-2; (**D**), pereopod-3; (**E**), pereopod-4; E’ basis of pereopod-4; (**F**) pereopod-6; (**G**), pleopod; (**H**), uropod. Insets at (**E**,**F**) show detail of tip of the rod seta. Scale bar: 0.1 mm.

**Material examined:** Holotype: neuter, BL = 1.5 mm, ZMH K-56564 (partly dissected). St 158, 14° 3.41′N 130° 7.99′W, 4946 m, 15 Apr 2015.

Paratypes: neuter, BL = 1.5 mm, ZMH K-56563 (partly dissected). St 20, 11° 49.81′N 117° 0.28′W, 4093 m, 22 Mar 2015.

**Diagnosis:** Antenna articles 2–3 with spines. Pereopod 2 and 3 carpus with short blade-like spine. Uropod exopod longer than endopod.

**Etymology:** The name of the species is dedicated to the great actor and film director of the silent film epoch Charles ‘Charlie’ Chaplin.

**Description**. Antennule (Fig. 31A) article-1 0.6x total length, 4.6 L:W, 2.6x article-2, with two simple and two penicillate mid-length setae and four distal setae; article-2 2.3 L:W, 1.1x article-3, with one penicillate and two simple setae; article-3 4.0 L:W, with one simple, four bifurcate setae, and one aestetasc.

Antenna (Fig. 31B) article-2 1.5 L:W; article-2 0.8x article-3, with spine 0.5x article-2; article-3 1.8 L:W, 0.3x article-4, with spine 0.3x article-3; article-4 8.6 L:W, 2.0x article-5, with two simple and two penicillate setae; article-5 5.0 L:W, 10.0x article-6, with seta; article-6 0.6 L:W, with six setae.

Mouthparts. Labrum (Fig. 31C) hood-shaped, setose. Left mandible (Fig. 31D) *lacinia mobilis* well developed and serrate distally, molar acuminate. Right mandible (Fig. 31E) incisor distal margin serrate, *lacina mobilis* merged to a small process. Maxillule (Fig. 31F) with 8 distal spines. Labium (Fig. 31G) distolateral corner lobes weakly setose. Maxilliped (Fig. 31H) distal margin with two tubercles (gustatory cusps) and seta; palp article-2 inner margin with three inner setae, outer margin with seta; article-3 with four setae, article-4 with five setae.

Cheliped (Fig. 32A) slender; basis 1.5 L:W, with distoproximal seta; merus with simple seta; carpus 2.1 L:W, with two ventral setae, and with one subdistal and one subproximal setae; chela non-forcipate; palm 1.2 L:W, with row of five setae on inner side; fixed finger distal spine pointed, 1.2x palm, with three ventral setae; dactylus 6.7 L:W, cutting edge smooth, proximal seta present.

Pereopod-1 (Fig. 32B) coxa with seta; basis 7.2 L:W, with one ventral and one dorsal setae; ischium with ventral seta; merus 1.7 L:W; carpus, with short seta; carpus 1.5 L:W, 0.4x propodus, with three short setae; propodus 4.6 L:W, 0.9x dactylus and unguis combined length, with two subdistal setae and one distal seta; dactylus 0.7x unguis, without proximal seta.

Pereopod-2 (Fig. 32C) basis 5.7 L:W, 3.6x merus; one ventral and two dorsal penicillate setae; ischium with ventral seta; merus 1.6 L:W, 0.7x carpus, with one seta and one spine; carpus 3.7 L:W, 0.9x propodus, with two simple setae, one serrate spine and one blade-like spine 0.4x propodus; propodus 6.2 L:W, 1.7x dactylus and unguis combined length, with seta and microtrichia on ventral margin; dactylus 0.9x unguis.

Pereopod-3 (Fig. 32D) basis 7.6 L:W, 3.4x merus; ischium with ventral seta; merus 2.2 L:W, 0.9x carpus, with simple seta and spine; carpus 2.8 L:W, 0.6x propodus, with two setae, one spine and one blade-like spine 0.3x propodus; propodus 7.0 L:W, 1.7x dactylus and unguis combined length, with seta and microtrichia on ventral margin; dactylus 0.7x unguis.

Pereopod-4 (Fig. 32E,E’) basis 6.2 L:W, 3.1x merus, with ventral seta; ischium with two ventral setae; merus 2.4 L:W, 0.7x carpus, with serrate seta; carpus 4.0 L:W, propodus, with one simple, one rod seta, one spine and blade-like spine, 0.2x propodus, rod seta 0.2x propodus; propodus 6.4 L:W, 2.9x dactylus and unguis combined length, with two ventral setae and one serrate dorsal seta 0.8x propodus; dactylus 1.7x unguis.

Pereopod-6 (Fig. 32F) basis 6.0 L:W, 4.3x merus, with two penicillate dorsal setae; ischium with ventral seta; merus 1.7 L:W, 0.6x carpus, with serrate seta; carpus 3.8 L:W, propodus, with rod seta 0.4x propodus, two spines and blade-like spine 0.2x propodus; propodus 5.5 L:W, 2.7x dactylus and unguis combined length, with one seta, two ventral spines ventrally and one serrate seta 1x propodus; dactylus 1.4x unguis.

Pleopods (Fig. 32G) exopod with eight, endopod with eleven plumose setae.

Uropod (Fig. 32H) peduncle 1.2 L:W; exopod with two articles, 1.1x endopod; article-1 5.7 L:W, with seta; article-2 7.5 L:W, with two simple setae; endopod article-1 3.2 L:W, with one simple and two penicillate setae; article-2 4.3 L:W, with two penicillate and five simple setae.

**Distribution:** *P*. *chaplini* n. sp. is known from the IFREMER and IOM licence areas of the Central Pacific.

**Remarks:** The exopod uropod being longer than endopod allows for distinguishing the new species from *P*. *abathagastor*, *P*. *corollatus*, *P*. *denticulatus*, *P*. *georgesandae*, *P. chopini* and *Pseudotanais* sp. C, as well as from all other species of the genus *Pseudotanais*.

***Pseudotanais oloughlini*** **n**. **sp**.

Figures 33–35.

**Figure 33:**
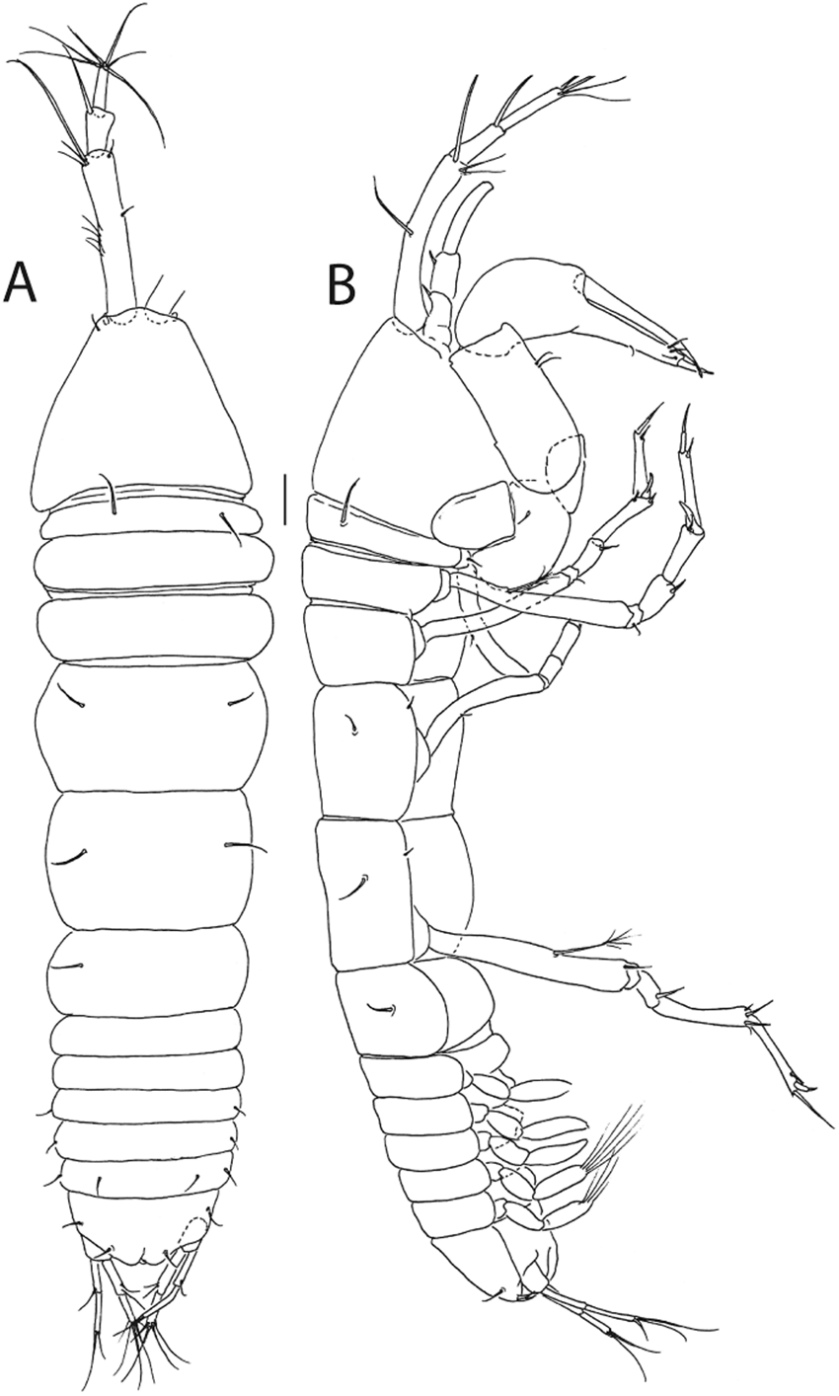
*Pseudotanais oloughlini* n. sp., ZMH K-56596, holotype neuter. (**A**), dorsal view; (**B**) lateral view. Scale bar: 1 mm.

**Figure 34:**
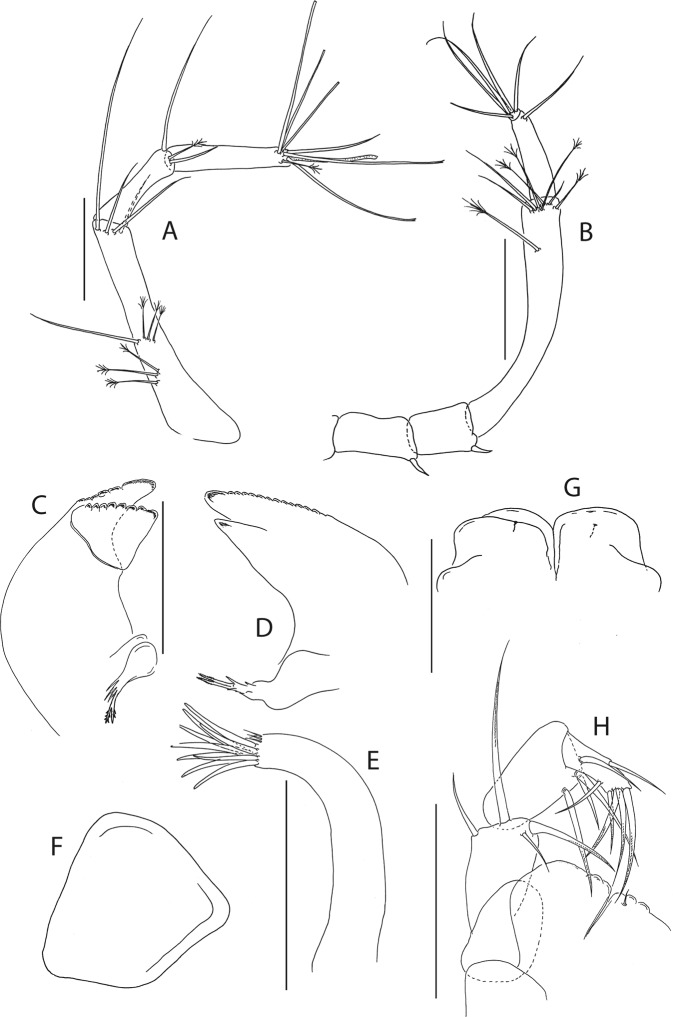
*Pseudotanais oloughlini* n. sp., ZMH K-56595, neuter. (**A**), antennule; (**B**), antenna; (**C**), left mandible; (**D**), right mandible; (**E**), maxillule; (**F**), maxilla; (**G**), labium; (**H**), maxilliped. Scale bar: 0.1 mm.

**Figure 35:**
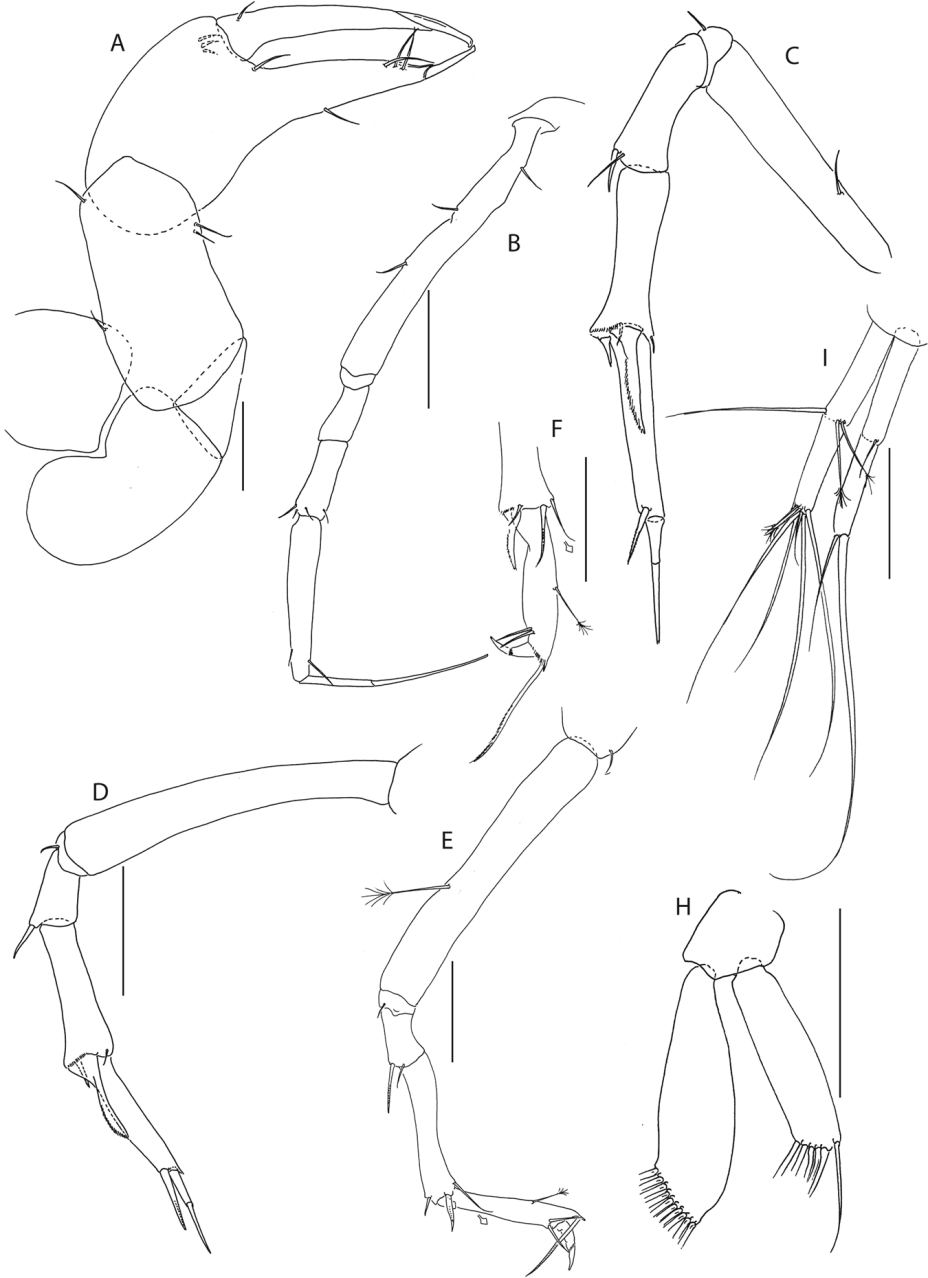
*Pseudotanais oloughlini* n. sp., ZMH K-56595, neuter. (**A**), cheliped; (**B**), pereopod-1; (**C**), pereopod-2; (**D**), pereopod-3; (**E**), pereopod-4; (**F**), pereopod-5; (**G**), pleopod; (**H**), uropod. Inset at (**E**) show detail of tip of the rod seta. Scale bar: 0.1 mm.

**Material examined:** Holotype: neuter, BL = 1.9 mm, ZMH K-56596. St 197, 18° 48.66′N 128° 22.75′W, 4805 m, 21 Apr 2015.

Paratypes: two neuters, BL = 2 mm, ZMH K-56597, ZMH K-56598. St 192, 18° 44.81′N 128° 21.87′W, 4877 m, 21 Apr 2015; two neuters, BL = 2–2.6 mm, ZMH K-56594 (dissected), ZMH K-56595 (dissected). St 197, 18° 48.66′N 128° 22.75′W, 4805 m, 21 Apr 2015.

**Diagnosis**: Mandible molar acuminate with bifurcate distal tooth. Antennal articles 2–3 with spine. Pereopods 2 and carpus with long blade-like spine. Uropod exopod longer than endopod.

**Etymology**: The species is named in recognition of the great holothurian specialist and wonderful friend and colleague – Dr. Mark O’Loughlin.

**Description of neuter**. BL 1.9 mm. Body slender (Fig. 33A,B), 3.9 L:W. Carapace 0.8 L:W, 5.4x pereonite-1, 0.2x BL. Pereonites 0.5x BL, pereonites-1–6: 0.1, 0.2, 0.2, 0.5, 0.6 and 0.4 L:W, respectively. Pleon short, 0.2x BL. Pleonites 0.9 L:W.

Antennule (Fig. 34A) article-1 0.5x total length, 6.0 L:W, 2.4x article-2, with one simple and six penicillate mid-length setae, and four simple setae (one very long); article-2 4.2 L:W, 0.8x article-3, with two simple and one penicillate seta; article-3 5.5 L:W, with one simple, two bifurcate, one penicillate and three broken setae, and one aestetasc.

Antenna (Fig. 34B) article-2 2.1 L:W; article-2 1.2x article-3, with spine 0.3x article-2; article-3 1.6 L:W, 0.3x article-4, with spine 0.3x article-3; article-4 6.8 L:W, 3.1x article-5, with penicillate subdistal seta and three simple, four penicillate distal setae; article-5 3.7 L:W, 11.0x article-6, with seta; article-6 0.5 L:W, with five setae.

Mouthparts. Left mandible (Fig. 34C) *lacinia mobilis* well developed and distally serrate, incisor distal margin serrate, molar acuminate, with distal bifurcate spine. Right mandible (Fig. 34D) incisor distal margin serrate, *lacina mobilis* merged to a small process. Maxillule (Fig. 34E) with 9 distal spines and three subdistal setae. Maxilla (Fig. 34F) with semi-triangular shape. Labium (Fig. 34G) lobes distolateral corner naked. Maxilliped (Fig. 34H) endites merged, with groove in the mid-length, distal margin with two tubercles (gustatory cusps) and seta; article-2 inner margin with three inner setae, outer margin with seta; article-3 with three setae; article-4 with five setae.

Cheliped (Fig. 35A) slender; basis 1.8 L:W; carpus 2.2 L:W, with two ventral setae, and with distal and subproximal dorsal setae; chela non-forcipate; palm 1.5 L:W, with row of three setae on inner side; fixed finger distal spine pointed, with three ventral setae; dactylus 6.5 L:W, proximal seta present.

Pereopod-1 (Fig. 35B) basis 9.1 L:W, with one ventral and two dorsal setae; merus 2.0 L:W, and 0.7x carpus; carpus 2.7 L:W, 0.5x propodus, with four setae; propodus 5.8 L:W, 0.9x dactylus and unguis combined length, with two setae; dactylus 0.6x unguis.

Pereopod-2 (Fig. 35C) basis 5.4 L:W, 1.9x merus, with ventral seta; ischium with ventral seta; merus 2.6 L:W, 0.8x carpus, with seta and spine; carpus 3.1 L:W, 0.8x propodus, with two simple setae, one regular spine and one blade-like spine 0.6x propodus; propodus 5.9 L:W, 1.5x as long dactylus and unguis combined length, with serrate distal seta; dactylus 0.6x unguis.

Pereopod-3 (Fig. 35D) basis 6.2 L:W, 4.9x merus; ischium with ventral seta; merus 0.7 L:W, 0.5x carpus, with seta; carpus 3.3 L:W, 1.1x propodus, with simple seta, regular spine and blade-like spine 0.7x propodus; propodus 4.7 L:W, 1.4x dactylus and unguis combined length, with serrate distal seta; dactylus 0.8x unguis.

Pereopod-4 (Fig. 35E) basis 7.3 L:W, 5.5x merus, with penicillate ventral seta; ischium with ventral seta; merus 1.5 L:W, 0.4x carpus, with two distal setae; carpus six L:W, 1.1x propodus, with one simple, one sensory, one regular spine and one blade-like spine (distally broken), rod seta 0.4x propodus; propodus 5.4 L:W, 2.7x dactylus and unguis combined length, with two ventral setae, one penicillate, and one serrate setae on dorsal margin 0.6x propodus; dactylus 1.5x unguis.

Pereopod-5 (Fig. 35F) carpus with two simple, one sensory 0.3x propodus, one blade-like spine 0.25x propodus; propodus 4.4 L:W, 3.1x dactylus and unguis combined length, with two ventral setae, one penicillate and one serrate dorsal seta 0.9x propodus; dactylus as long as unguis.

Pleopods (Fig. 35G) exopod with five, endopod with 10 plumose setae.

Uropod (Fig. 35H) exopod 1.1.x endopod, with two articles; article-1 5.0 L:W, with seta; article-2 4.2 L:W, with two setae. Endopod article-1 3.7 L:W, with one simple and two penicillate setae; article-2 4.0 L:W, with five simple and two penicillate setae;

**Distribution:** *P*. *oloughlini* n. sp. is known only from APEI3 of the Clarion and Clipperton Fractures Zone, Central Pacific.

**Remarks:** Uropod exopod longer than endopod separates *Pseudotanais oloughlini* from *P. abathagastor*, *P. corollatus*, *P. denticulatus*, *P. georgesandae*, *P. chopini* and *Pseudotanais* sp. C. *P. oloughlini* is most similar to *P. chaplini* but can be distinguished by its long blade-like spine on carpus of pereopods 2 and 3 (short blade-like spine in *P. chaplini*).

***Pseudotanais mariae*** **n**. **sp**.

Figures 36–38.

**Figure 36:**
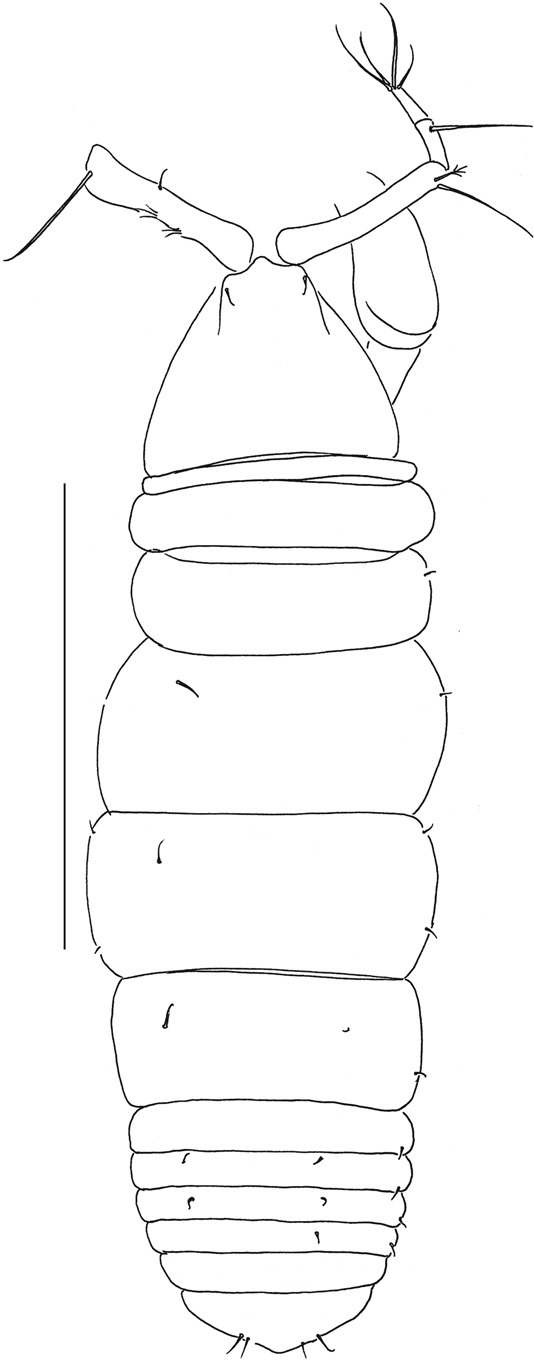
*Pseudotanais mariae* n. sp., ZMH K-56592, holotype neuter. Dorsal view. Scale bar: 1 mm.

**Figure 37:**
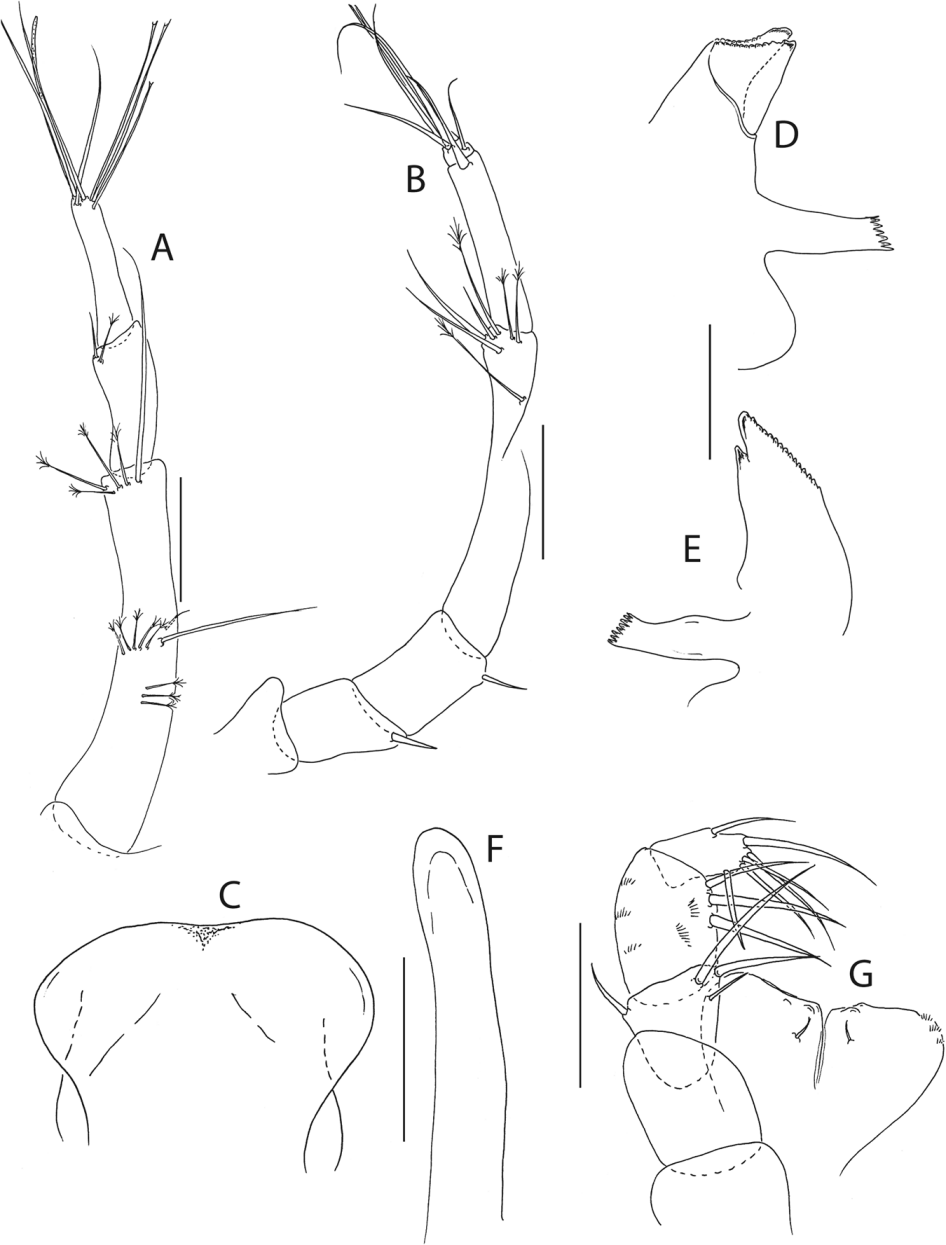
*Pseudotanais mariae* n. sp., ZMH K-56591, neuter. (**A**), antennule; (**B**), antenna; (**C**), labrum; (**D**), left mandible; (**E**), right mandible; (**F**), maxilliped, (**G**), epignath. Scale bar: 0.1 mm.

**Figure 38:**
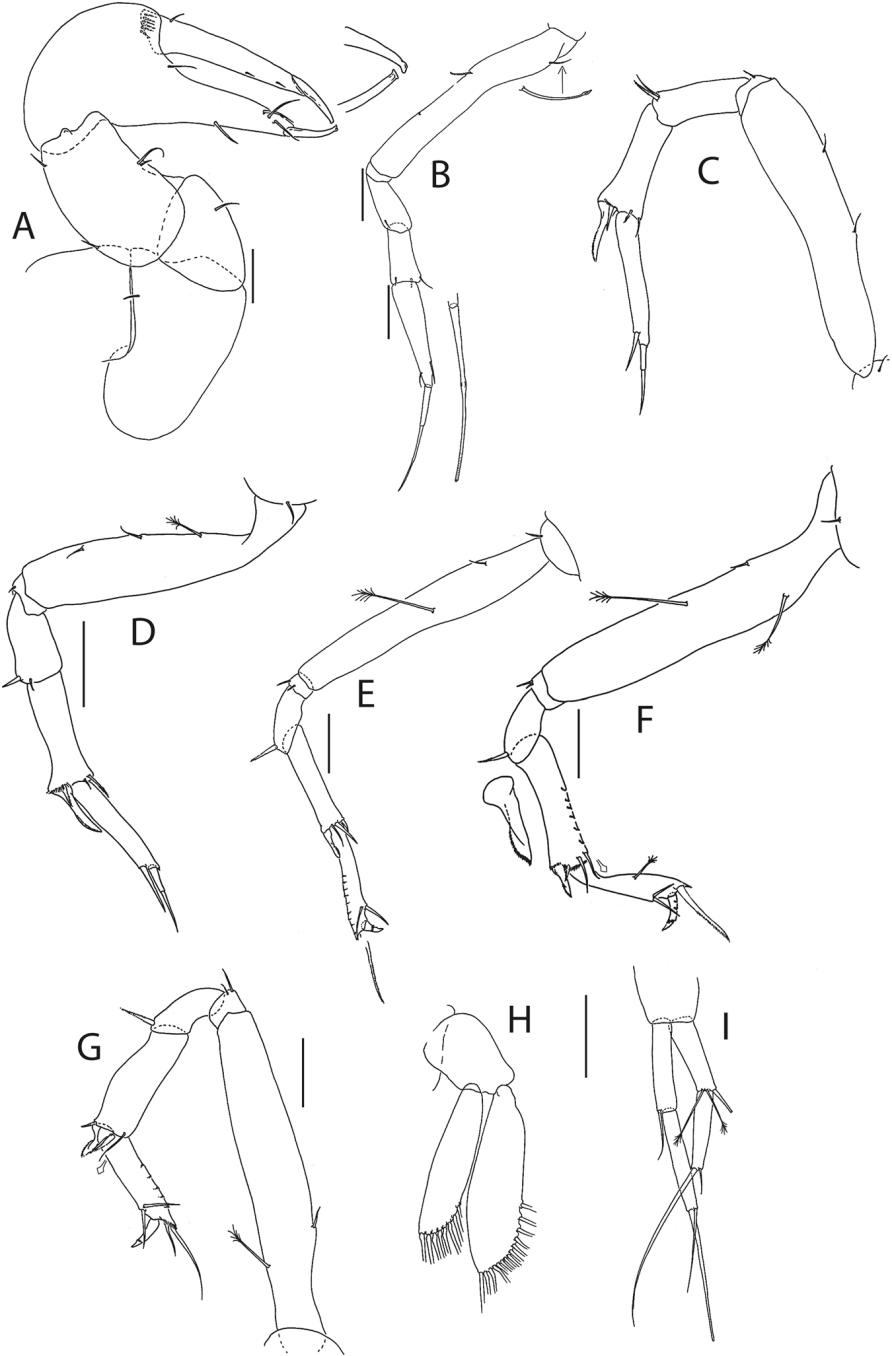
*Pseudotanais mariae* n. sp., ZMH K-56591, neuter. (**A**), cheliped; (**B**), pereopod-1; (**C**), pereopod-2; (**D**), pereopod-3; (**E**), pereopod-4; (**F**), pereopod-5; (**G**), pereopod-5; (**H**), pleopod; (**I**), uropod. Insets at (**F**,**G**) show detail of tip of the rod seta. Scale bar: 0.1 mm.

**Material examined:** Holotype: neuter, BL = 2.4 mm, ZMH K-56592. St. 81, 11° 3.97′N 119° 37.67′W, 4365 m, 1 Apr 2015.

Paratypes: neutrum, BL = 1.4 mm, ZMH K-56590. St. 20, 11° 49.81′N 117° 0.28′W, 4093 m, 22 Mar 2015; neuter, BL = 2 mm, ZMH K-56591 (dissected). St. 81, 11° 3.97′N 119° 37.67′W, 4365 m, 1 Apr 2015; neuter, BL = 1.9 mm, ZMH K-56593. St. 99, 11° 2.61′N 119° 39.52′W, 4401 m, 4 Apr 2015.

**Diagnosis**: Mandible molar wide. Antenna articles 2–3 with seta. Pereopod-2 carpus blade-spine short. Uropod exopod slightly shorter than endopod.

**Etymology:** The species is dedicated to Maria Jakiel, the mother of the first author.

**Description of neuter**. BL 2.4 mm. Body robust (Fig. 36), 3.2 L:W. Carapace 0.8 L:W, 9.0x pereonite-1, 0.2x BL. Pereonites 0.6x BL, pereonites-1–6: 0.1, 0.2, 0.3, 0.4, 0.4 and 0.4 L:W, respectively. Pleon short, 0.2x BL. Pleonites 0.7 L:W.

Antennule (Fig. 37A) article-1 0.5x total length, 5.8 L:W, 2.6x article-2, with two simple and eight penicillate setae at mid-length and one simple and four penicillate setae distally; article-2 2.2 L:W, 0.9x article-3, one simple and one penicillate setae distally; article-3 3.5 L:W, with three simple and three bifurcate setae, and aestetasc distally.

Antenna (Fig. 37B) article-2 1.5 L:W; 0.9x article-3, with seta 0.4x article-2; article-3 1.6 L:W, 0.4x article-4, with seta 0.4x article-3; article-4 5.0 L:W, 1.8x article-5, with penicillate subdistal seta, three simple and three penicillate setae distally; article-5 4.9 L:W, 8.5x article-6, with distal seta; article-6 0.7 L:W, with five setae.

Mouthparts. Labrum (Fig. 37C) hood-shaped, naked. Left mandible (Fig. 37D) *lacinia mobilis* well developed and serrate distally, incisor distal margin serrate, molar wide, with spines distally. Right mandible (Fig. 37E) incisor distal margin serrate, *lacina mobilis* merged to a small process. Maxilliped (Fig. 37F) endites merged, with groove in the mid-length, distal margin with two tubercles (gustatory cusps) and seta; palp article-2 inner margin with three inner setae, outer margin with seta; article-3 with three inner setae; article-4 with five inner distal and subdistal setae and one outer seta. Epignath (Fig. 37G) distally rounded.

Cheliped (Fig. 38A) robust; basis 1.7 L:W, with distoproximal seta; merus with seta; carpus 1.6 L:W, with two ventral setae, one distal and one subproximal seta dorsally; chela non-forcipate, palm 1.1 L:W, with row of five setae on inner side; fixed finger with three ventral setae and three inner setae, cutting edge almost simple; dactylus 7.0 L:W, cutting edge with two spines, proximal seta present.

Pereopod-1 (Fig. 38B) basis 7.3 L:W, with two simple ventral setae and sensory dorsal seta; merus 2.2 L:W and 0.9x carpus, with seta; carpus 2.4 L:W, 0.6x propodus, with three setae; propodus 4.0 L:W, 0.9x dactylus and unguis combined length, with two setae, dactylus 0.6x unguis.

Pereopod-2 (Fig. 38C) coxa with seta; basis 5 L:W, 3.5x merus, with two ventral seta; ischium with ventral seta; merus 2.4 L:W, 0.8x carpus, with two setae; carpus 3 L:W, 0.8x propodus, with two simple setae, one spine and one blade-like spine, 0.4x propodus; propodus 6.2 L:W, 2.1x dactylus and unguis combined length, with seta; dactylus 0.6x unguis.

Pereopod-3 (Fig. 38D) coxa with seta; basis 4.5 L:W, 3.2x merus, with two simple and one penicillate seta ventrally; ischium with two ventral setae; merus 2.1 L:W, 0.8x carpus, with two setae; carpus 3.1 L:W, with two simple setae, one spine and one blade-like spine, 0.5x propodus; propodus 5.7 L:W, 1.5x dactylus and unguis combined length, with seta; dactylus 0.7x unguis.

Pereopod-4 (Fig. 38E) basis 7.6 L:W, 4.4x merus, with penicillate ventral seta and simple dorsal seta; ischium with two ventral setae; merus 3 L:W, 0.6x carpus, with seta; carpus 4 L:W, 0.9x propodus, with one simple, one rod seta 0.3x propodus, one spine and one blade-like spine 0.3x propodus; propodus 5.2 L:W, 4.7x dactylus and unguis combined length, with two simple setae ventrally, one serrate seta dorsally 0.7x propodus and microtrichia on ventral margin; dactylus 3x unguis.

Pereopod-5 (Fig. 38F) basis 5.4 L:W, 7.7x merus, with one simple and one penicillate seta ventrally and with penicillate seta dorsally; ischium with two ventral seta; merus 5.2 L:W, 0.5x carpus, with seta; carpus 3.9 L:W, 1.3x propodus, one simple, one sensory 0.3x propodus, one spine and one blade-like spine, 0.3x propodus; propodus 4.0 L:W, 2.5x dactylus and unguis combined length, with two simple ventral seta and serrate dorsal seta 0.7x propodus; dactylus 0.1x unguis.

Pereopod-6 (Fig. 38G) basis 7.7 L:W, 4.9x merus, with simple seta ventrally and with penicillae seta dorsally; ischium with two ventral seta; merus 2.3 L:W, 0.6x carpus, with serrate seta; carpus 3.6 L:W, 1.2x propodus, with one simple, one sensory 0.3x propodus, one spine, and one blade-like spine 0.3x propodus; propodus 3.3 L:W, 2.5x dactylus and unguis combined length, with two setae on ventral margin and one serrate setae on dorsal margin 0.7x propodus and microtrichia on ventral margin; dactylus 3.0x unguis.

Pleopods (Fig. 38H) exopod with 8, endopod with 14 plumose setae.

Uropod (Fig. 38I) peduncle 0.8 L:W; exopod 0.6x as long as, with two articles; article-1 3.7 L:W, with one simple and two penicillate setae; article-2 five L:W, with two seta; endopod article-1 4.7 L:W, with one seta; article-2 6.7 L:W, with two setae.

**Distribution:** *P*. *mariae* n. sp. is known from the Belgium (GSR) and Interoceanmetal (IOM) licence areas of the Central Pacific.

**Remarks:** The presence of setae on antenna articles 2–3 distinguishes *P*. *mariae* from other members of the ‘denticulatus + abathagastor’ group (*P*. *abathagastor*, *P*. *corollatus*, *P*. *denticulatus*, *P*. *georgesandae*, *P. chopini*, *P*. *chaplini*, *P*. *oloughlini and Pseudotanais* sp. C), which have antenna articles 2–3 armed with spines.

$$ \mbox{'} {\rm{spicatus}}\mbox{'}\,{\rm{group}}$$

**Diagnosis**: Mandible molar acuminate or wide. Antenna articles 2–3 armed with spine. Pereopod-1 basis with setae on ventral margin. Pereopod-1 merus and carpus distodorsal seta short. Pereopod-2 carpus blade-like spine short. Pereopod 5–6 carpus distodorsal seta short. Uropod slender, exopod slightly shorter or equal to endopod.

**Species included**: *Pseudotanais spicatus* Bird & Holdich, 1989; *P. tympanobaculum* Błażewicz-Paszkowycz, Bamber & Cunha, 2011; *P*. *kobro* n. sp.

**Remarks**: The presence of a very short blade-like spine on carpus of pereopod-2 allows to distinguish this group from other taxa.

***Pseudotanais kobro*** **n**. **sp**.

Figures 39 and 40.

**Figure 39:**
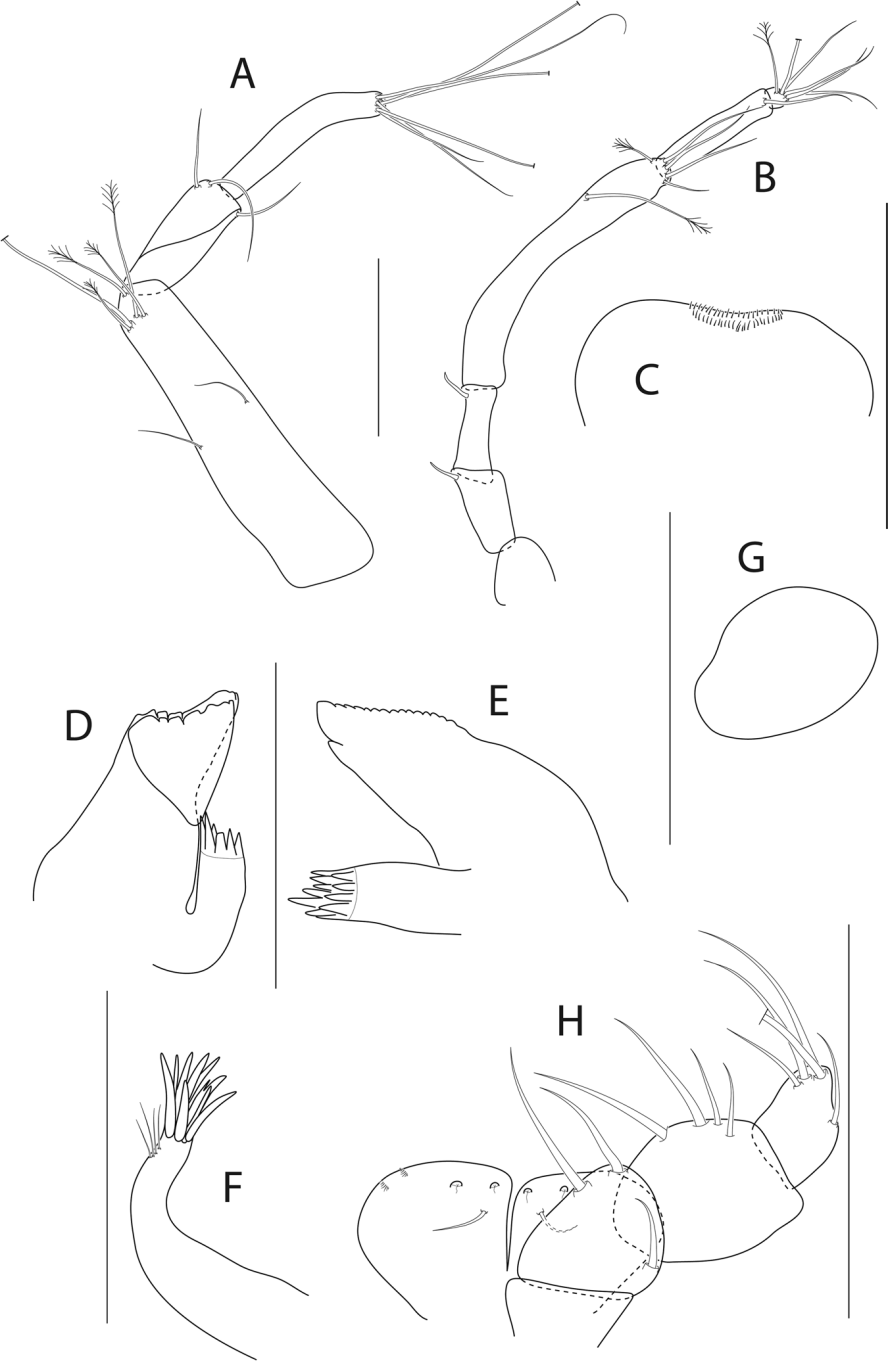
*Pseudotanais kobro* n. sp., ZMH K-56587, neuter. (**A**), antennule; (**B**), antenna; (**C**), labrum; (**D**), left mandible; (**E**), right mandible; (**F**), maxillula, (**G**), maxilla, (**H**), maxilliped. Scale bar: 0.1 mm.

**Figure 40:**
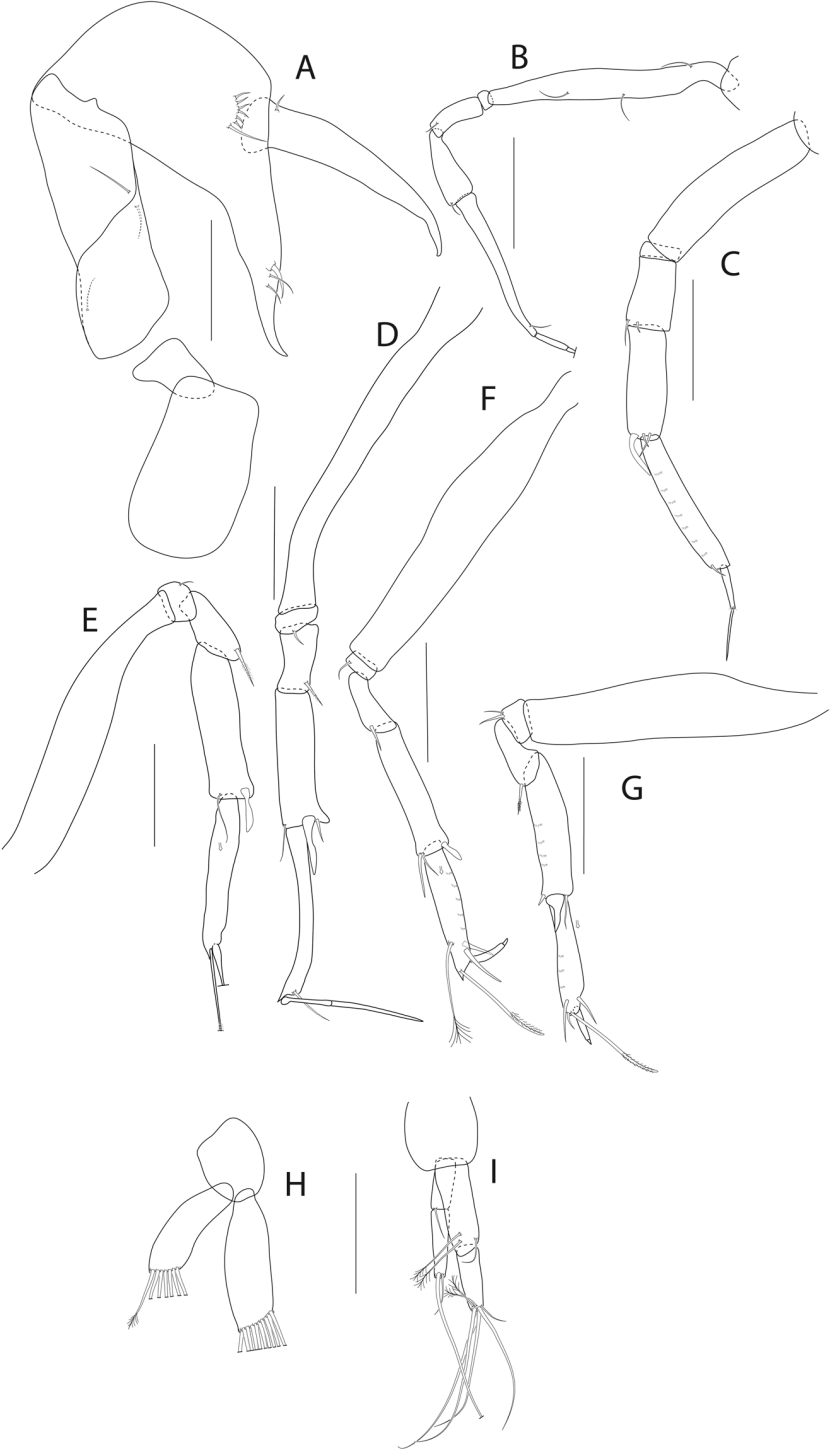
*Pseudotanais kobro* n. sp., ZMH K-56586 (D, E), ZMH K-56587 (**A**–**C**,**F**–**H**), neuter. (**A**), cheliped; (**B**), pereopod-1; (**C**), pereopod-2; (**D**), pereopod-3; (**E**), pereopod-4; (**F**), pereopod-5; (**G**), pereopod-6; (**H**), pleopod; (**I**), uropod. Insets at (**E**–**G**) show detail of tip of the rod seta. Scale bar: 0.1 mm.

**Material examined:** Holotype: neuter, BL = 1.3 mm, ZMH K-56589. St 117, 13° 52.39′N 123° 15.30′W, 4496 m, 7 Apr 2015.

Paratypes: neuter, BL = 1.4 mm, ZMH K-56585 (partly dissected). St 11° 3.97′N 119° 37.67′W, 4365 m, 1 Apr 2015; three neuters BL = 1.3–1.4 mm, ZMH K-56586 (dissected), ZMH K-56587 (dissected), ZMH K-56588. St 99, 11° 2.61′N 119° 39.52′W, 4401 m, 4 Apr 2015.

**Diagnosis:** Antenna articles 2–3 with a thin and long spine, unguis of pereopod 5**–**6 minute.

**Etymology**: The name of the species is dedicated to Katarzyna Kobro, a modern Polish sculptor.

**Description**. Antennule (Fig. 39A) article-1 0.5x total length, 4.2 L:W, 2.5x article-2, with two simple setae in mid-length, one simple and four penicillate setae distally; article-2 2.5 L:W, 0.8x article-3, with three setae; article-3 5.3 L:W, with six setae (three broken).

Antenna (Fig. 39B) article-2 2.1 L:W; article-2 0.9x article-3, with spine 0.3x article; article-3 2.8 L:W, 0.3x article-4, with spine, 0.3x article; article-4 6.2 L:W, 2.5x article-5, one penicillate seta in mid-length, four simple setae and one penicillate seta distally; article-5 5 L:W, 5x article-6, with distal seta; article-6 wide, one penicillate seta and 5 simple setae (one broken).

Mouthparts. Labrum (Fig. 39C) hood-shaped, setose. Left mandible (Fig. 39D) *lacinia mobilis* well developed and serrate distally, incisor distal margin serrate, molar wide. Right mandible (Fig. 39E) incisor distal margin serrate, *lacina mobilis* merged to a small process. Maxillule (Fig. 39F) with 8 simple and one bifurcate distal spine with four subdistal setae. Maxilla (Fig. 39G) oval. Maxilliped (Fig. 39H) endites merged, with groove in the mid-length, distal margin, with two tubercles and one seta; palp article-2 inner margin, with two setae, outer margin with seta; article-3 with four setae; article-4 with four inner distal and subdistal setae and one outer seta.

Cheliped (Fig. 40A) slender; basis 1.6 L:W; carpus 3 L:W, with two ventral setae, subproximal seta; chela non-forcipate; palm 1.2 L:W, row of 6 serrate setae on inner margin; fixed finger distal spine pointed, 1.4x palm, with three ventral setae; dactylus 7.5 L:W, cutting edge smooth, proximal seta present.

Pereopod-1 (Fig. 40B) basis 8.8 L:W, with one seta ventrally and two setae dorsally; merus 2.5 L:W and 0.8x carpus, with seta; carpus 2.6 L:W, 0.4x propodus, with seta; propodus 10 L:W, with seta.

Pereopod-2 (Fig. 40C) basis 4.5 L:W, 2.6x merus; merus 1.7 L:W, 0.6x carpus, with two setae; carpus 2.7 L:W, 0.8x propodus, with two simple setae and blade-like spine, 0.3x propodus; propodus 5.6 L:W, 1.5x dactylus and unguis combined length, with simple seta and microtrichia on ventral margin; dactylus 0.8x unguis.

Pereopod-3 (Fig. 40D) basis 7 L:W, 5.8x merus; ischium with simple seta; merus 2 L:W, 0.5x carpus, with one serrate setae; carpus 3.7 L:W, 0.7x propodus, with two simple setae and blade-like spine, 0.3x propodus; propodus 7 L:W, 1.3x dactylus and unguis combined length, with serrate seta; dactylus 0.5x unguis.

Pereopod-4 (Fig. 40E) basis 5.8 L:W, 3.9x merus; ischium with simple seta; merus 2.1 L:W, 0.5x carpus, with one serrate setae; carpus 3.7 L:W, 1x propodus, with one simple setae and blade-like spine, 0.3x propodus; propodus 6 L:W, with one serrate seta; unguis broken.

Pereopod-5 (Fig. 40F) basis 6.7 L:W, 5.6x merus; ischium with ventral seta; merus 3 L:W, 0.6x carpus, with one serrate seta; carpus 4.2 L:W, 0.9x propodus, one simple seta, one rod seta and one blade-like spine, 0.2x propodus, rod seta 0.4x propodus; propodus 5.7 L:W, 2.9x dactylus and unguis combined length, with two simple ventral setae and one dorsal serrate seta 0.7x propodus and microtrichia on ventral margin, dactylus 7x unguis.

Pereopod-6 (Fig. 40G) basis 5.8 L:W, 4.8x merus; merus 2.4 L:W, 0.5x carpus, with one serrate seta; carpus 4.2 L:W, 1.1x propodus, with one simple seta, one rod seta and one blade-like spine, 0.3x propodus, rod seta 0.3x propodus; propodus 5.7 L:W, 3.3x dactylus and unguis combined length, with two ventral and one serrate dorsal seta 0.9x propodus; dactylus 6x unguis.

Pleopods (Fig. 40H) exopod with seven and endopod with 10 plumose setae, respectively.

Uropod (Fig. 40I) peduncle 1.1 L:W, exopod with two articles; article-1 2.5 L:W, with seta; article-2 4.3 L:W, with two setae; endopod article-1 2.8 L:W, with one simple and two penicillate setae; article-2 3.7 L:W, with two penicillate and five simple setae. Exopod 0.8x endopod.

**Distribution:** *P*. *kobro* n. sp. is recorded from is known from the Belgium (GSR), German (BGR) and Interoceanmetal (IOM) licence areas of the Central Pacific.

**Remarks:** *Pseudotanais kobro* n. sp. can be distinguished from the other members of the ‘spicatus’ group by the presence of a thin, long spine on antenna article 2–3. Besides, the new species has wide mandible molar (being acuminate in *P*. *spicatus* and *P*. *tympanobaculum*) and it can be further distinguished from *P*. *spicatus* by having an endopod of uropod composed of two articles (one article in *P*. *spicatus*). Finally, *P*. *kobro* has a short, minute unguis on pereopod 5–6, differing it from the elongated unguis of *P*. *tympanobaculum*.

#### Identification keys to pseudotanaids found within the CCZ

**Key for Pseudotanaidae genera (modified from Bird & Holdich 1989 and McLelland 2008** **)**

1. Pereopods 2 and 3 blade-like spine on carpus
  present (Fig. 16D)………………………………….……………………………………………………2
  absent (see Larsen *et al*. (2012); Fig. 10C ^34^)………..……………………………………*Akanthinotanais*
2. Number of ventral setae on fixed finger (pollex) of chela
  one (Fig. 6A)…………………………………………………………………………………………….3
  two (see Bird & Holdich (1989); Fig. 23J ^30^)………………………………………….....*Parapseudotanais*
3. Inner margin of pollex (fixed finger)
  serrated (Fig. 6A)………………………………………………………………………………………..4
  smooth (Fig. 16A)……………………………………………………………………….….*Pseudotanais*
4. Proportion of the length of pereonite-1 to 2 (S = < 0.4; L = > 0.75). Profile of the thick rod seta on antennular article-3, antennal article-6 and maxilliped palp article-4 (0 = absent; 1 = present)

S-0-1-1 (see Jakiel *et al*. (2018); Fig. 5A,B,H ^31^)………………………….….………….… *Mystriocentrus*

L-1-0-0 (Fig. 5A)……………………….……………………………………..………. *Beksitanais* n. gen.

**Key for** ***Pseudotanais*** **morpho-groups**

1. Forcipate chela
  present (Jakiel *et al*. (2015); Fig. 15A ^35^)…………….…………………………………………‘forcipatus’
  absent (Fig. 16A)……………………………………………………………………………………….. 2
2. Uropod exopod
  short (≤½ endopod) (see Bird & Holdich (1989); Fig. 3H ^30^).………………………….………..‘colonus’
  long (>½ of endopod) (Fig. 16H)………………………………………………………………………. 3
3. Pereopod-1 merus seta
  long (≥½ of merus) (Fig. 16B)……………………………………….…………... ‘affinis + longisetosus’
  short (≤½ of merus) (Fig. 35B)………………………..…………………………………..………….…4
4. Pereopod-5 and 6 unguis

minute (Fig. 40F)……………………………………………………………… ‘spicatus’ (*P*. *kobro* n. sp.)

elongated (Fig. 30F)……………………………………………….…….… ‘denticulatus + abathagastor’

**Key to ‘affinis + longisetosus’ species**

1. Pereopod-5 and 6 carpus dorsodistal seta
  short (0.3x propodus) (Fig. 25E)…………………………………...……………………………………2
  long (≥0.8x propodus) (Fig. 19E)…………………………………………………………………....… 3
2. Pereopod-1 merus distal seta
  1x merus (Fig. 25B)………………………………………………………..……….…….. *P*. *geralti* n. sp.
  0.5x merus (Fig. 22B)…………………………………………………..……….……. *P*. *yenneferae* n. sp.
3. Pereopod-1 basis
  few setae (1-3) (Fig. 11B)……………………………………………………………………….…….… 4
  many setae (5-6) (Fig. 16B) …………………………. …………………………………………………5
4. Pereopod- 3 with blade-like spine
  semilong (0.5x propodus) (Fig. 11D) …………………………………………..……….. *P*. *uranos* n. sp.
  long (≥0.6x propodus) (Fig. 13D) ……………………………………………….……….. *P*. *gaiea* n. sp.
5. Maxilliped endite

naked (Fig. 18F) ……………………………………………………………………….… *P*. *romeo* n. sp.

with two tubercles (Fig. 15H) ………………………………………….…….…………. *P*. *julietae* n. sp.

**Key to ‘denticulatus + abathagastor’ species**

1. Antenna article 2 and 3 with
  spine (Fig. 34B) ………………………………………………………………………………….…….. 2
  seta (Fig. 37B) ……………………………………….……….………………….……… *P*. *mariae* n. sp.
2. Uropod exopod length
  ≤1x endopod (Fig. 38I) …………………………….………………………………………………….. 3
  >1x endopod (Fig. 32H) …………………………….………………………………………………… 4
3. Mandible molar
  wide (Fig. 26C) …………………………………………………………………….. *P*. *georgesande* n. sp.
  acuminate (Fig. 29E) ……………………………………………………………………. *P*. *chopini* n. sp.
4. Pereopod-3 blade-like spine

short (0.3x propodus) (Fig. 32D) …………………………………………………….… *P*. *chaplini* n. sp.

long (0.6x propodus) (Fig. 35D) …………………………………………….…….….. *P*. *oloughlini* n. sp.

## Discussion

The present study uncovered a significant diversity of pseudotanaids within the CCZ. A total of 15 new species are described here combining morphological and molecular data. Pseudotanaidae had been reported only once before from CCZ and without including any description^36^. This is also the first time pseudotanaids are studied using a DNA barcoding approach, with the only entry available in GenBank for this family being the histone 3 sequence from a *Pseudotanais* sp. collected in Crawl Key, Panama^27^. Another study on Pseudotanaidae from the North Atlantic reported a complex of cryptic species in four ecologically-diverse basins around Iceland^31^, although the lack of genetic data prevented clear taxa delimitation. The wide geographic sampling carried out, combined with a reverse taxonomy approach, suggests that pseudotanaids might have comparatively narrow ranges (considering the entire study area), because most species were mainly limited to the closest stations. Potentially narrow ranges could also be inferred from the extensive tanaid collection made in Amundsen and Scotia Seas^29^. Deep-sea species are generally rare and sparsely distributed, so it is not surprising that each species in our study was represented by just a few individuals. The mechanisms maintaining the immense diversity but low abundances in the deep sea are hardly understood^29^ and the low number of properly preserved individuals obtained, despite immense logistic efforts, hampers morphological and molecular studies of the abyssal fauna^37,38^.

Resolving the presence of cryptic species is currently considered one of the main challenges for taxonomy^39–41^. Phenotypic plasticity and high sexual dimorphism may lead to misidentification of tanaidaceans^42,43^ and lack of detailed morphological studies might obscure the real number of species and true diversity^44,45^. For example, dimorphic male and females of *Beksitanais apocalyptica* could be described for the first time here thanks to a DNA barcoding approach. *Beksitanais apocalyptica* is the only member of the genus described from the Pacific and the first for which molecular information is made available. The new genus is distinguished from the other Pseudotanaidae genera based on the following set of unique characters or character combination: Antennula article-3 with thickened rod seta; chela forcipate with serrate incisive margin, but propodus (palm) without small folds in distodorsal corner and pereopods 4-6 dactylus and unguis fused with a small hook on tip. Similarly, the separation of the known *Pseudotanais* species into the four groups proposed by Bird & Holdich^32^ and Jakiel *et al*. namely, ‘affinis’, ‘denticulatus’, ‘forcipatus’ and ‘longisetosus’ was re-assessed here. Careful examination of the material from CCZ uncovered a close relationship between ‘affinis’ and ‘longisetosus’ and the presence of at least two more *Pseudotanais* species groups namely, ‘abathagastor’ and ‘spicatus’. The recognition of these clades is supported by the setation pattern on pereopods 1, 5 and 6 and by the setal types on pereopods 2 and 3. The new ‘spicatus’ group can be characterized by very short blade-like spine in pereopod-2 and minute unguis in pereopods 5 and 6, whereas the ‘abathagastor’ group is distinguished by a combination of short setae on merus and carpus of pereopod 1, and by the presence of setae (not spines) on the antennal articles 2 and 3. The congruence observed for both morphological and molecular data suggests that *Pseudotanais* might in fact be formed by several complexes of cryptic species.

Discovering new taxa in a sample taken from any arbitrary chosen spot in the deep sea occurs quite frequently^46^. The deep-sea has traditionally been associated with a homogeneous environment, but state-of-the-art technologies proved that abyssal landscapes include different structures, such as seamounts, rises or fracture zones. This spatial heterogeneity is likely to impact the diversity and distribution of abyssal fauna, particularly for small epibenthic species^47^. The numerous asymmetric ridges, scarps, and elongate depressions at the Clarion facture zone can effectively limit dispersion and constitute geographical barriers, because none of the species collected from the APEI3 zone was found anywhere else. The Clarion Fracture Zone has been produced by seafloor spreading as the scar of transform faulting that began at least 80 million years ago and that is still continuing at present^48^. The patterns of magnetic intensity of the seafloor rocks in the studied area are displaced laterally, and rocks of the northern block are millions of years older than adjacent rocks south of the fracture zone^49^. Similarly, the elevated topography of the south-to-north ridge could be considered a remnant of an old east Pacific rise (EPR), a sea-floor spreading center that was active approximately 30 mya. Our results suggest that physical barriers restrict the distribution of Pseudotanaidae species, promoting genetic differentiation and allopatric speciation. The sessile lifestyle of pseudotanaid females, which are generally found in self-constructed tubes, makes them particularly sensitive to geographic barriers^44^.

Other environmental factors could explain the observed distribution of pseudotanaid taxa, and might be correlated with the CCZ deep sea landscape. There is mineralogical and chemical evidence for heterogenous sediment composition due to hydrothermal influence around the Clarion fracture zone between 113°W and 119°W. Similarly, nodules from pelagic clays found north of the Clarion fracture zone show higher Mn/Fe ratios^50^. Food availability might also affect the spatial distribution of diversity in the deep-sea^50^, because only a small part of the particulate organic carbon (POC) from the euphotic zone will ever reach the ocean bottom^16^. Megafauna studies suggest higher abundance and diversity in the eastern part of CCZ, where POC availability is larger^37^. For example, Polychaeta family richness was found to be higher in the eastern IOM area than in the more western IFREMER region^43^. Nevertheless, the northernmost area studied here (APEI3) showed similar Pseudotanaidae abundances and species richness as the southeastern areas despite a gradual increase in POC flux. Finally, other factors such as the calcite compensation depth (CCD), which in the Pacific Ocean is about 4200–4500 metres, could also have an impact on the carapace-bearing crustaceans^16^. Further sampling within the CCZ would be essential to properly evaluate the relative importance of these factors on the observed distribution of deep-sea pseudotanaids.

The Clarion-Clipperton Zone remains the focus of international mining companies and faces a real danger of industrial exploitation, so recognizing its biological diversity and how it is structured are primary and critical steps preceding any potential anthropogenic activity^51,52^. A marginal understanding of deep-sea ecosystems utterly prevents an adequate assessment of the potential impact of mining operations on the marine environment^53^. Deep-sea expeditions are generally deprived of an opportunity for repeated sampling, being highly costly and burdened with logistic difficulties, so the large collection of pseudotanaids studied here is extremely valuable. The correlation observed between spatial features and species distribution has important implications for the establishment of protected areas, and the APEI3 area studied here would only protect one third of the total pseudotanaid species found in CCZ. It is possible that some species might have wider ranges than suggested by our current sampling, but this study represents an important first step in characterizing the diversity and distribution of pseudotanaids from the Tropical Eastern Pacific.

## Material and Methods

### Sampling

The European Joint Project Initiative – Oceans (JPI-O) ‘Ecological Aspects of the Deep-Sea Mining’ is a long-term intergovernmental initiative to assess the potential impact of deep sea mining using ecological and genetic techniques^54,55^. The marine expedition ‘EcoResponse 2015’ was organized to assess the genetic connectivity between populations from different CCZ areas. The biological material included in the present study was collected during SO-239 cruise, conducted on *RV Sonne*, from 10^th^ March until 30^th^ April 2015. Tanaidacean samples were taken from the Belgian, German and French license areas, but also from the APEI3 and Interoceanmetal (i.e. the consortium associating Bulgaria, Cuba, Czech Republic, Poland, Russian Federation and Slovakia). Thus, the areas surveyed include APEI3 (Areas of Particular Environmental Interest 3); BGR (Bundesanstalt fur Geowissenschalfen und Rofstoffe, Germany); IOM (Interoceanometal Joint Organisation); GSR (Global Sea Mineral Resources NV, Belgium) and IFREMER (France) (Table 1). An epibenthic sled (EBS) was used to collect material at each sampling site as in Brandt and Barthel^56^. Samples were sieved on board through a 300 µ mesh using cooled seawater and rapidly transferred to cold 96% EtOH. Fixed samples were stored at −20 °C until further processed. Detailed onboard and laboratory sample-processing procedures can be found in Rhiel^57^.

### Phylogenetic analyses

A single cheliped was taken using sterile needles as starting material for DNA extraction using the Chelex (InstaGene Matrix, Bio-Rad) method as in Palero *et al*.^58^. The COI gene was amplified using a 25 μL volume reaction containing 22 μL H_2_O, 0.5 μL of each primer (10 pmol/μL) polyLCO and polyHCO^59,60^ 1U of Illustra PuReTaq Ready−To−Go PCR Beads (GE Healthcare) and 2 μL of DNA template. The PCR protocol was 94 °C for 3 min, 40 cycles of 94 °C for 40 s, 42 °C for 30 s, 72 °C for 1 min, and a final elongation step of 72 °C for 10 min. A 2 μL aliquot of the PCR products was visualized in Midori Green-stained (Nippon Genetics) 1.5% agarose gels to verify PCR product quality and length. PCR purification and sequencing using forward and reverse primers was carried out by MACROGEN (Amsterdam, Netherlands). Consensus sequences were built using Geneious version 9.1.3 (www.geneious.com) and compared with the GenBank database using BLAST^61^ to discard contamination from non-arthropod sources. Sequences were aligned using alignment option (L-INSi) of MAFFT^62^ as implemented in Geneious. To improve reliability, we extracted conserved (ungapped) blocks of sequence from the alignment by using Gblocks server with default settings^63,64^. Selection of the best nucleotide substitution model was performed according to the BIC criterion as implemented in MEGA v7^58,65^. The aligned sequences and selected evolutionary model were used to estimate genetic distances and the corresponding Maximum Likelihood phylogenetic tree in MEGA. Initial trees for the heuristic search were obtained automatically by applying Neighbor-Join and BioNJ algorithms to a matrix of pairwise distances estimated using the Maximum Composite Likelihood (MCL) approach, and then selecting the topology with superior log likelihood value. Nodal support was assessed using 500 bootstrap replicates.

### Spatial modelling and genetic gradients

A 3D-model of the deep sea landscape of the CCZ was built using the GeoElevationData function as implemented in the *Mathematica* v11.0 software package (Wolfram Inc., USA). GeoElevationData returns the elevation with respect to the geoid (=mean sea level) of a specified location. An array including the bathymetry for 12,231 different latitude longitude coordinates was built by uniformly recording the mean sea level every 1/10^th^ of a decimal degree in the rectangular area spanning from 11°N 116°W to 19°N 131°W. A contour-plot representing the array of mean sea level values and the location of the sampling sites was generated using the ListPlot and ListContourPlot functions in *Mathematica*. Names for particular structures, including fractures, seamounts and knolls, are taken from the General Bathymetric Chart of the Oceans (GEBCO) undersea feature Gazetteer (https://www.ngdc.noaa.gov/gazetteer/). The degree of association between geographic and genetic distances was measured using the Spearman rank correlation. This non-parametric correlation test was selected because it does not carry any assumptions about the distribution of the data. A standard isolation by distance (IBD) analysis was also carried out in *Mathematica* to further analyze the presence of a linear correlation between geographic and genetic distances.

### Morphological analyses and species descriptions

Specimens were dissected with chemically-sharpened tungsten needles, and the dissected appendages slide-mounted using glycerine. Drawings were prepared using a light microscope (Nikon Eclipse 50*i*) equipped with a *camera lucida*. Digital drawings were obtained using a graphic tablet following Coleman^66^. Total body length (BL) was measured along the main axis of symmetry, from the frontal margin to the end of the telson. Body width (BW) was measured at the widest point along the main axis of symmetry. To simplify species descriptions, the expression ‘*N*x’ replaces ‘*N* times as long as’ and ‘*N* L:W’ replaces ‘*N* times as long as wide’. The measurements were made with a camera connected to the microscope (Nikon Eclipse Ci-L) and NIS-Elements View software (www.nikoninstruments.com). The body width and the length of the carapace, pereonites, pleonites, and pleotelson were measured on whole specimens. The poor condition of individuals after DNA extraction or incompleteness even for well-preserved specimens, made the description of pereonite and pleonite setation not reliable. Therefore, this character was not included in the species description. The morphological terminology here follows Błażewicz-Paszkowycz *et al*. (2012)^67^. The unique blade-like spine of *Pseudotanais*, *Mystriocentrus* and *Parapseudotanais* species^67^, is recognized as ‘long’ when is at least 0.6x propodus, ‘semilong’ when it is 0.5x propodus and ‘short’ when it is at most 0.3x the propodus. The type of sensory seta present on carpus of pereopod 4-6 is defined as rod seta (slightly inflated distally and with a pore) following^68^ and^69^. This seta is recognized as ‘long’ when is at least 0.8x propodus, ‘semilong’ when it is 0.5x propodus and ‘short when it is at most 0.25x propodus. Beside simple setae (=without ornamentation), at least four setae types are recognized here: (1) serrate – with serration or denticulation, (2) plumose – with any type of plumose or delicate setulae tufts distributed along the main axis, (3) penicillate – with a tuft of setules located distally and with a small knob on which a seta is fixed to the tegument and, (4) sensory – specified above.

Among the studied individuals: manca, neuter, and male stages were recognized. Specifically, the term ‘manca’ describes juveniles with or without buds of pereopod-6, respectively; ‘mature (swimming) male’^30^ refers to individuals with completely developed sexual dimorphic characters. ‘Neuter’ is retained for the stage developed from manca that cannot be classified as either female or juvenile male. The examined material will be deposited in “Senckenberg Research Institute and Natural History Museum” (Hamburg, Germany). Taxonomic descriptions and the corresponding identification key were prepared using the DELTA software (DEscription Language for TAxonomy)^44,66,70^.
